# Highly enantioselective access to diketopiperazines *via* cinchona alkaloid catalyzed Michael additions†
†Electronic supplementary information (ESI) available: Experimental procedures, analytical data and NMR spectra, X-ray data. CCDC 977363, 977364, 977365 and 1017592. For ESI and crystallographic data in CIF or other electronic format see DOI: 10.1039/c4sc03218g


**DOI:** 10.1039/c4sc03218g

**Published:** 2014-11-28

**Authors:** Alejandro Cabanillas, Christopher D. Davies, Louise Male, Nigel S. Simpkins

**Affiliations:** a School of Chemistry , University of Birmingham , Edgbaston , Birmingham , B15 2TT UK; b AstraZeneca , Innovative Medicines , Alderley Park , SK10 4TG UK

## Abstract

Michael addition reactions of triketopiperazine (TKP) derivatives to enones, mediated by a cinchona alkaloid-derived catalyst, deliver products in high yield and enantiomeric ratio (er). Use of unsaturated ester, nitrile or sulfone partners gives bridged hydroxy-diketopiperazine (DKP) products resulting from a novel Michael addition–ring closure.

## Abstract 2

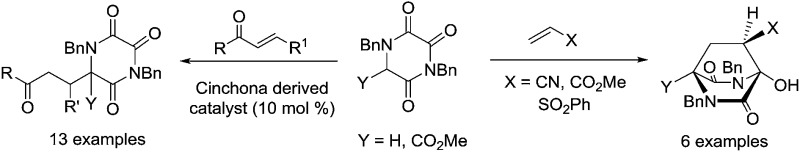
Alkaloid catalysed additions to triketopiperazines gives products in high yield and er (88 : 12 to 99 : 1), including bridged hydroxy-DKPs *via* Michael-addition–ring closure.

## Introduction

**Fig. 1:**
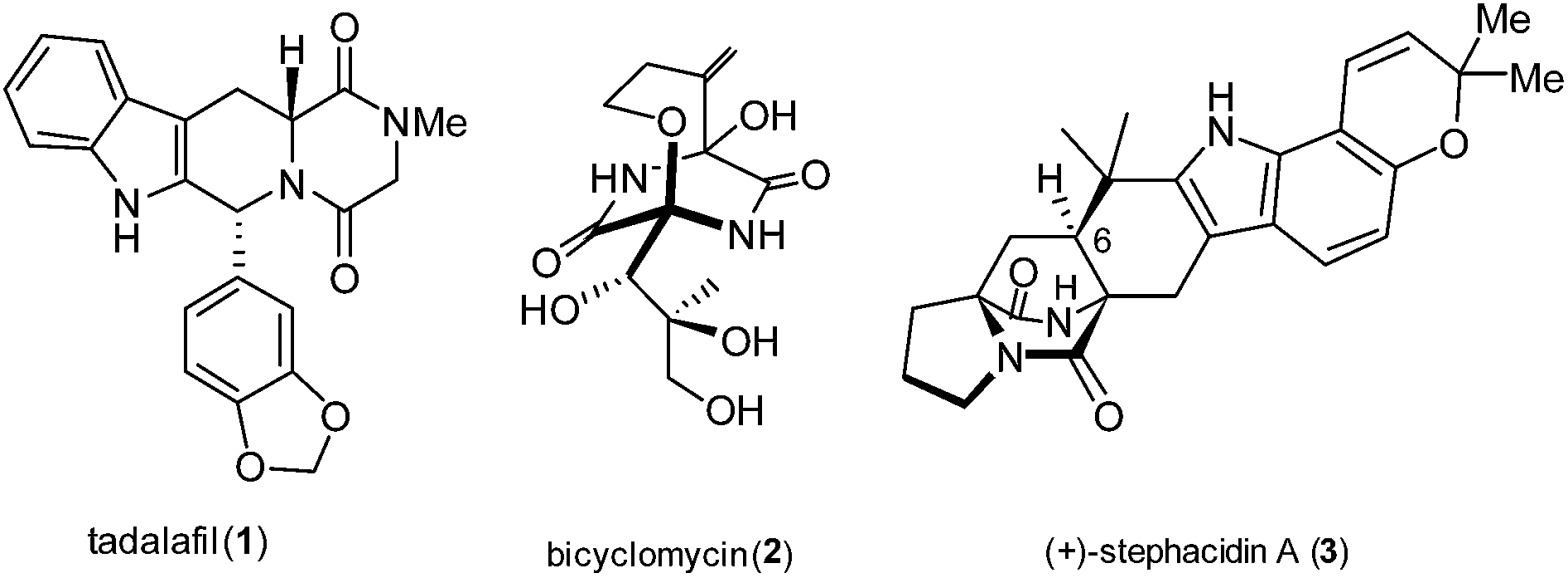
Structures of significant bioactive DKPs.

Diketopiperazines (DKPs) have acquired the status of privileged structures for medicinal chemistry, for example tadalafil (or cialis®, **1**) is a potent PDE5 inhibitor used for treating male erectile dysfunction, Fig. 1.^1^

The DKP structure is also prevalent in many types of peptide antibiotics, including the clinically significant compound bicyclomycin (**2**), which has an interesting [4.2.2] ether-bridged core.^2^

Many other important natural product families incorporate DKP motifs, for example the bicyclo[2.2.2]diazaoctane core structure features in the fungal metabolite stephacidin A (**3**), which displays potent and selective antitumour activity.^3^ The extensive studies of the synthesis and medicinal chemistry of DKPs and their occurrence in bioactive natural products have been described in a comprehensive review by Borthwick.^4^

Despite this background, access to enantiomerically pure DKPs has relied very heavily on chiral pool materials and, with the exception of studies aimed at stephacidins and related natural products, reports of stereocontrolled access to α,α-disubstituted systems are scarce.^5^

Herein, we describe highly enantioselective access to α,α-disubstituted DKPs, including bridged products related to **2** and **3**, by means of asymmetric Michael additions mediated by cinchona alkaloid catalysts.^6^ To date this field of organocatalysis has largely been limited to doubly activated Michael donors, such as malonates, β-diketones, and similarly acidic substrates,^7^ and the use of amidic donors has been scarcely explored.^8^ Key to our new chemistry is the finding that the under-explored triketopiperazine (TKP) motif provides exceptional activation for deprotonation, whilst also enabling facile access to DKP products through nucleophilic addition.^9^

## Results and discussion

Both the doubly activated α-carboxymethyl TKP **4a**, and the parent system **4b**, were readily prepared using an established approach.9b,^10^ Mindful of the challenge of engaging masked glycine **4b** in this type of chemistry, initial screening focused on the doubly activated TKP **4a**, using a limited range of alkaloid catalysts **5a–e**, and with MVK as Michael acceptor, Table 1.

**Table 1:** Initial catalyst screening using TKP **4a**

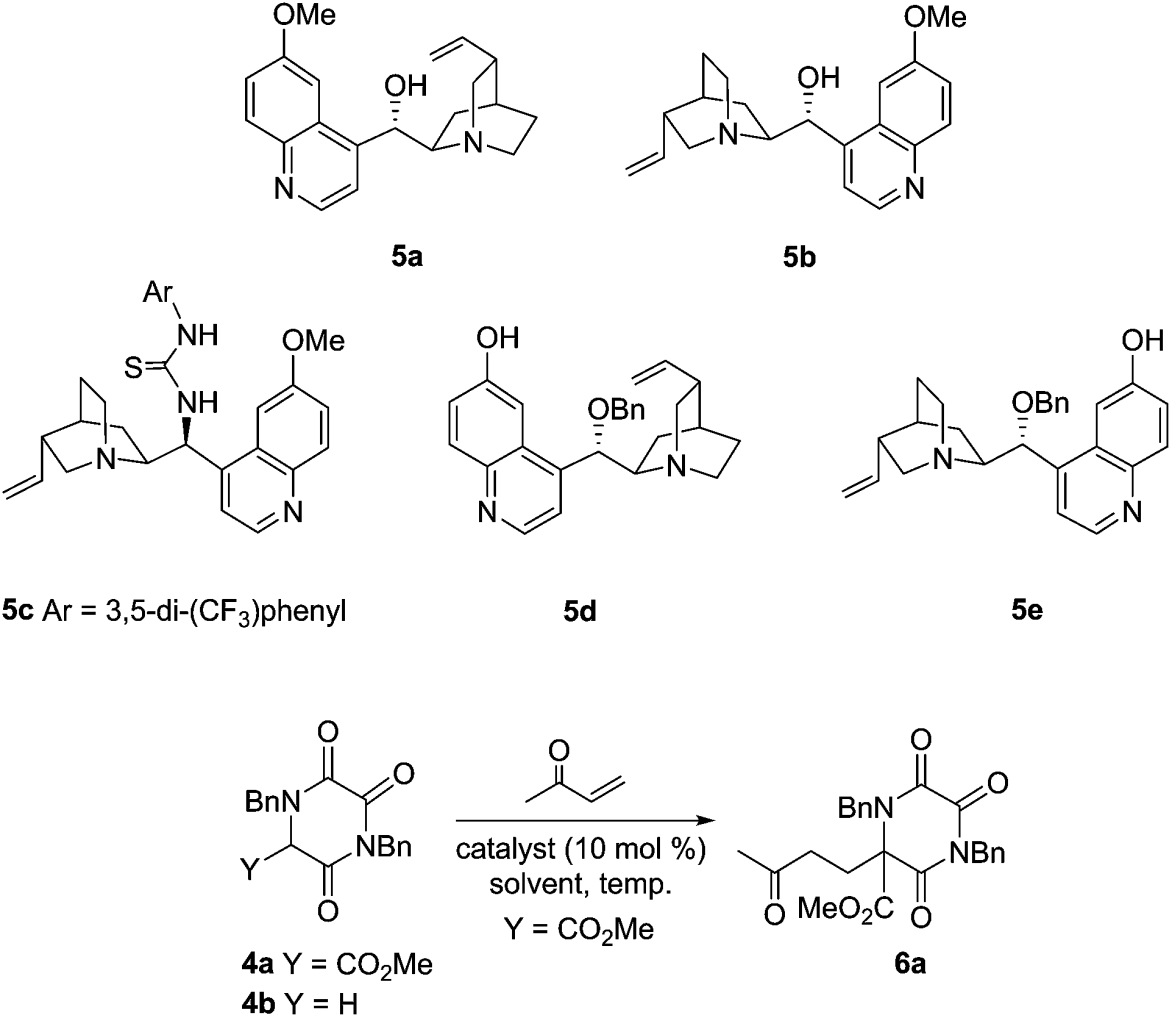						
Entry	Catalyst	Temp	Solvent	Time^*a*^	**6a** ^*b*^ [%]	er^*c*^
1	**5a**	RT	CH_2_Cl_2_	20	94	82 : 18
2	**5b**	RT	CH_2_Cl_2_	20	91	25 : 75
3	**5c**	RT	CH_2_Cl_2_	20	98	31 : 69
4	**5d**	RT	CH_2_Cl_2_	20	99	96 : 4
5	**5d**	RT	THF	20	98	95 : 5
6	**5d**	RT	Toluene	20	98	94 : 6
7	**5d**	RT	MeCN	20	99	94 : 6
8	**5d**	0 °C	CH_2_Cl_2_	60	99	98 : 2
9	**5d**	0 °C	CH_2_Cl_2_	120	91^*d*^	97 : 3
10	**5e**	0 °C	CH_2_Cl_2_	60	82	2 : 98

^*a*^Minutes.

^*b*^Isolated yield after chromatography.

^*c*^Determined by HPLC analysis.

^*d*^Reaction conducted using 1 mol% catalyst.

All of the catalysts explored gave good levels of conversion to the desired Michael adduct **6a** within only 20 minutes at room temperature, with isolated yields being very high. Natural alkaloids quinidine (**5a**) and quinine (**5b**) gave interesting levels of enantioselection, with the modified thiourea system (**5c**) being slightly less selective.^11^ The catalyst systems **5d** and **5e**, developed by the Deng group, having a free quinoline phenol and an alkylated C-9 secondary alcohol, immediately displayed exciting levels of selectivity, especially in CH_2_Cl_2_ at 0 °C.^12^ As anticipated, pseudoenantiomeric catalysts **5d** and **5e** gave opposite enantiomers of the product **6a**, in this case with very high selectivity. The use of only 1 mol% of catalyst **5d** was still adequate to effect smooth conversion to product **6a** without erosion of enantioselectivity (entry 9).^13^

When these partly optimised conditions were explored with alternative Michael acceptors, and then with the parent TKP **4b**, we found some slight further tailoring of the conditions was required. In particular, TKP **4b** showed a tendency towards double Michael addition, which could be controlled simply by reducing the reaction temperature to –20 °C. The use of this lower temperature in turn extended reaction times, particularly with β-substituted Michael acceptors, and required us to maintain catalyst loading at 10 mol%. Table 2 shows the results obtained with TKPs **4a** and **4b** with a range of acceptors, including MVK, acrolein and various enones.

**Table 2:** Michael additions of TKPs **4** using catalyst **5d**

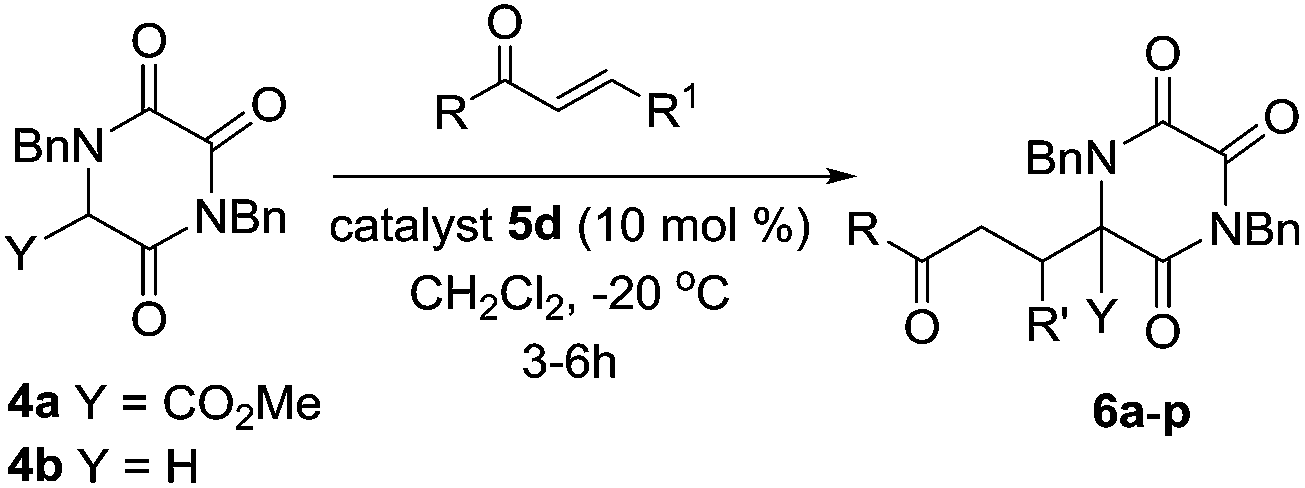					
Entry	TKP	R′	R	**6** ^*a*^ [%]	er^*b*^
1	**4a**	H	Me	**6a** 99	99 : 1
2	**4a**	H	H	**6b** 99	96 : 4^*c*^
3	**4a**	H	Et	**6c** 90	97 : 3
4	**4a**	H	cy-C_6_H_11_	**6d** 99	97 : 3
5	**4a**	H	Ph	**6e** 98	99 : 1
6	**4a**	H	*p*-C_6_H_4_Br	**6f** 98	99 : 1
7	**4a**	H	*p*-C_6_H_4_OMe	**6g** 87	99 : 1
8	**4b**	H	Me	**6h** 80	93 : 7
9	**4b**	H	Et	**6i** 86	96 : 4
10	**4b**	H	cy-C_6_H_11_	**6j** 99	90 : 10
11	**4b**	H	*p*-C_6_H_4_OMe	**6k** 93	88 : 12
12	**4b**	Ph	Ph	**6l** 98	99 : 1^*d*^
13	**4b**	Ph	*o*-C_6_H_4_Br	**6m** 91	88 : 12^*d*^

^*a*^Isolated yield after chromatography.

^*b*^Determined by HPLC analysis.

^*c*^HPLC carried out on derived compound **8**. See Scheme 1.

^*d*^Isolated as a single diastereomer.

Addition reactions with TKP **4a** gave excellent levels of asymmetric induction, with er ≥ 97 : 3 (entries 1–7). The high selectivity observed for both aromatic and aliphatic enones, and for acrolein, is particularly notable. High yields were also obtained in reactions of TKP **4b**, although levels of enantioselection proved more mixed. The lower figures appear to be due to variable erosion of er in the configurationally unstable TKP products rather than inherently lower levels of asymmetric induction. Thus, re-exposure of isolated TKP **6i** to the catalyst **5d**, under the usual conditions, led to erosion of er from 96 : 4 to 89 : 11. This problem should be overcome by more closely monitoring individual reactions, or perhaps by use of more hindered catalysts.

Notwithstanding these issues, the er values for MVK and EVK (entries 8 and 9) are good, and entry 12, involving chalcone as acceptor, demonstrates that excellent selectivities are still possible from the simple TKP **4b**. Notably, both chalcone products **6l** and **6m** were isolated as single diastereoisomers, and no minor isomers could be detected in the ^1^H NMR spectra of the crude materials.

To our knowledge, these are the first enone Michael additions using a simple, non-benzylic, amidic donor,^14^ and are unusual in giving masked α-amino acid products retaining an α-hydrogen.

**Fig. 2:**
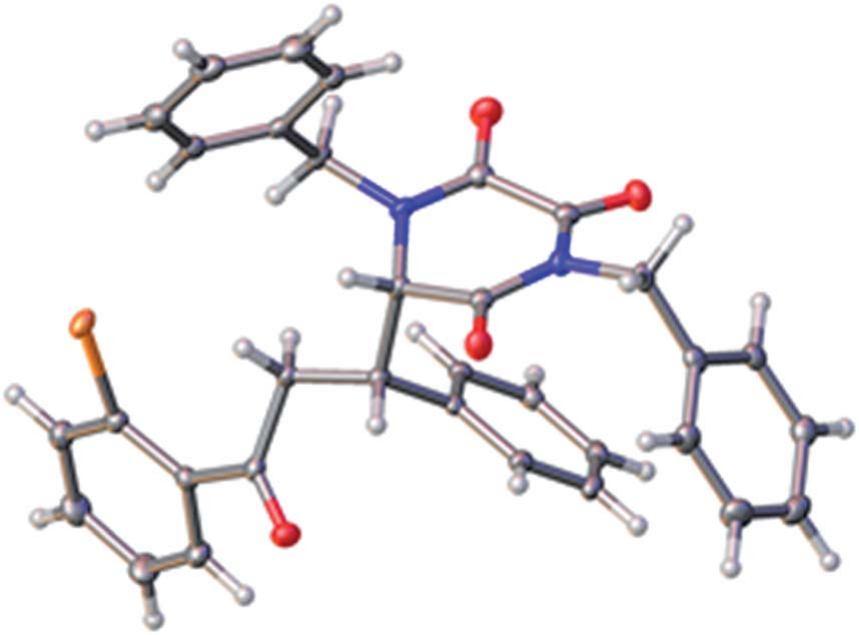
The structure of one of the two crystallographically independent molecules of **6m**, with ellipsoids drawn at the 50% probability level.

Following crystallisation, the absolute and relative configuration of adduct **6m**, generated from reaction of TKP **4b** and *ortho*-bromo chalcone, was determined by X-ray crystallography, Fig. 2.^15^

Interestingly, in the crystal there are two independent molecules of **6m** having different conformations around the newly formed C–C bond (only one is shown, see ESI† for the other one).

From the data available to date, we confidently expect that the absolute configuration of the other products **6** is analogous, *i.e.* (*S*) at the newly formed asymmetric centre at C-6 on the TKP ring.^16^

**Fig. 3:**
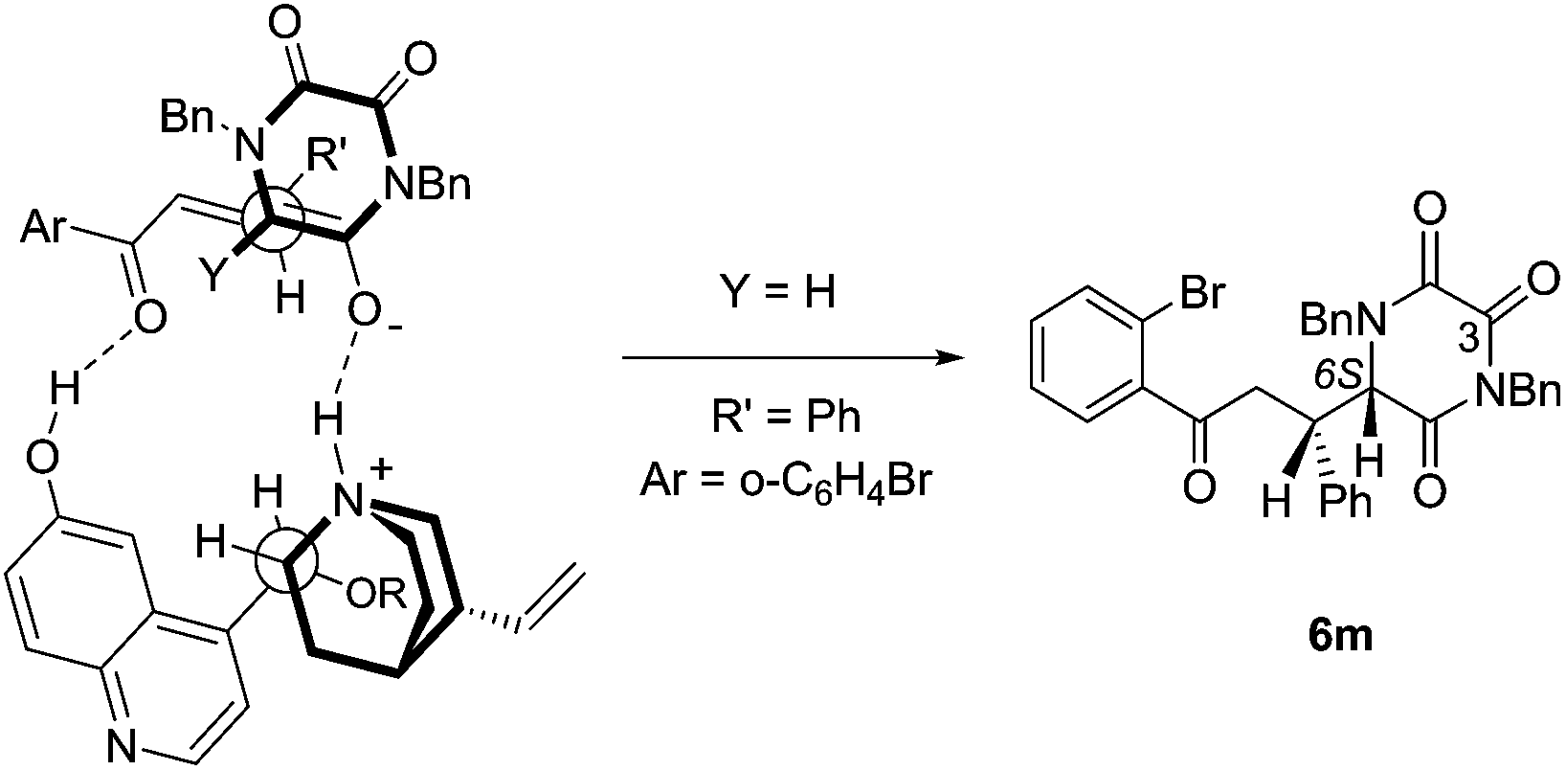
Model for **5d**-catalyzed Michael addition leading to **6m**.

This outcome is in accord with the stereochemical model proposed by Deng, in which the catalyst serves to organize both of the Michael addition partners in an extended array.^12^ This involves activation of the donor by enolate formation through the quinuclidine amine function, and a developing hydrogen-bonded association between the Michael acceptor and the quinoline phenol, Fig. 3.

Application of the Deng model for chalcone systems requires a seemingly counter-intuitive placement of the β-phenyl substituent (R′) *endo* with respect to the TKP ring (albeit a planar, all-sp^2^ ring). That this arrangement is plausible is supported by the finding that one of the crystal structure conformations of **6m** (Fig. 1) exhibits precisely the local conformation about the newly formed C–C bond, as shown in Fig. 2. This arrangement apparently tolerates substitution of the α-hydrogen, Y = H, with an α-CO_2_Me function, this possibly further organising the transition state by an additional hydrogen bond to the quinoline phenol.12*e*

**Scheme 1:**
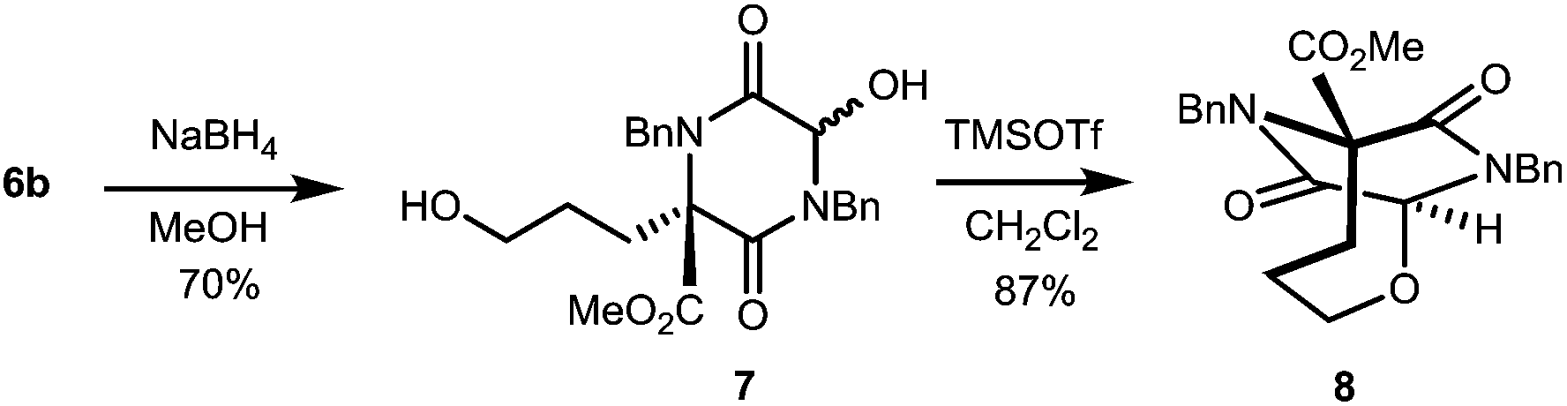
Conversion of Michael adduct **6b** into bridged DKP **8**.

The TKP ring in these chiral products is expected to be electrophilic at C-3, this carbonyl function being both part of an imide, and part of an α-dicarbonyl.^9^ Thus, we anticipate that varied manipulations of these compounds will be possible to give DKPs of various kinds, and by way of preliminary demonstration of this potential, we have transformed **6b** into the ether bridged DKP **8**, *via* diol **7**, Scheme 1.

The use of NaBH_4_ enabled reduction of the aldehyde in adduct **6b** with concomitant regioselective TKP reduction, to give **7**, which then underwent *N*-acyliminium cyclisation using TMSOTf to give **8**.^17^ This sequence was used to convert **6b** into an adduct more suitable for er determination, and the product **8** proved to be highly enantiomerically enriched (>96 : 4 er). This compound has a distinctive 4-atom ether-bridged DKP reminiscent of that present in the antibiotic bicyclomycin (**2**).^2^

**Scheme 2:**
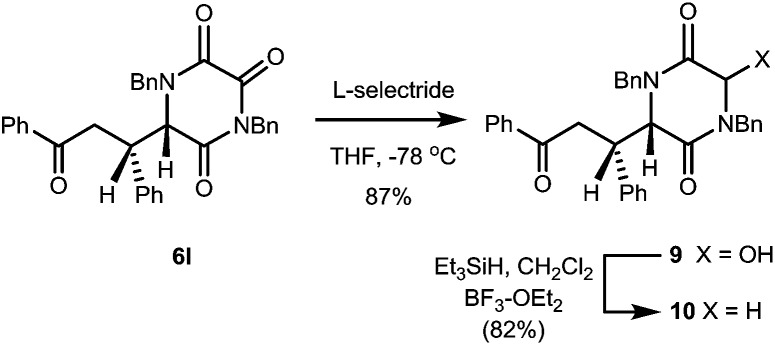
Regioselective reduction of TKP **6l** to give DKP **10**.

We also established that reduction of TKPs to the corresponding DKPs could be achieved in very high overall yield by a two-step process involving initial reduction by l-selectride, then treatment with Et_3_SiH BF_3_–OEt_2_, Scheme 2.

The initial reduction proved highly regioselective for the C-3 TKP C=O function, even in the presence of a side-chain ketone. The hydroxy DKP **9** is an obvious precursor to an *N*-acyliminium type of intermediate, with many possibilities for further transformation.

In this case reduction under standard conditions gave DKP **10**, the er of which (99 : 1) was found to be essentially the same as the starting TKP. This result is particularly significant for manipulation of these systems, since it demonstrates the possibility of converting a configurationally labile TKP into a less acidic, and so more configurationally stable, DKP product.

We subsequently extended our Michael addition studies to include other types of acceptor, including vinyl sulfone, acrylonitrile and acrylate esters. Reactions conducted using TKPs **4a** and **4b** with these partners provided bicyclic hydroxy DKP products **11** directly, *via* a novel Michael–ring-closure process,^18^Table 3.

**Table 3:** Enantioselective Michael–ring-closure of TKPs **4**

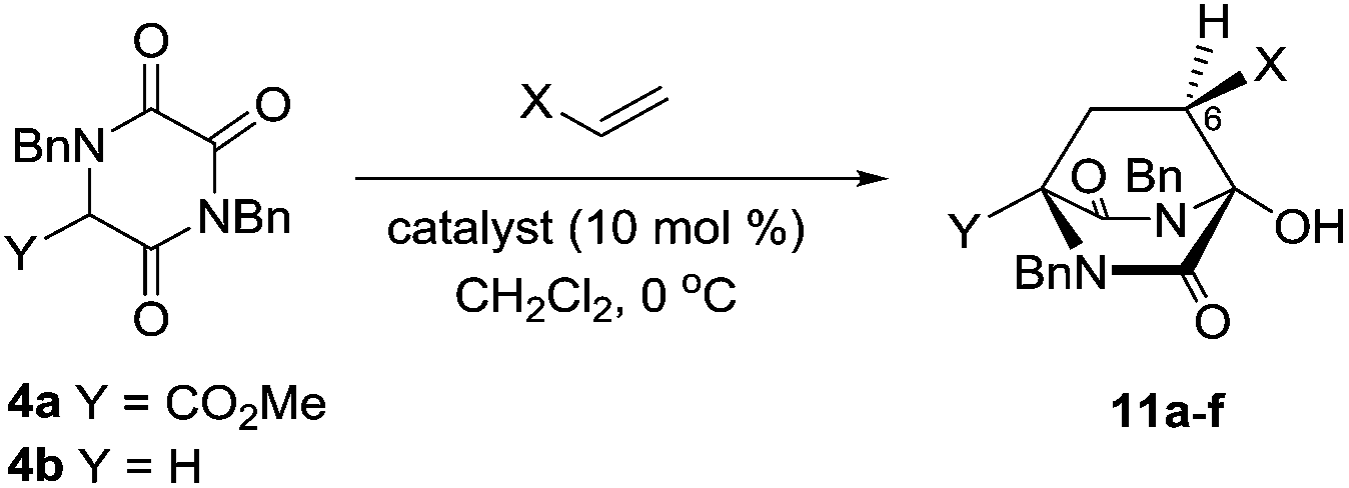					
Entry	TKP	Cat.	X	**11** ^*a*^ [%]	er^*b*^
1	**4a**	**5d**	CO_2_Me	**11a** 98	87 : 13
2	**4a**	**5d**	CN	**11b** 99	95 : 5
3	**4a**	**5d**	SO_2_Ph	**11c** 97	97 : 3
4	**4b**	**5d**	CO_2_Me	**11d** 89	83 : 17
5	**4b**	**5d**	CN	**11e** 98	91 : 9
6	**4b**	**5d**	SO_2_Ph	**11f** 82	93 : 7
7	**4a**	**5c**	CN	**11b** 88	7 : 93^*c*^
8	**4b**	**5c**	CN	**11e** 82	1 : 99^*c*^

^*a*^Isolated yield of single diastereomer after chromatography.

^*b*^Determined by HPLC analysis.

^*c*^Enantiomeric structure to that shown.

The greater nucleophilicity of the intermediate anions or enolates involved in these reactions, compared to those involving enones, is presumably the origin of the observed aldol-like ring-closure. Remarkably, this process delivers bridged DKP products **11** as single diastereomers, with the creation of three new stereogenic centres, and with extraordinary efficiency and very good selectivity, especially in the cases involving vinyl sulfone.^19^ In the case of the nitriles **11b** and **11e** it was found that the thiourea catalyst **5c** also gives excellent results (entries 7 and 8).

These DKPs possess the bicyclo[2.2.2]diazaoctane core structure of stephacidin and related natural products, and the relative configuration at C-6 (stephacidin numbering – see **3**) is also correct for the majority of this family of alkaloids. The potential of this new process to deliver intermediates capable of further elaboration towards bioactive relatives of naturally occurring alkaloids is clear.

## Conclusions

In conclusion, we have demonstrated the utility of the TKP motif in asymmetric cinchona alkaloid-catalyzed Michael additions to access various significant types of chiral DKP with high selectivities. We anticipate that this approach will enable the synthesis of many types of non-racemic TKPs, DKPs and derived structures, particularly those with fully substituted asymmetric centres, and further study of these systems is the subject of ongoing research in our laboratories.

## Supplementary Material

Supplementary information

Crystal structure data
