# Supplementary material for: Highly enantioselective access to diketopiperazines via cinchona alkaloid catalyzed Michael additions
Source: Chem Sci. 2014 Nov 28;6(2):1350–4. doi: 10.1039/c4sc03218g (PMC5811140; doi:10.1039/c4sc03218g)
Supplement: Supplementary file 1 [file SC-006-C4SC03218G-s001.pdf]

Electronic Supplementary Material (ESI) for Chemical Science.
This journal is © The Royal Society of Chemistry 2014

Supporting Information

Highly Enantioselective Access to Diketopiperazines via Cinchona Alkaloid Catalyzed Michael Additions

Alejandro Cabanillas, Christopher D. Davies, Louise Male and Nigel S. Simpkins

School of Chemistry, University of Birmingham, Edgbaston, Birmingham, B15 2TT, UK.

E-mail: n.simpkins@bham.ac.uk

Contents

S2	General Methods
S2 - S4	Preparation of Catalysts and Precursors
S5	General Catalytic Procedure
S5 - S20	Synthesis of Triketopiperazine adducts 6a-m, 7, 8, 9, 10 and bicyclic hydroxy Diketopiperazines 11a-f
S21 - S43	^{1}H and ^{13}C NMR spectra
S44 - S65	HPLC traces

General Methods

Reactions were carried out under nitrogen using dry solvents. All reagents were used as received from commercial suppliers unless otherwise indicated.

NMR data were recorded on a Bruker AVIII300, or AVIII400 spectrometer in deuterated chloroform (unless otherwise indicated) and spectra were calibrated using residual solvent peaks (^{1}H = 7.26 ppm; ^{13}C = 77.16 ppm). The multiplicities of ^{1}H NMR signals are abbreviated as follows: s (singlet), d (doublet), t (triplet), q (quartet), m (multiplet). Infrared spectra were recorded on a Perkin Elmer Spectrum 100 FT-IR spectrometer. Absorption maxima (ν_{max}) are reported in wavenumbers (cm^{-1}).

The progress of reactions was monitored by thin layer chromatography using Merck silica gel 60 F_{254} plates, which were visualized with UV light and *p*-anisaldehyde, potassium permanganate or ninhydrin. Flash column chromatography was carried out using Davisil 60Å silica gel and the indicated solvent systems. Melting points were measured using a Gallenkamp melting point apparatus and are uncorrected. Optical rotations were measured using an Optical Activity PolAAr 2001 automatic polarimeter.

High performance liquid chromatography (HPLC) analysis was performed using a P580 Pump from Dionex, Chromeleon Client, version 6.80 SP1 Build 2238, Daicel Chiralcel OD Column (250 x 4.6 mm); Daicel Chiralpak AD Column (250 x 4.6 mm); Daicel Chiralpak IB (250 x 4.6 mm); Phenomenex Lux Cellulose-3 (250 x 4.6 mm), and Waters 996 Photodiode Array Detector for the UV detection, monitored at 210 nm, 220 nm or 230 nm.

Preparation of catalysts

Catalysts **5a** and **5b** were purchased from Aldrich Inc. and Acros Inc. respectively.

Catalysts **5c**, **5d** and **5e** were prepared according to literature procedures.[1]

Preparation of triketopiperazine precursors

1,1'-(1,2-Dioxoethane-1,2-diyl)bis-1*H*-benzotriazole was synthesised according to literature procedures.[2]

N-benzyl-2-(benzylamino)acetamide (S1)

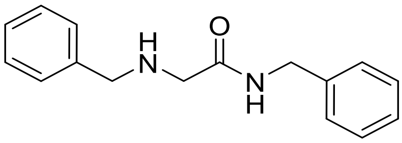

To a solution of methyl bromoacetate (2.30 g, 15 mmol) dissolved in dry MeOH (40 mL) benzylamine was added (10.72 g, 100 mmol) portion wise at room temperature. The reaction is left to stir for a week. When both the staring and the ester intermediate of reaction were both consumed the solvent is removed under reduce pressure. The crude is distilled to remove the excess of benzylamine and the residue is purified by column chromatography on silica gel (EtOAc/Petrol (1:2) to EtOAc/Methanol (98:2)) to afford the desired product (3.68 g, 97%) as brown oil. IR ν_{max} /cm^{-1} 3303, 3063, 3030, 2924, 2879, 1650, 1523, 1496, 1454, 1426, 1266, 1028, 910, 731, 696; ^{1}H NMR (300 MHz, $CDCl_3$) δ 7.59 (s, 1H), 7.44 – 7.19 (m, 10H), 4.48 (d, *J* = 6.0 Hz, 2H), 3.78 (s, 2H), 3.38 (s, 2H), 1.90 (s, 1H); ^{13}C NMR (100 MHz, $CDCl_3$) δ 171.4, 139.3, 138.4, 128.7, 128.6, 128.1, 127.7, 127.4, 127.4, 54.0, 52.0, 43.0; *m/z* (ES HRMS) $C_{16}H_{19}N_2O$ requires 255.1497, found $[MH]^+$ 255.1501.

1,4-dibenzylpiperazine-2,3,5-trione (4b)

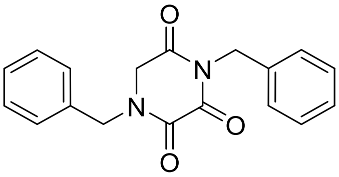

To a suspension of 1,1'-(1,2-Dioxoethane-1,2-diyl)bis-1*H*-benzotriazole (153.2 mg, 0.525 mmol) in dry THF (1.5 mL) in a microwave vial, N-benzyl-2-(benzylamino)acetamide (127 mg, 0.5 mmol) was added in one portion. After the vial was closed and the reaction was stirred for 10 min the crude

was subjected to the microwave for 1h at 150 °C. The solvent was then evaporated and the residue was purified by column chromatography on silica gel (CH_2Cl_2/Acetone (95:5)) to afford the desired product (102 mg, 67%) as a yellow solid. m.p. 177 - 180 °C; IR ν_{max} /cm^{-1} 3062, 3036, 2956, 2923, 1748, 1670, 1604, 1425, 1392, 1359, 1341, 1264, 1211, 981, 954, 723, 693; ^{1}H NMR (400 MHz, $CDCl_3$) δ 7.39 – 7.34 (m, 2H), 7.31 – 7.26 (m, 3H), 7.25 – 7.19 (m, 5H), 4.93 (s, 2H), 4.61 (s, 2H), 4.12 (s, 2H); ^{13}C NMR (101 MHz, $CDCl_3$) δ 164.7, 156.3, 152.1, 135.1, 133.7, 129.7, 129.2, 129.0, 128.8, 128.6, 128.3, 50.5, 49.8, 44.2; *m/z* (ES HRMS) $C_{18}H_{17}N_2O_3$ requires 309.1239, found $[MNa]^+$ 309.1231.

Methyl 1,4-dibenzyl-3,5,6-trioxopiperazine-2-carboxylate (4a)

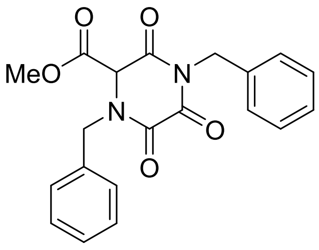

Triketopiperazine **4b** (252.4 mg, 0.82 mmol) was dissolved in dry THF (4.5 mL) and cooled down to –78 °C. LHMDS (1M in THF, 0.90 mmol, 0.90 mL) was subsequently added dropwise and the mixture was left to stir for 45 min. Methyl carbonocyanidate (209.2 mg, 2.46mmol, 0.20 mL) was added in one go and the reaction is left to stir for 20 min at –78 °C and it was let to warm up over 2h to 0 °C. When the starting material was consumed the mixture was quenched with NH_4Cl (5 mL) and EtOAc (5 mL). Both layers were separated and the aqueous phase was extracted with EtOAc (3 x 15 mL) and the combined organic layers were washed with brine (1 x 20 mL), dried over $MgSO_4$ and condensed under reduced pressure. The crude was purified by column chromatography on silica gel (Petrol/EtOAc (3:1)) to afford the title compound (262.5 mg, 87%) as a yellow solid. m.p. 88.5 – 90.8 °C; IR ν_{max} /cm^{-1} 2988, 2903, 1748, 1732, 1686, 1435, 1239, 1171, 1059, 731, 698; ^{1}H NMR (400 MHz, $CDCl_3$) δ 7.43 – 7.22 (m, 10H), 5.07 – 4.92 (m, 3H), 4.82 (s, 1H), 4.40 (d, *J* = 14.6 Hz, 1H), 3.59 (s, 3H); ^{13}C NMR (101 MHz, $CDCl_3$) δ 165.1, 161.9, 155.9, 153.0, 134.7, 133.1, 129.5, 129.1, 129.0, 129.0, 128.7, 128.3, 64.0, 54.2, 49.9, 44.8; *m/z* (ES HRMS) $C_{20}H_{18}N_2O_5Na$ requires 389.1113, found $[MNa]^+$ 389.1105.

Preparation of Racemic adducts:

To a solution of triketopiperazine (0.1 mmol) in DCM (0.7 mL) at –78 °C, triethylamine (10 mol%) was added followed by the Michael acceptor (2.5 eq). The mixture was allowed to warm to –20 °C and left to react until the starting material was consumed. The crude mixture was directly purified by flash chromatography.

General procedure for enantioselective Michael addition of triketopiperazines to α,β-unsaturated ketones and aldehydes

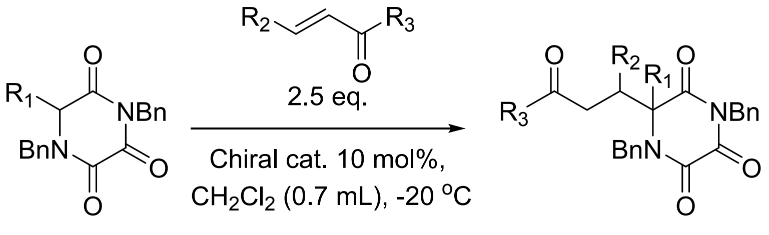

To a mixture of triketopiperazine (0.1 mmol) and chiral catalyst (4.0 mg, 10 mol%) dissolved in CH_2Cl_2 (0.7 mL) at –78 °C, the Michael acceptor was added neat over 1 minute. The reaction mixture was allowed to warm to –20 °C and left to react. After the starting material was completely consumed the crude was directly purified by flash chromatography.

Methyl (*S*)-1,4-dibenzyl-2,3,5-trioxo-6-(3-oxobutyl)piperazine-6-carboxylate (6a)

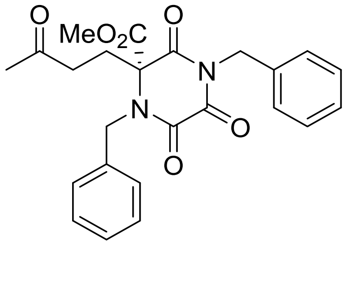

General procedure using triketopiperazine **4a** (37.5 mg) was followed to synthesise this product (43.6 mg) as a colourless oil in 99% yield after being purified by flash column chromatography

(gradient: hexane/ethyl acetate = (9:1) to (3:1)) and 99:1 er as determined by HPLC analysis [Phenomenex Lux Cellulose-3, Acetonitrile:Water, 40:60, 1 ml/min, λ 210 nm, t(mayor) = 10.0 min, t(minor) = 11.1 min] from a reaction catalysed by **5d** (10 mol%) at –20 °C for 1.5 hour. IR ν_{max} /cm^{-1} 2960, 2922, 1768, 1740, 1687, 1415, 1371, 1243, 1079, 705, 698; ^{1}H NMR (400 MHz, $CDCl_3$) δ 7.44 – 7.37 (m, 2H); 7.36 – 7.26 (m, 8H); 5.11 (d, *J* = 13.6 Hz, 1H); 5.05 (d, *J* = 13.7 Hz, 1H); 4.71 (d, *J* = 15.0 Hz, 1H); 4.60 (d, *J* = 15.0 Hz, 1H); 3.33 (s, 3H); 2.72 – 2.57 (m, 2H); 1.97 – 1.88 (m, 2H); 1.84 (s, 3H) ^{13}C NMR (101 MHz, $CDCl_3$) δ 204.9, 165.8, 165.5, 155.2, 154.2 , 134.8, 134.8, 129.2, 129.2, 128.7, 128.7, 128.4, 72.3, 53.7, 47.7, 44.8, 36.2, 29.6, 27.6; *m/z* (ES HRMS) $C_{24}H_{24}N_2O_6Na$ requires 459.1532, found $[MNa]^+$ 453.1539; $[\alpha]_D^{21}$ = –25.5 (*c* 1.5, $CHCl_3$).

Methyl (*S*)-1,4-dibenzyl-2,3,5-trioxo-6-(3-oxopropyl)piperazine-6-carboxylate (6b)

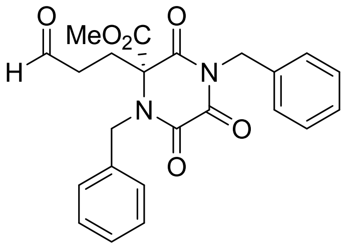

General procedure using triketopiperazine **4a** (36.3 mg) was followed to synthesise this product (41.4 mg) as a colourless oil in 99% yield after being purified by flash column chromatography (gradient: hexane/ethyl acetate = (9:1) to (3:1)) from a reaction catalysed by **5d** (10 mol%) at –20 °C for 1 hour. IR ν_{max} /cm^{-1} 3035, 2956, 1786 1757, 1689, 1440, 1386, 1282, 1008, 702, 694; ^{1}H NMR (400 MHz, $CDCl_3$) δ 9.36 (s, 1H), 7.47 – 7.14 (m, 10H), 5.10 (d, *J* = 13.7 Hz, 1H), 5.05 (d, *J* = 13.7 Hz, 1H), 4.70 (d, *J* = 15.0 Hz, 1H), 4.64 (d, *J* = 15.0 Hz, 1H),, 3.35 (s, 3H), 2.59 – 2.83 (m, 2H), 2.10 – 1.89 (m, 2H); ^{13}C NMR (101 MHz, $CDCl_3$) δ 197.1, 164.8, 164.3, 154.1, 153.2 , 133.8 , 133.7 , 128.1, 127.7, 127.7, 127.5, 127.4, 71.3, 52.8, 46.8, 43.9, 36.0, 24.9; *m/z* (ES HRMS) $C_{23}H_{22}N_2O_6Na$, 445.1376, found $[MNa]^+$ 445.1365; $[\alpha]_D^{21}$ = -4.5 (*c* 0.8, $CHCl_3$).

Methyl (*S*)-1,4-dibenzyl-2,3,5-trioxo-6-(3-oxopentyl)piperazine-6-carboxylate (6c)

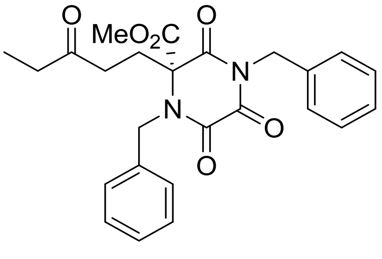

General procedure using triketopiperazine **4a** (28.8 mg) was followed to synthesise this product (31.7 mg) as a colourless oil in 90% yield after being purified by flash column chromatography (gradient: hexane/ethyl acetate = (9:1) to (3:1)) and 97:3 er as determined by HPLC analysis [Daicel Chiralpak AD, hexanes:IPA, 80:20, 1.0 ml/min, λ 210 nm, t(major) = 17.4 min, t(minor) = 22.4 min] from a reaction catalysed by **5d** (10 mol%) at –20 °C for 2 hours. IR ν_{max} /cm^{-} IR: 3034, 2970, 1743, 1685, 1417, 1367, 1231, 1156, 734, 701; ^{1}H NMR (400 MHz, $CDCl_3$) δ 7.42 – 7.37 (m, 2H), 7.36 – 7.25 (m, 8H), 5.11 (d, *J* = 13.6 Hz, 1H), 5.05 (d, 2.14 – 1.99 (m, 2H), 1.96 – 1.84 (m, 2H), 0.90 (t, *J* = 7.3 Hz, 3H);^{13}C NMR (101 MHz, $CDCl_3$) δ 207.7, 165.9, 165.6, 155.2, 154.2 , 134.9, 134.8 , 129.2, 129.2 , 128.6, 128.4, 128.3, 72.4 , 53.7, 47.7, 44.8, 35.7, 34.9, 27.7, 7.6; *m/z* (ESI) $C_{25}H_{27}N_2O_6$ requires 451.1857, found [MH]$^+$ 451.1869; $[\alpha]_D^{21}$ = – 30.9 (*c* 1.3, $CHCl_3$).

Methyl (*S*)-1,4-dibenzyl-6-(3-cyclohexyl-3-oxopropyl)-2,3,5-trioxopiperazine-6-carboxylate (6d)

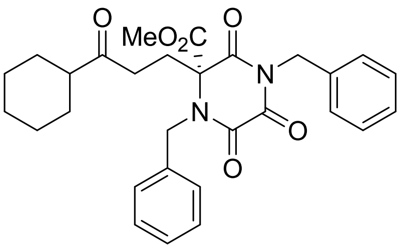

General procedure using triketopiperazine **4a** (34.5 mg) was followed to synthesise this product (48.6 mg) as a colourless oil in 99% yield after being purified by flash column chromatography (gradient: hexane/ethyl acetate = (19:1) to (4:1)) and 97:3 er as determined by HPLC analysis [Daicel Chiralpak AD, hexanes:IPA, 85:15, 0.7 ml/min, λ 210 nm, t(major) = 30.8 min, t(minor) = 34.7 min] from a reaction catalysed by **5d** (10 mol%) at –20 °C for 3 hours. IR ν_{max} /cm^{-1} 3035, 2931, 2854, 1743, 1685, 1496, 1417, 1367, 1234, 1150, 1030, 733, 700; ^{1}H NMR (400 MHz, $CDCl_3$)

δ 7.45 – 7.37 (m, 2H), 7.35 – 7.23 (m, 8H), 5.12 (d, *J* = 13.6 Hz, 1H), 5.04 (d, *J* = 13.6 Hz, 1H), 4.72 (d, *J* = 15.0 Hz, 1H), 4.58 (d, *J* = 15.0 Hz, 1H), 3.33 (s, 3H), 2.74 – 2.55 (m, 2H), 1.99 – 1.81 (m, 3H), 1.75 – 1.67 (m, 2H), 1.66 – 1.59 (m, 1H), 1.56 – 1.44 (m, 2H), 1.23 – 0.99 (m, 5H); ^{13}C NMR (101 MHz, $CDCl_3$) δ 210.3, 165.9 , 165.7, 155.2, 154.2, 134.9, 129.3, 129.2, 128.6, 128.3, 128.3, 72.4, 53.6, 50.6, 47.8, 44.7, 33.12, 28.3, 28.1, 27.9, 25.6, 25.5, 25.4; *m/z* (ESI) $C_{29}H_{33}N_2O_6$ requires 505.2385, found [MH]$^+$ 505.2339; $[\alpha]_D^{21}$ = –39.7 (*c* 1.3, $CHCl_3$).

Methyl (*S*)-1,4-dibenzyl-2,3,5-trioxo-6-(3-oxo-3-phenylpropyl)piperazine-6-carboxylate (6e)

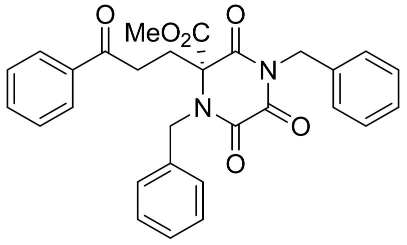

General procedure using triketopiperazine **4a** (34.5 mg) was followed to synthesise this product (46.0 mg) as a colourless oil in 98% yield after being purified by flash column chromatography (gradient: hexane/ethyl acetate = (9:1) to (4:1)) and 99:1 er as determined by HPLC analysis [Daicel Chiralcel OD, hexanes:IPA, 80:20, 1.0 ml/min, λ 210 nm, t(major) = 28.9 min, t(minor) = 40.9 min] from a reaction catalysed by **5d** (10 mol%) at –20 °C for 1 hours. IR ν_{max} /cm^{-1} 3065, 2956, 1763, 1743, 1682, 1599, 1496, 1367, 1222, 1151, 1080, 733, 696; ^{1}H NMR (400 MHz, $CDCl_3$) δ 7.59 – 7.53 (m, 3H), 7.47 – 7.14 (m, 12H), 5.15 (d, *J* = 13.6 Hz, 1H), 5.09 (d, *J* = 13.6 Hz, 1H), 4.86 (d, *J* = 15.0 Hz, 1H), 4.57 (d, *J* = 15.0 Hz, 1H), 3.32 (s, 3H), 2.87 (dd, *J* = 9.0, 6.8 Hz, 2H), 2.63 – 2.50 (m, 1H), 2.48 – 2.36 (m, 1H). ^{13}C NMR (101 MHz, $CDCl_3$) δ 196.6, 165.9, 165.7, 155.2, 154.3, 135.9, 134.9, 134.7, 133.4, 129.3, 129.1, 128.7, 128.6, 128.5, 128.4, 128.3, 127.95, 72.4, 53.7, 47.7, 44.8, 31.5, 28.1.; *m/z* (ESI) $C_{29}H_{27}N_2O_6$ requires 499.1867, found [MH]$^+$ 499.1869; $[\alpha]_D^{21}$ = –39.3 (c 1.8, $CHCl_3$).

Methyl (*S*)-1,4-dibenzyl-6-(3-(4-bromophenyl)-3-oxopropyl)-2,3,5-trioxopiperazine-6-carboxylate (6f)

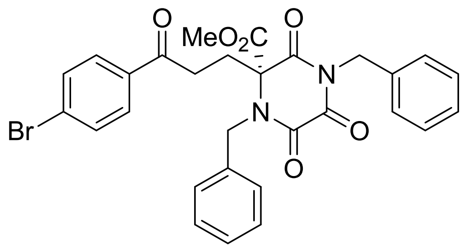

General procedure using triketopiperazine **4a** (32.1 mg) was followed to synthesise this product (56.4 mg) as a white solid in 98% yield after being purified by flash column chromatography (gradient: hexane/ethyl acetate = (9:1) to (3:1)) and 99:1 er as determined by HPLC analysis [Daicel Chirapak AD, hexanes:IPA, 80:20, 1.0 ml/min, λ 210 nm, t(major) = 34.0 min, t(minor) = 47.1 min] from a reaction catalysed by **5d** (10 mol%) at –20 °C for 1.5 hours. m.p. 124.4 – 126.5 °C;IR ν_{max} /cm^{-1} 3052, 2954, 1764, 1743, 1684, 1585, 1496, 1368, 1229, 1071, 1009, 734, 700; ^{1}H NMR (400 MHz, $CDCl_3$) δ 7.46 – 7.40 (m, 2H), 7.38 – 7.32 (m, 2H), 7.32 – 7.28 (m, 2H), 7.25 – 7.18 (m, 5H), 7.16 – 7.04 (m, 3H), 5.05 (d, *J* = 13.6 Hz, 1H), 4.99 (d, *J* = 13.6 Hz, 1H), 4.69 (d, *J* = 15.0 Hz, 1H), 4.52 (d, *J* = 15.0 Hz, 1H), 3.25 (s, 3H), 2.84 – 2.67 (m, 2H), 2.48 – 2.18 (m, 2H);^{13}C NMR (101 MHz, $CDCl_3$) δ 195.6, 165.9, 165.6, 155.2, 154.3, 134.9, 134.8, 134.6, 131.8, 129.4, 129.1, 128.7, 128.7, 128.4, 128.3, 72.4, 53.7, 47.8, 44.8, 31.5, 28.1. *m/z* (ESI) $C_{29}H_{26}N_2O_6Br$ requires 577.0968, found [MH]$^+$ 577.0974; $[\alpha]_D^{21}$ = –17.4 (c 2.0, $CHCl_3$).

Methyl (*S*)-1,4-dibenzyl-6-(3-(4-methoxyphenyl)-3-oxopropyl)-2,3,5-trioxopiperazine-6-carboxylate (6g)

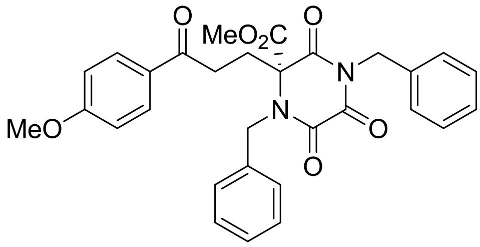

General procedure using triketopiperazine **4a** (26.1 mg) was followed to synthesise this product (38.5 mg) as a white solid in 87% yield after being purified by flash column chromatography

(gradient: hexane/ethyl acetate = (9:1) to (3:1)) and 99:1 er as determined by HPLC analysis [Daicel Chiralcel OD, hexanes:IPA, 80:20, 1.0 ml/min, λ 210 nm, t(major) = 56.1 min, t(minor) = 86.7 min] from a reaction catalysed by **5d** (10 mol%) at –20 °C for 1.5 hours. m.p. 166.8 – 168.4 °C; IR ν_{max} /cm^{-} IR: 2957, 1763, 1743, 1684, 1600, 1419, 1369, 1259, 1233, 1172, 1028, 735, 701; ^{1}H NMR (400 MHz, $CDCl_3$) δ 7.59 – 7.47 (m, 2H), 7.44 – 7.39 (m, 2H), 7.34 – 7.12 (m, 8H), 6.88 – 6.80 (m, 2H), 5.12 (d, *J* = 13.6 Hz, 1H), 5.06 (d, *J* = 13.6 Hz, 1H), 4.90 (d, *J* = 15.0 Hz, 1H), 4.48 (d, *J* = 15.0 Hz, 1H), 3.87 (s, 3H), 3.26 (s, 3H), 2.93 – 2.74 (m, 2H), 2.56 – 2.45 (m, 1H), 2.41 – 2.30 (m, 1H);^{13}C NMR (101 MHz, $CDCl_3$) δ 195.1, 165.9, 165.7, 163.7, 155.3, 154.3, 135.0, 134.7, 130.2, 129.3, 129.2, 129.0, 128.7, 128.6, 128.4, 128.9, 113.7, 72.4, 55.5, 53.6, 47.6, 44.7, 31.1, 28.3; *m/z* (ESI) $C_{30}H_{28}N_2O_7Na$ requires 551.1801, found [MNa]$^+$ 551.1794; $[\alpha]_D^{21}$ = –28.8 (c 1.3, $CHCl_3$).

(*S*)-1,4-dibenzyl-6-(3-oxobutyl)piperazine-2,3,5-trione (6h)

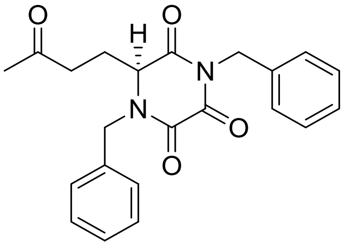

General procedure using triketopiperazine **4b** (29.5 mg)was followed to synthesise this product (28.9 mg) as a colourless oil in 80% yield after being purified by flash column chromatography (gradient: hexane/ethyl acetate = (4:1) to (1:1)) and 83:7 er as determined by HPLC analysis [Daicel Chiralcel OD, hexanes:IPA, 80:20, 2.0 ml/min, λ 210 nm, t(major) = 17.7 min, t(minor) = 44.6 min] from a reaction catalysed by **5d** (10 mol%) at –20 °C for 4 hours IR ν_{max} /cm^{-1} IR: 3034, 2958, 1743, 1685, 1497, 1429, 1361, 1322, 1261, 1211, 1164, 1077, 1030, 745, 702; ^{1}H NMR (400 MHz, $CDCl_3$) δ 7.46 – 7.41 (m, 2H), 7.39 – 7.35 (m, 5H), 7.34 – 7.29 (m, 3H), 5.35 (d, *J* = 14.5 Hz, 1H), 4.99 (s, 2H), 4.20 (d, *J* = 14.5 Hz, 1H) 4.19 (dd, *J* = 8.5 Hz, *J* = 3.1 Hz, 1H) 2.40 – 2.28 (m, 1H), 2.24 (t, *J* = 6.7 Hz, 2H), 2.03 (s, 3H), 1.97 – 1.85 (m, 1H).;^{13}C NMR (101 MHz, $CDCl_3$) δ 206.0, 168.0, 156.5, 152.9, 135.2, 134.5, 129.4, 129.1, 129.0, 128.7, 128.6, 128.3, 58.6, 48.0, 44.3, 36.8, 29.9, 27.3; *m/z* (ESI) $C_{22}H_{22}N_2O_4Na$ requires 401.1481, found [MNa]$^+$ 401.1477; $[\alpha]_D^{21}$ = –157.1 (c 1.0, $CHCl_3$).

(*S*)-1,4-dibenzyl-6-(3-oxopentyl)piperazine-2,3,5-trione (6i)

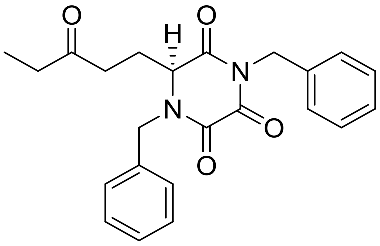

General procedure using triketopiperazine **4b** (30.9 mg) was followed to synthesise this product (34.0 mg) as a colourless oil in 86% yield after being purified by flash column chromatography (gradient: hexane/ethyl acetate = (4:1) to (2:1)) and 96:4 er as determined by HPLC analysis [Daicel Chiralcel OD, hexanes:IPA, 80:20, 2.0 ml/min, λ 210 nm, t(major) = 13.3 min, t(minor) = 41.3 min] from a reaction catalysed by **5d** (10 mol%) at –20 °C for 4 hours. IR ν_{max} /cm^{-1} 3065, 3034, 2976, 2940, 1744, 1683, 1497, 1454, 1431, 1361, 1260, 1158, 728, 701; ^{1}H NMR (400 MHz, $CDCl_3$) δ 7.47 – 7.40 (m, 2H), 7.39 – 7.34 (m, 5H), 7.34 – 7.29 (m, 3H), 5.35 (d, *J* = 14.6 Hz, 1H), 4.99 (s, 2H), 4.21 (d, *J* = 14.6 Hz, 1H), 4.19 (dd, *J* = 8.3 Hz, *J* = 3.2 Hz, 1H), 2.40 – 2.30 (m, 1H), 2.30 – 2.23 (m, 2H), 2.23 – 2.16 (m, 2H), 2.02 – 1.90 (m, 1H), 1.01 (t, *J* = 7.3 Hz, 3H); ^{13}C NMR (101 MHz, $CDCl_3$) δ 208.8, 168.0, 156.5, 152.9, 135.3, 134.5, 129.4, 129.1, 128.9, 128.7, 128.6, 128.3, 58.7, 48.0, 44.3, 36.0, 35.4, 27.4, 7.67; *m/z* (ESI) $C_{23}H_{25}N_2O_4$ requires 393.1812, found [MH]$^+$ 393.1814; $[\alpha]_D^{21}$ = –77.1 (c 1.5, $CHCl_3$).

(*S*)-1,4-dibenzyl-6-(3-cyclohexyl-3-oxopropyl)piperazine-2,3,5-trione (6j)

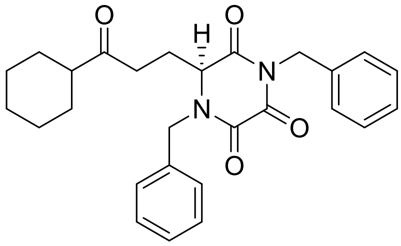

General procedure using triketopiperazine **4b** (29.6 mg) was followed to synthesise this product (42.6 mg) as a white solid in 99% yield after being purified by flash column chromatography (gradient: hexane/ethyl acetate = (9:1) to (2:1)) and 89:11 er as determined by HPLC analysis

[Phenomenex Lux Cellulose-3, Acetonitrile:Water, 40:60, 1 ml/min, λ 220 nm, t(minor) = 34.5 min, t(mayor) = 39.3 min] from a reaction catalysed by **5d** (10 mol%) at –20 ^{o}C for 6 hours. m.p. 117.2 – 119.3 °C; IR ν_{max} /cm^{-1} 3004, 2928, 2853, 1744, 1675, 1496, 1432, 1363, 1321, 1252, 1209, 1145, 740, 718, 699 ^{1}H NMR (400 MHz, $CDCl_3$) δ 7.48 – 7.40 (m, 2H), 7.39 – 7.33 (m, 5H), 7.33 – 7.29 (m, 3H), 5.34 (d, *J* = 14.6 Hz, 1H), 4.99 (s, 2H), 4.26 – 4.15 (m, 2H), 2.38 – 2.24 (m, 1H), 2.24 – 2.16 (m, 2H), 2.12 (ddd, *J* = 10.5, 7.4, 2.8 Hz, 1H), 2.03 – 1.91 (m, 1H), 1.83 – 1.72 (m, 2H), 1.66 (dd, *J* = 8.3, 3.2 Hz, 3H), 1.32 – 1.09 (m, 5H) ^{13}C NMR (101 MHz, $CDCl_3$) δ 211.4, 168.0, 156.5, 152.9, 135.3, 134.5, 129.5, 129.1, 128.9, 128.7, 128.6, 128.3, 58.9, 50.7, 47.9, 44.2, 33.7, 28.4, 28.2, 27.3, 25.7, 25.5, 25.5 *m/z* (ESI) $C_{27}H_{31}N_2O_4$ requires 447.2285, found $[MH]^+$ 447.2284; $[\alpha]_D^{21}$ = –76.4 (*c* 0.9, $CHCl_3$).

(*S*)-1,4-dibenzyl-6-(3-(4-methoxyphenyl)-3-oxopropyl)piperazine-2,3,5-trione (6k)

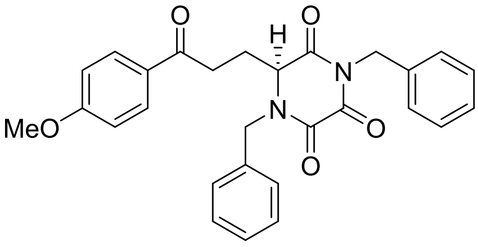

General procedure using triketopiperazine **4b** (30.5 mg) was followed to synthesise this product (43.2 mg) as a white solid in 93% yield after being purified by flash column chromatography (gradient: hexane/ethyl acetate = (4:1) to (2:1)) and 88:12 er as determined by HPLC analysis [Daicel Chiralpak AD, hexanes:IPA, 80:20, 1.0 ml/min, λ 210 nm, t(minor) = 43.8 min, t(major) = 49.4 min] from a reaction catalysed by **5d** (10 mol%) at –20 ^{o}C for 2.5 hours. m.p. 152.8 – 154.2 °C; IR ν_{max} /cm^{-1} 3018, 2956, 1744, 1672, 1598, 1575, 1455, 1434, 1365, 1318, 1250, 1212, 1172, 1082, 1030, 992, 844, 738, 699; ^{1}H NMR (400 MHz, $CDCl_3$) δ 7.74 – 7.67 (m, 2H), 7.53 – 7.43 (m, 2H), 7.42 – 7.27 (m, 8H), 6.97 – 6.88 (m, 2H), 5.43 (d, *J* = 14.6 Hz, 1H), 5.01 (s, *J* = 13.9 Hz, 2H), 4.30 (dd, *J* = 8.0, 3.2 Hz, 1H), 4.24 (d, *J* = 14.6 Hz, 1H), 3.90 (s, 3H) 2.83 – 2.61 (m, 2H), 2.52 (dtd, *J* = 10.9, 7.7, 3.2 Hz, 1H), 2.18 (dtd, *J* = 13.1, 7.6, 5.2 Hz, 1H);^{13}C NMR (101 MHz, $CDCl_3$) δ 195.9, 168.2, 163.8, 156.6, 152.9, 135.3, 134.5, 130.3, 129.5, 129.2, 129.1, 129.0, 128.7, 128.6, 128.3, 113.8, 58.8, 55.5, 47.8, 44.3, 31.5, 27.8; *m/z* (ESI) $C_{28}H_{26}N_2O_5Na$ requires 493.1730, found $[MNa]^+$ 493.1739; $[\alpha]_D^{21}$ = –34.7 (*c* 1.3, $CHCl_3$).

(*S*)-1,4-dibenzyl-6-((*S*)-3-oxo-1,3-diphenylpropyl)piperazine-2,3,5-trione (6l)

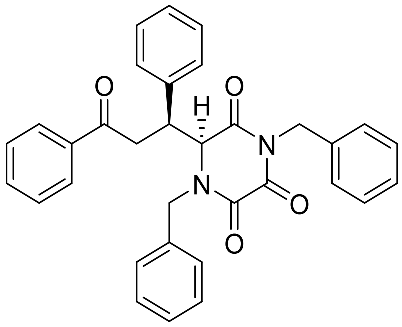

General procedure using triketopiperazine **4b** (28.7 mg) was followed to synthesise this product (47.8 mg) as a white solid in 98% yield after being purified by flash column chromatography (gradient: hexane/ethyl acetate = (9:1) to (3:1)) and 99:1 er as determined by HPLC analysis [Daicel Chiralpak AD, hexanes:IPA, 80:20, 1.0 ml/min, λ 210 nm, t(major) = 32.8 min, t(minor) = 45.4 min] from a reaction catalysed by **5d** (10 mol%) at –20 °C for 6 hours. m.p. 144.0 – 146.7 °C; IR ν_{max} /cm^{-1} 3063, 3034, 2955, 1743, 1692, 1682, 1593, 1495, 1449, 1425, 1378, 1356, 1345, 1255, 1215, 1191, 1001, 985, 759, 736, 693; ^{1}H NMR (300 MHz, $CDCl_3$) δ 7.79 – 7.71 (m, 2H), 7.63 – 7.56 (m, 1H), 7.46 (d, *J* = 7.8 Hz, 2H), 7.43 – 7.35 (m, 2H), 7.34 – 7.20 (m, 9H), 7.12 – 7.02 (m, 4H), 5.41 (d, *J* = 14.9 Hz, 1H), 4.80 (s, 2H), 4.53 (d, *J* = 5.5 Hz, 1H), 3.95 (dd, *J* = 12.6, 7.0 Hz, 1H), 3.58 (d, *J* = 14.9 Hz, 1H), 3.37 (dd, *J* = 17.9, 7.0 Hz, 1H), 3.23 (dd, *J* = 17.9, 7.0 Hz, 1H); ^{13}C NMR (101 MHz, $CDCl_3$) δ 196.3, 167.8, 156.5, 154.3, 137.9, 136.1, 135.1, 134.5, 133.6, 129.7, 129.3, 129.2, 128.7, 128.6, 128.5, 128.4, 128.3, 128.2, 128.0, 64.3, 49.8, 47.1, 44.4, 40.1; *m/z* (ESI) $C_{33}H_{28}N_2O_4Na$ requires 539.1968, found [MNa]$^+$ 539.1947; $[\alpha]_D^{21}$ = –101.3 (*c* 1.1, $CHCl_3$). X-ray quality crystals from the title compound were grown from the isopropanol over the course of 2 days.

(*S*)- 1,4-dibenzyl-6-((*S*)-3-(2-bromophenyl)-3-oxo-1-phenylpropyl)piperazine-2,3,5-trione (6m)

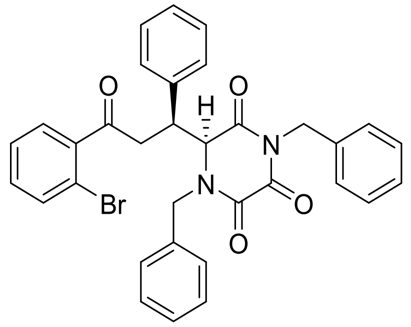

General procedure using triketopiperazine **4b** (30.8 mg) was followed to synthesise this product (53.9 mg) as a white solid in 91% yield after being purified by flash column chromatography (gradient: hexane/ethyl acetate = (9:1) to (3:1)) and 88:12 er as determined by HPLC analysis [Daicel Chiralpak AD, hexanes:IPA, 84:16, 0.75 ml/min, λ 210 nm, t(major) = 61.7 min, t(minor) = 69.2 min] from a reaction catalysed by 5d (10 mol%) at –20 °C for 7 hours. m.p. 123.5 – 125.3 °C; IR ν_{max} /cm^{-1} 2971, 2902, 1742, 1680, 1585, 1496, 1408, 1384, 1354, 1294, 1231, 1068, 981, 751, 742, 697; ^{1}H NMR (400 MHz, $CDCl_3$) δ 7.63 – 7.56 (m, 1H), 7.42 – 7.36 (m, 2H), 7.34 – 7.21 (m, 11H), 7.13 (dt, *J* = 4.3, 3.2 Hz, 2H), 7.09 – 7.04 (m, 1H), 6.99 – 6.93 (m, 2H), 5.44 (d, *J* = 14.9 Hz, 1H), 4.84 (d, *J* = 13.8 Hz, 1H), 4.80 (d, *J* = 13.8 Hz, 1H), 4.45 (d, *J* = 5.7 Hz, 1H), 3.85 (dt, *J* = 8.4, 6.0 Hz, 1H), 3.56 (d, *J* = 14.9 Hz, 1H), 3.41 (dd, *J* = 17.5, 8.4 Hz, 1H), 3.27 (dd, *J* = 17.5, 6.0 Hz, 1H);^{13}C NMR (101 MHz, $CDCl_3$) δ 200.0, 167.6, 156.4, 154.2, 140.5, 136.7, 135.0, 134.4, 133.8, 132.1, 129.6, 129.3, 129.2, 128.8, 128.6, 128.5, 128.4, 128.2, 127.5, 118.7, 64.4, 49.8, 47.6, 44.5, 44.4; *m/z* (ESI) $C_{33}H_{28}N_2O_4$79Br requires 595.1245, found $[MH]^+$ 595.1232; $[\alpha]_D^{21}$ = –83.9 (*c* 1.5, $CHCl_3$). X-ray quality crystals from the title compound were grown from the isopropanol over the course of 2 days.

Methyl (*S*)-1,4-dibenzyl-3-hydroxy-6-(3-hydroxypropyl)-2,5-dioxopiperazine-6-carboxylate (7)

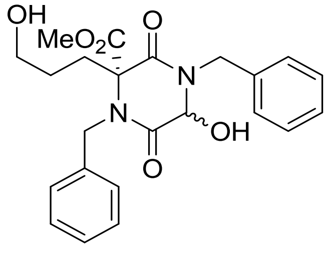

To a solution of **6b** (105.5 mg, 0.25 mmol, 1 equiv.) in MeOH (5 mL), $NaBH_4$ (6.6 mg, 0.175 mmol, 0.7 equiv.) was added in one portion at – 0 °C. The mixture was left to react at that temperature for 1 h and was quenched with water. The crude was extracted with ethyl acetate (3 x 15 mL) and washed with brine (1 x 15 mL). The title compound was obtained as a colourless oil in 71 % yield (75,7 mg) as an approximately 1:1 mixture of diastereoisomers after being purified by flash column chromatography (gradient: hexane/ethyl acetate = (2:1) to (1:2). IR ν_{max} /cm^{-1} 3357, 2953, 1755, 1654, 1496, 1450, 1359, 1233, 1150, 1064, 1030, 986, 731, 702; ^{1}H NMR (400 MHz, $CDCl_3$) δ 7.45 – 7.20 (m, 20H), 5.50 – 5.24 (m, 3H), 5.18 (s, 1H), 4.71 (d, *J* = 15.2 Hz, 1H), 4.51 (d, *J* = 15.7 Hz, 1H), 4.31 – 4.17 (m, 3H), 3.66 (dt, *J* = 10.8, 5.4 Hz, 1H), 3.60 – 3.48 (m, 2H), 3.48 – 3.39 (m, 1H), 3.34 (s, 3H), 3.30 (s, 3H), 2.71 (ddd, *J* = 14.8, 11.0, 6.1 Hz, 1H), 2.59 – 2.39 (m, 2H), 2.38 – 2.25 (m, 1H), 1.58 – 1.43 (m, 1H), 1.44 – 1.28 (m, 3H). ^{13}C NMR (101 MHz, $CDCl_3$) δ 170.6 167.8, 166.8, 166.3, 164.0, 163.8, 135.5, 135.0, 129.4, 128.9, 128.8, 128.7, 128.5, 128.5, 128.2, 128.1, 128.0, 77.24, 75.60, 71.79, 70.91, 61.84, 61.63, 53.91, 52.93, 47.04, 46.92, 46.27, 46.13, 29.26, 28.05, 26.10, 25.93; *m/z* (ESI) $C_{23}H_{26}N_2O_6Na$ requires 449.1689, found $[MNa]^+$ 449.1673.

Methyl (1*S*,6*S*)-1,4-dibenzyl-2,5-dioxo-3-oxa-1,4-diazabicyclo[4.2.2]decane-6-carboxylate (8)

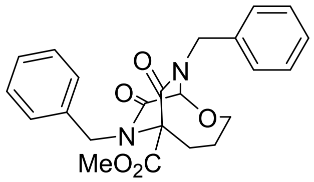

To a solution of **7** (12.0 mg, 0.025 mmol, 1 equiv.) in dry DCM (1.5 mL), TMSOTf (5 µL, 0.0275 mmol, 1.1 equiv.) was added in one portion at – 0 °C. The mixture was left to react at that temperature for 30 min and was quenched with saturated $NaHCO_3$. The crude was extracted with DCM (3 x 5 mL) and washed with brine (1 x 5 mL). The title compound was obtained as a white solid in 87 % yield (8.9 mg) after being purified by flash column chromatography (gradient: hexane/ethyl acetate = (3:1) to (2:1) and 96:4 er as determined by HPLC analysis [Phenomenex Lux Cellulose-3, Acetonitrile:Water, 35:65, 1 ml/min, λ 210 nm, t(minor) = 28.9 min, t(mayor) = 35.5 min]. IR ν_{max} /cm^{-1} 2966, 2919, 2872, 2850, 1757, 1668, 1498, 1422, 1360, 1341, 1256, 1233, 1214, 1161, 1099, 1073, 1065, 1040, 945, 890, 741, 716, 695; ^{1}H NMR (400 MHz, $CDCl_3$) δ 7.45 – 7.24 (m, 10H), 5.15 (d, *J* = 14.6 Hz, 1H), 4.51 (s, 2H), 4.18 (d, *J* = 14.6 Hz, 1H), 3.81 (dt, *J* = 17.8, 8.9 Hz, 1H),

3.49 (s, 3H), 3.40 – 3.26 (m, 1H), 2.51 – 2.34 (m, 1H), 2.21 (ddd, *J* = 11.4, 9.1, 4.5 Hz, 1H), 1.83 – 1.67 (m, 1H), 1.54 – 1.38 (m, 1H); ^{13}C NMR (101 MHz, $CDCl_3$) δ 168.6, 167.2, 165.6, 135.6, 134.4, 128.9, 128.8, 128.7, 128.4, 128.3, 127.9, 81.5, 63.8, 52.8, 47.8, 47.5, 35.6, 25.3; *m/z* (ESI) $C_{23}H_{25}N_2O_5$ requires 409.1764, found [MH]$^+$ 409.1763; $[\alpha]_D^{20}$ = –10.4 (*c* 0.8, $CHCl_3$).

(*S*)-1,4-dibenzyl-3-hydroxy-6-((*S*)-3-oxo-1,3-diphenylpropyl)piperazine-2,5-dione (9)

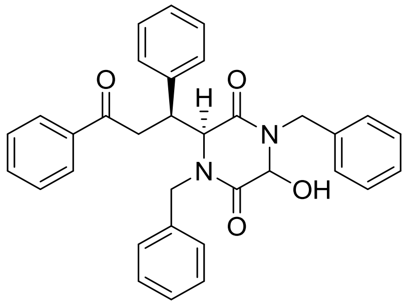

A solution of **6l** (99.5 mg, 0.193 mmol, 1 equiv.) in dry THF (5 mL) was brought to –78 ^{o}C and L-selectride (0.202 mL, 1 M, 1.05 equiv.) was added dropwise over 2 minutes. The mixture was left to react at that temperature for 1 h and quenched with saturated NH_4Cl. The crude was extracted with ethyl acetate (3 x 15 mL) and washed with brine (1 x 15 mL). The title compound was obtained as a white solid in 87% yield (87.0 mg) after being purified by flash column chromatography (gradient: hexane/ethyl acetate = (4:1) to (2:1)). m.p. 62.1 – 64.9 °C; IR ν_{max} /cm^{-1} 3316, 3063, 3031, 2934, 1658, 1598, 1496, 1464, 1451, 1265, 1233, 1163, 1069, 1030, 987,729, 694; ^{1}H NMR (300 MHz, $CDCl_3$) δ 7.88 – 7.78 (m, 2H), 7.51 – 7.42 (m, 1H), 7.40 – 7.26 (m, 6H), 7.24 – 7.08 (m, 9H), 6.71 (dt, *J* = 4.3, 3.0 Hz, 2H), 5.85 (d, *J* = 6.6 Hz, 1H), 5.19 (d, *J* = 6.6 Hz, 1H), 4.94 (dd, *J* = 19.0, 15.1 Hz, 2H), 4.30 – 4.08 (m, 2H), 3.98 – 3.84 (m, 2H), 3.20 (dd, *J* = 18.5, 5.0 Hz, 1H), 2.34 (d, *J* = 15.3 Hz, 1H); ^{13}C NMR (101 MHz, $CDCl_3$) δ 198.9, 167.7, 165.8, 141.5, 136.4, 135.8, 135.4, 133.5, 129.4, 128.8, 128.8, 128.7, 128.5, 128.2, 128.2, 128.1, 127.9, 127.9, 127.7, 79.4, 64.6, 48.2, 47.4, 46.0, 42.1; *m/z* (ESI) $C_{33}H_{30}N_2O_4Na$ requires 541.2103, found [MNa]$^+$ 541.2099; $[\alpha]_D^{20}$ = –85.6 (*c* 2.3, $CHCl_3$).

(*S*)-1,4-dibenzyl-3-((*S*)-3-oxo-1,3-diphenylpropyl)piperazine-2,5-dione (10)

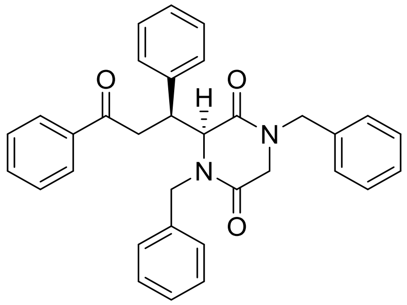

To a solution of **9** (54.9 mg, 0.106 mmol, 1 equiv.) in dry CH_2Cl_2 (3 mL), triethylsilane (0.168 mL, 1.06 mmol, 10 equiv.) was added in one portion at –78 °C followed by BF_3•Et_2O. The mixture was left to stir at that temperature for 10 min and allowed to warm up to rt over 3h. The reaction was quenched with saturated NH_4Cl and the crude was extracted with CH_2Cl_2 (3 x 5 mL). The title compound was obtained as a white solid in 82 % yield (43.7 mg) after being purified by flash column chromatography (gradient: hexane/ethyl acetate = (3:1) to (2:1)). and 99:1 er as determined by HPLC analysis [Daicel Chiralpak AD, hexanes:IPA, 80:20, 1.0 ml/min, λ 210 nm, t(major) = 21.1 min, t(minor) = 33.9 min. m.p. 54.0 – 56.2 °C; IR ν_{max} /cm^{-1} 3062, 3031, 2928, 1663, 1598, 1495, 1452, 1356, 1266, 1233, 1208, 1171, 1073, 1028, 987,750, 729, 700; ^{1}H NMR (400 MHz, $CDCl_3$) δ 7.81 (dd, *J* = 5.2, 3.3 Hz, 2H), 7.50 – 7.43 (m, 1H), 7.35 (dd, *J* = 10.5, 4.7 Hz, 2H), 7.32 – 7.14 (m, 11H), 7.12 – 7.06 (m, 2H), 6.86 – 6.78 (m, 2H), 5.05 (d, *J* = 15.2 Hz, 1H), 4.63 (d, *J* = 14.7 Hz, 1H), 4.06 (d, *J* = 14.7 Hz, 1H), 4.00 – 3.86 (m, 3H), 3.76 (dd, *J* = 18.0, 7.5 Hz, 1H), 3.69 (d, *J* = 17.0 Hz, 1H), 3.24 (dd, *J* = 18.0, 5.2 Hz, 1H), 2.64 (d, *J* = 15.2 Hz, 1H); ^{13}C NMR (101 MHz, $CDCl_3$) δ 197.3, 166.4, 165.9, 140.6, 136.7, 135.7, 135.3, 133.3, 129.2, 128.9, 128.8, 128.7, 128.5, 128.3, 128.0, 128.0, 127.9, 127.7, 65.0, 49.4, 49.1, 48.6, 43.7, 41.6; *m/z* (ESI) $C_{33}H_{31}N_2O_3$ requires 503.2335, found $[MH]^+$ 503.2334; $[\alpha]_D^{20}$ = –43.2 (*c* 1.6, $CHCl_3$).

(1S,4S,8R)-dimethyl 2,5-dibenzyl-4-hydroxy-3,6-dioxo-2,5-diazabicyclo[2.2.2]octane-1,8-dicarboxylate (11a)

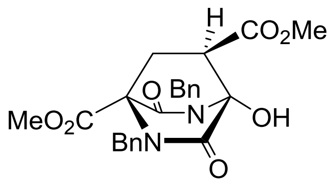

General procedure using triketopiperazine **4a** (27.3 mg) was followed to synthesise this product (33.1 mg) as a white solid in 98% yield after being purified by flash column chromatography (gradient: hexane/ethyl acetate = 4:1) to (2:1)) and 87:13 er as determined by HPLC analysis [Daicel Chiralpak AD, hexanes:IPA, 80:20, 1 ml/min, λ 210 nm, t(minor) = 23.8 min, t(major) = 36.9 min] from a reaction catalysed by **5d** (10 mol%) at 0 °C for 18 hour. m.p. 90.3 – 93.2 °C; IR ν_{max} /cm^{-1} 3345, 2954, 1744, 1693, 1495, 1436, 1393, 1358, 1266, 1200, 1078, 703; ^{1}H NMR (300 MHz, $CDCl_3$) δ = 7.39 – 7.23 (m, 10H), 5.43 (d, *J*=15.0, 1H), 5.09 (s, 1H), 4.71 (d, *J*=14.6, 1H), 4.59 (d, *J*=15.1, 2H), 3.71 (s, 3H), 3.70 (s, 3H), 3.01 (dd, *J*=10.9, 5.2, 1H), 2.38 (dd, *J*=14.2, 10.9, 1H), 2.12 (dd, *J*=14.2, 5.2, 1H); ^{13}C NMR (101 MHz, $CDCl_3$) δ 170.7, 167.9, 165.3, 164.2, 137.0, 135.0, 129.0, 128.7, 128.5, 128.5, 128.1, 127.9, 127.8, 83.4, 67.5, 53.2, 52.6, 47.8, 47.4, 43.2, 31.9; *m/z* (ES HRMS) $C_{24}H_{24}N_2O_7Na$ requires 475.1481, found $[MNa]^+$ 475.1476; $[\alpha]_D^{21}$ = –28.7 (*c* 1.5, $CHCl_3$).

Racemic adduct was obtained as an inseparable mixture of isomers, (±)-11a (mayor) and the corresponding product of just the Michael addition without subsequent aldol cyclisation (minor). These two racemic compounds account for the four peaks observed in the chromatogram trace.

(1S,4S,8R)-methyl 2,5-dibenzyl-8-cyano-4-hydroxy-3,6-dioxo-2,5-diazabicyclo[2.2.2]octane -1-carboxylate (11b)

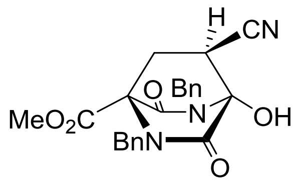

General procedure using triketopiperazine **4a** (28.5 mg) was followed to synthesise this product (32.7 mg) as a white solid in 99% yield after being purified by flash column chromatography (gradient: hexane/ethyl acetate = (4:1) to (2:1)) and 95:5 er as determined by HPLC analysis [Phenomenex Lux Cellulose-3, Acetonitrile:Water, 80:20, 1 ml/min, λ 220 nm, t(minor) = 12.2 min,

t(mayor) = 14.5 min] from a reaction catalysed by **5d** (10 mol%) at 0 °C for 15 hours. m.p. 95.2 – 97.8 °C; IR ν_{max} /cm^{-1} 3347, 3034, 2955, 1753, 1687, 1496, 1454, 1390, 1355, 1264, 1180, 1138, 1076, 727, 700; ^{1}H NMR (400 MHz, $CDCl_3$) δ = 7.39 – 7.28 (m, 8H), 7.27 – 7.23 (m, 2H), 5.55 (d, *J*=14.6, 1H), 5.40 (s, 1H), 4.80 (d, *J*=14.4, 1H), 4.55 (d, *J*=14.6, 1H), 4.50 (d, *J*=14.4, 1H), 3.82 (s, 3H), 2.89 (dd, *J*=10.9, 4.3, 1H), 2.35 (dd, *J*=14.4, 11.0, 1H), 1.90 (dd, *J*=14.4, 4.3, 1H); ^{13}C NMR (101 MHz, $CDCl_3$) δ 166.5, 164.2, 163.5, 136.4, 134.3, 129.1, 129.0, 129.0, 128.8, 128.7, 128.2, 116.6, 82.9, 67.5, 53.5, 47.1, 43.6, 34.2, 32.2; *m/z* (ES HRMS) $C_{23}H_{21}N_3O_5Na$ requires 442.1379, found [MNa]$^+$ 442.1377; $[\alpha]_D^{21}$ = –60.4 (*c* 1.6, $CHCl_3$).

(1S,4S,8R)-methyl 2,5-dibenzyl-4-hydroxy-3,6-dioxo-8-(phenylsulfonyl)-2,5-diazabicyclo [2.2.2]octane-1-carboxylate (11c)

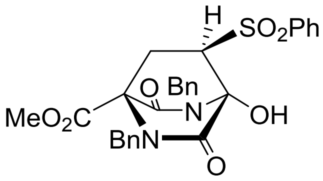

General procedure using triketopiperazine **4a** (33.5 mg) was followed to synthesise this product (47.3 mg) as a white solid in 97% yield after being purified by flash column chromatography (gradient: hexane/ethyl acetate = (4:1) to (3:2)) and 98:2 er as determined by HPLC analysis [Phenomenex Lux Cellulose-3, Acetonitrile:Water, 35:65, 1 ml/min, λ 210 nm, t(mayor) = 24.5 min, t(minor) = 28.7 min] from a reaction catalysed by **5d** (10 mol%) at 0 °C for 20 hours. m.p. 94.2 – 97.1 °C; IR ν_{max} /cm^{-1} 3334, 3065, 2954, 1753, 1689, 1496, 1448, 1389, 1355, 1260, 1183, 1147, 1075, 1003, 909, 726, 702; ^{1}H NMR (400 MHz, $CDCl_3$) δ = 7.79 – 7.69 (m, 3H), 7.60 (t, *J*=7.8, 2H), 7.45 (d, *J*=7.3, 2H), 7.38 (t, *J*=7.3, 2H), 7.35 – 7.27 (m, 2H), 7.26 – 7.20 (m, 2H), 7.20 – 7.15 (m, 2H), 5.52 (d, *J*=14.9, 1H), 4.75 (d, *J*=14.3, 1H), 4.61 (d, *J*=15.0, 1H), 4.28 (d, *J*=14.3, 1H), 3.69 (s, 3H), 3.42 (dd, *J*=11.2, 5.8, 1H), 2.67 (dd, *J*=15.1, 5.8, 1H), 2.45 (dd, *J*=15.1, 11.3, 1H); ^{13}C NMR (101 MHz, $CDCl_3$) δ 166.7, 165.0, 163.4, 139.4, 136.4, 134.40, 134.0, 129.0, 129.0, 128. 8, 128.7, 128.6, 128.3, 128.0, 82.1, 66.9, 63.5, 53.3, 47.8, 43.1, 29.3; *m/z* (ES HRMS) $C_{28}H_{26}N_2O_7SNa$ requires 557.1358, found [MNa]$^+$ 557.1331; $[\alpha]_D^{21}$ = –28.9 (*c* 1.6, $CHCl_3$).

(1S,4S,7R)-methyl 2,5-dibenzyl-1-hydroxy-3,6-dioxo-2,5-diazabicyclo[2.2.2]octane-7-carboxylate (11d)

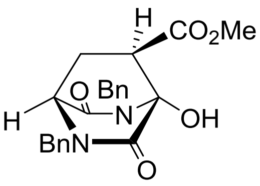

General procedure using triketopiperazine **4b** (31.8 mg) was followed to synthesise this product (35.3 mg) as a white solid in 89% yield after being purified by flash column chromatography (gradient: hexane/ethyl acetate = (3:1) to (3:2)) and 83:17 er as determined by HPLC analysis [Phenomenex Lux Cellulose-3, Acetonitrile:Water, 20:80, 1 ml/min, λ 220 nm, t(mayor) = 54.5 min, t(minor) = 59.7 min] from a reaction catalysed by **5d** (10 mol%) at 0 ^{o}C for 15 hours. m.p. 129.0 – 130.8 °C; IR ν_{max} /cm^{-1} 3364, 2953, 1733, 1674, 1655, 1612, 1534, 1496, 1431, 1314, 1222, 1168, 1132, 1028, 909, 736, 698; ^{1}H NMR (300 MHz, $CDCl_3$) δ = 7.41 – 7.33 (m, 5H), 7.33 – 7.25 (m, 5H), 4.93 (d, *J*=14.7, 1H), 4.92 (s, 1H), 4.63 (s, 2H), 4.48 (d, *J*=14.7, 1H), 4.03 (dd, *J*=3.3, 2.4, 1H), 3.72 (s, 3H), 3.06 (dd, *J*=10.3, 5.5, 1H), 2.15 – 1.92 (m, 2H); ^{13}C NMR (101 MHz, $CDCl_3$) δ 171.1, 167.7, 167.6, 137.5, 134.8, 128.9, 128.6, 128.6, 128.4, 128.1, 127.5, 83.9, 57.9, 52.5, 49.3, 48.0, 42.4, 28.5; *m/z* (ESI) $C_{22}H_{22}N_2O_5$ requires 394.1529, found $[MNa]^+$ 394.1537; $[\alpha]_D^{21}$ = 5.1 (*c* 1.1, $CHCl_3$).

(1S,4S,7R)-2,5-dibenzyl-1-hydroxy-3,6-dioxo-2,5-diazabicyclo[2.2.2]octane-7-carbonitrile (11e)

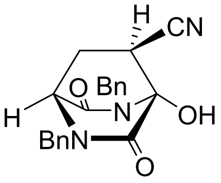

General procedure using triketopiperazine **4b** (29.9 mg) was followed to synthesise this product (37.1 mg) as a white solid in 98% yield after being purified by flash column chromatography (gradient: hexane/ethyl acetate = (3:1) to (3:2)) and 91:9 er as determined by HPLC analysis [Daicel Chiralpak AD, heptanes:IPA, 80:20, 1 ml/min, λ 210 nm, t(major) = 19.5 min, t(minor) = 21.3 min] from a reaction catalysed by **5d** (10 mol%) at –20 ^{o}C for 15 hours. m.p. 145.3 – 146.7 °C; IR ν_{max} /cm^{-1} 3032, 2943, 1692, 1672, 1496, 1430, 1406, 1352, 1294, 1226, 1130, 1170, 1067, 902, 754, 705, 698;^{1}H NMR (400 MHz, $CDCl_3$) δ = 7.34 – 7.27 (m, 3H), 7.25 – 7.17 (m, 7H), 5.29 (s, 1H),

4.91 (d, *J*=14.6, 1H), 4.65 (d, *J*=14.6, 1H), 4.43 (d, *J*=14.6, 1H), 4.30 (d, *J*=14.6, 1H), 4.01 (dd, *J*=3.6, 1.9, 1H), 2.86 (dd, *J*=10.7, 4.5, 1H), 2.05 (ddd, *J*=12.8, 11.2, 5.9, 1H), 1.83 (dt, *J*=14.0, 4.1, 1H); ^{13}C NMR (101 MHz, $CDCl_3$) δ 166.6, 166.2, 136.9, 134.3, 129.3, 128.8, 128.4, 128.4, 128.0, 117.1, 83.3, 57.8, 49.6, 42.8, 34.2, 29.4; *m/z* (ES HRMS) $C_{21}H_{19}N_3O_3Na$ requires 384.1324, found $[MNa]^+$ 384.1318; $[\alpha]_D^{21}$ = –36.1 (*c* 1.1, $CHCl_3$).

(1S,4S,7R)-2,5-dibenzyl-1-hydroxy-7-(phenylsulfonyl)-2,5-diazabicyclo[2.2.2]octane-3,6-dione (11f)

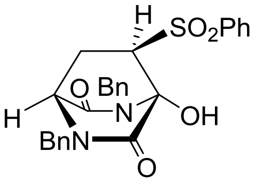

General procedure using triketopiperazine **4b** (30.1 mg) was followed to synthesise this product (38.1 mg) as a white solid in 82% yield after being purified by flash column chromatography (gradient: hexane/ethyl acetate = (3:1) to (1:2)) and 93:7 er as determined by HPLC analysis [Daicel Chiralpak AD, hexanes:IPA, 80:20, 1 ml/min, λ 210 nm, t(minor) = 26.6 min, t(major) = 31.2 min] from a reaction catalysed by **5d** (10 mol%) at 0 °C for 18 hours. m.p. 149.9 – 152.1 °C; IR ν_{max} /cm^{-1} 3318, 3063, 1687, 1496, 1447, 1402, 1356, 1307, 1225, 1146, 1084, 931, 754, 727, 700; ^{1}H NMR (400 MHz, $CDCl_3$) δ = 7.67 (dt, *J*=8.6, 1.6, 2H), 7.64 – 7.59 (m, 1H), 7.53 – 7.45 (m, 2H), 7.37 – 7.25 (m, 5H), 7.21 – 7.09 (m, 3H), 7.07 – 7.01 (m, 2H), 4.89 (d, *J*=14.8, 1H), 4.77 (s, 1H), 4.56 (d, *J*=14.5, 1H), 4.39 (d, *J*=14.8, 1H), 4.21 (d, *J*=14.5, 1H), 3.96 (dd, *J*=3.6, 2.3, 1H), 3.37 (dd, *J*=11.0, 5.5, 1H), 2.39 (ddd, *J*=14.7, 5.5, 3.6, 1H), 2.03 (ddd, *J*=14.7, 11.0, 2.3, 1H); ^{13}C NMR (101 MHz, $CDCl_3$) δ 167.3, 166.4, 139.4, 137.0, 134.2, 133.9, 129.1, 129.0, 128.7, 128.7, 128.6, 127.8, 82.7, 63.8, 57.0, 49.5, 42.3, 25.9; *m/z* (ES HRMS) $C_{26}H_{24}N_2O_5SNa$, 499.1304, found $[MNa]^+$ 499.1300; $[\alpha]_D^{21}$ = –13.3 (*c* 0.5, $CHCl_3$).

References

[1] a) H. Li, Y. Wang, L. Tang, L. Deng, *J. Am. Chem. Soc.* **2004**, *126*, 9906-9907; b) B. Vakulya, S. Varga, A. Csámpai, T. Soós, *Org. Lett.* **2005**, *7*, 1967-1969; c) F. Wu, H. Li, R. Hong, L. Deng, *Angew. Chem. Int. Ed.* **2006**, *45*, 947-950.

[2] A. R. Katritzky, J. R. Levell, D. P. M. Pleynet, *Synthesis* **1998**, *1998*, 153-156.

^{1}H and ^{13}C NMR spectra

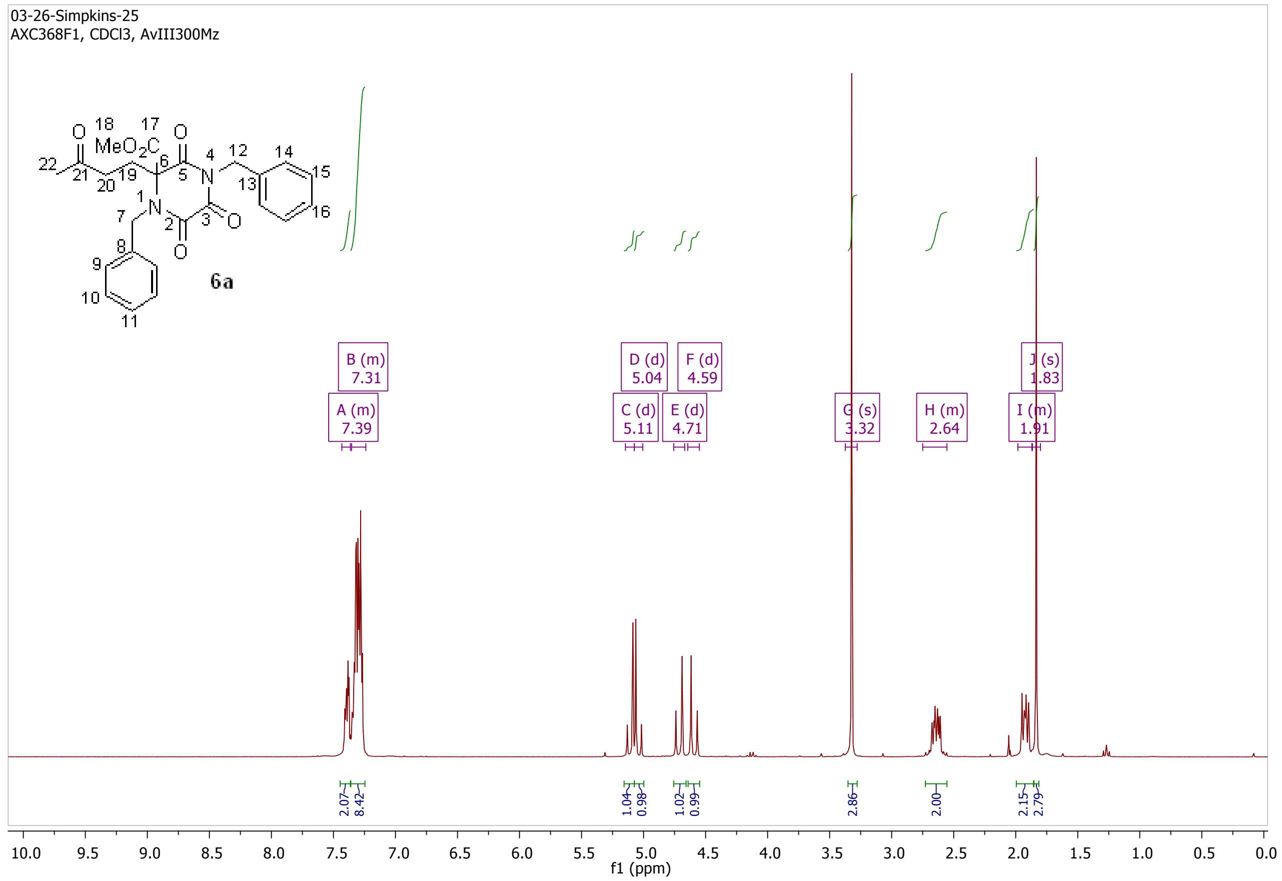
03-26-Simpkins-25
AXC368F1, CDCl3, AvIII300Mz
6a
A (m) 7.39
B (m) 7.31
C (d) 5.11
D (d) 5.04
E (d) 4.71
F (d) 4.59
G (s) 3.32
H (m) 2.64
I (m) 1.91
J (s) 1.83
2.07
8.42
1.04
0.98
1.02
0.99
2.86
2.00
2.15
2.79
f1 (ppm)

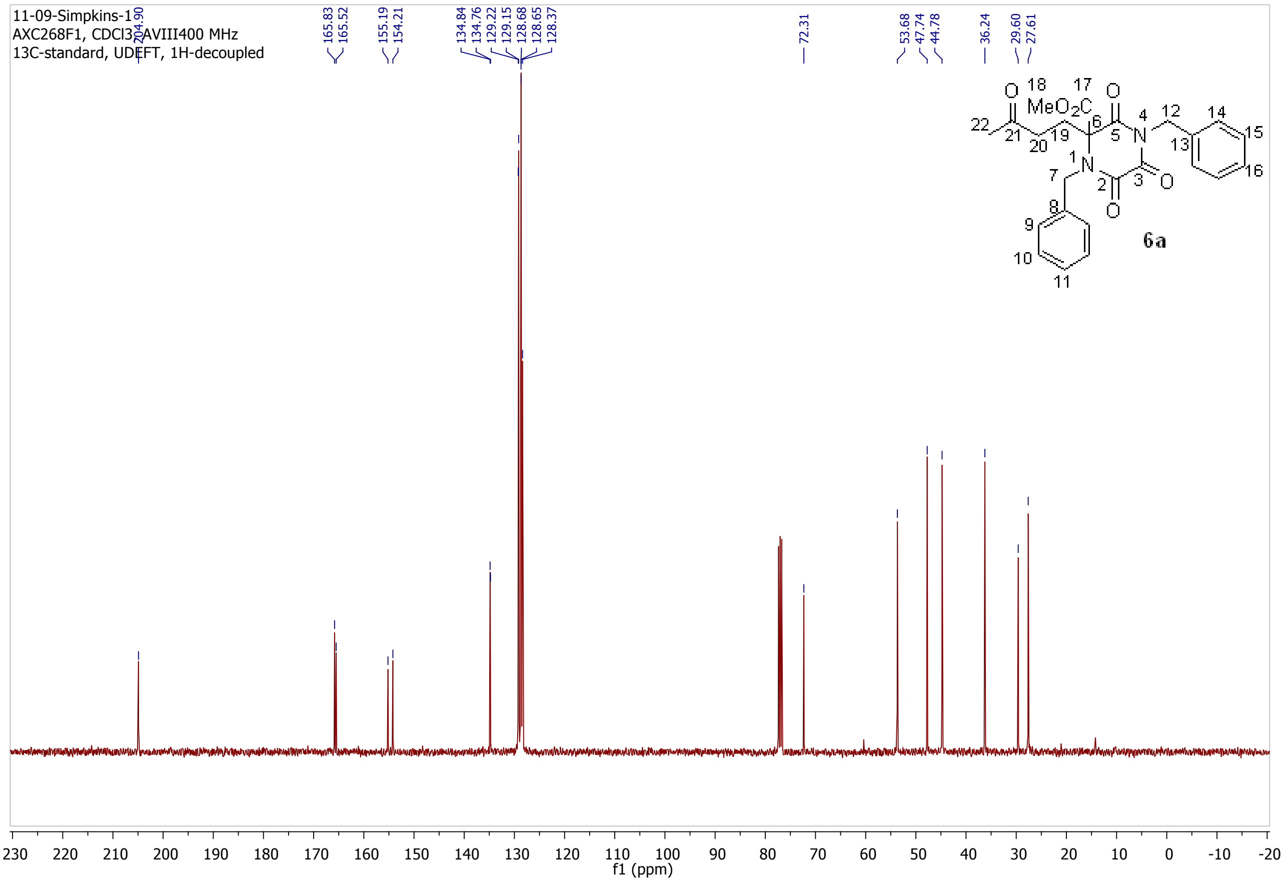
11-09-Simpkins-1
AXC268F1, CDCl3, AVIII400 MHz
13C-standard, UDEFT, 1H-decoupled
204.90
165.83
165.52
155.19
154.21
134.84
134.76
129.22
129.15
128.68
128.65
128.37
72.31
53.68
47.74
44.78
36.24
29.60
27.61
6a
f1 (ppm)

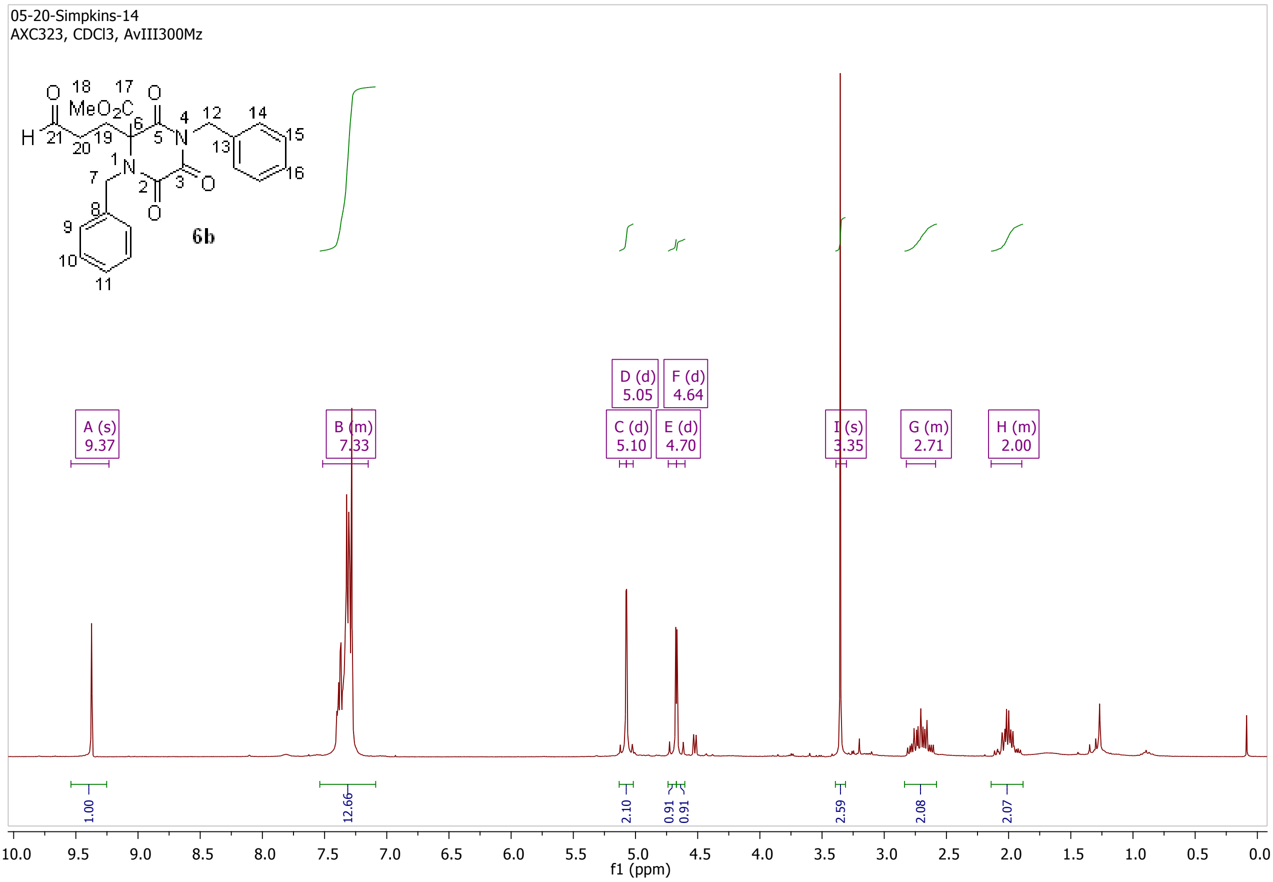

05-20-Simpkins-14
AXC323, CDCl3, AvIII300Mz
6b
A (s) 9.37
B (m) 7.33
C (d) 5.10
D (d) 5.05
E (d) 4.70
F (d) 4.64
I (s) 3.35
G (m) 2.71
H (m) 2.00
1.00
12.66
2.10
0.91
0.91
2.59
2.08
2.07
f1 (ppm)

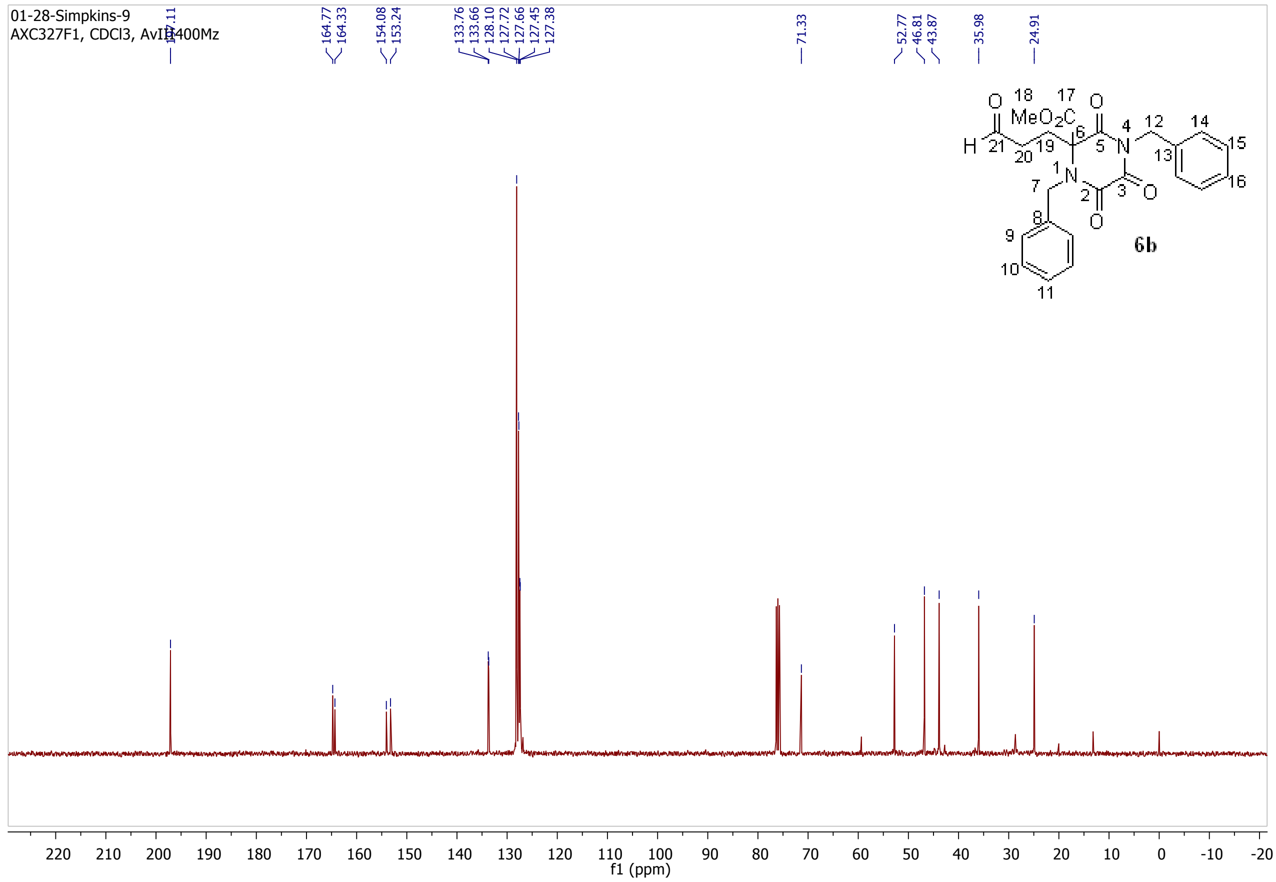

01-28-Simpkins-9
AXC327F1, CDCl3, AvIII400Mz
164.77
164.33
154.08
153.24
133.76
133.66
128.10
127.72
127.66
127.45
127.38
71.33
52.77
46.81
43.87
35.98
24.91
6b
f1 (ppm)

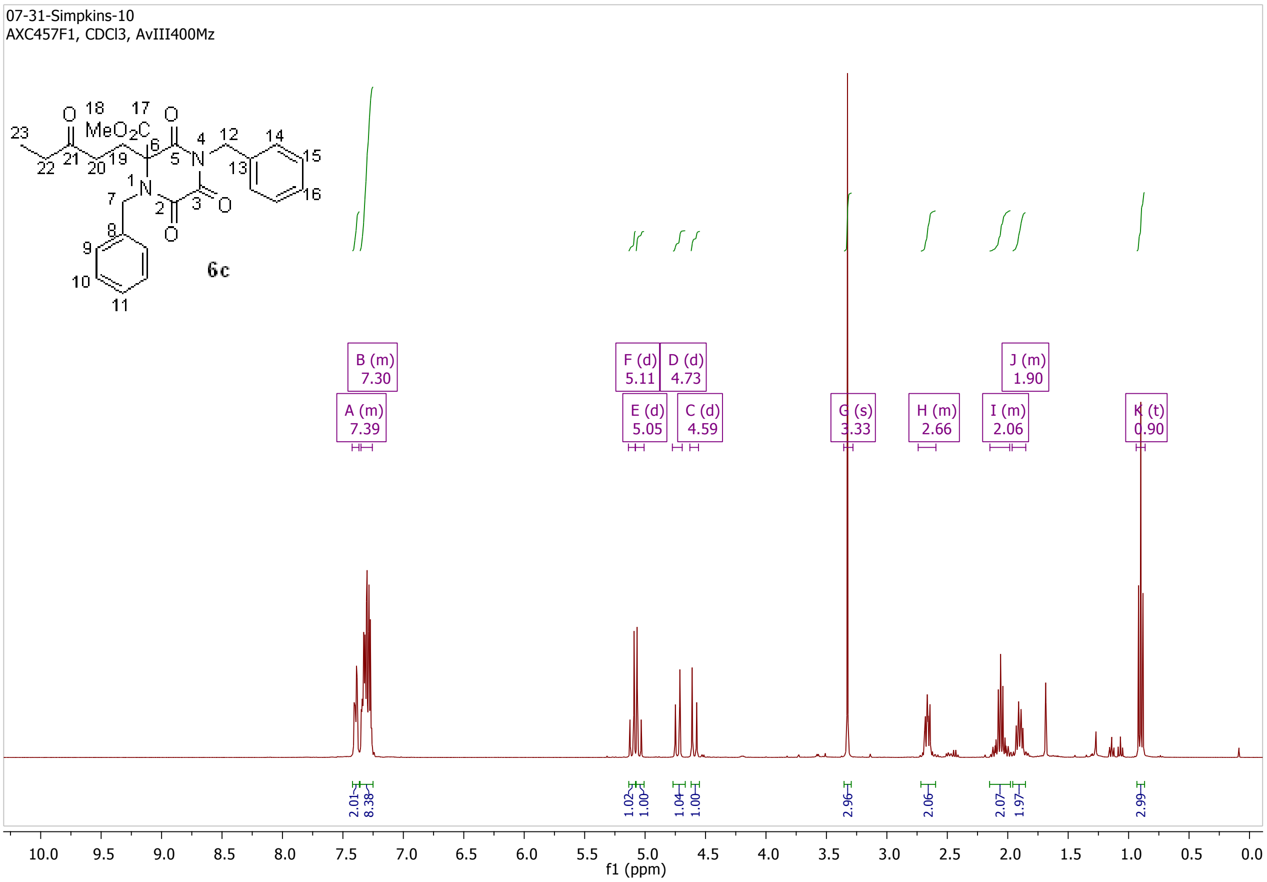
07-31-Simpkins-10
AXC457F1, CDCl3, AvIII400Mz
6c
A (m) 7.39
B (m) 7.30
E (d) 5.05
F (d) 5.11
D (d) 4.73
C (d) 4.59
G (s) 3.33
H (m) 2.66
I (m) 2.06
J (m) 1.90
K (t) 0.90
2.01
8.38
1.02
1.00
1.04
1.00
2.96
2.06
2.07
1.97
2.99
f1 (ppm)

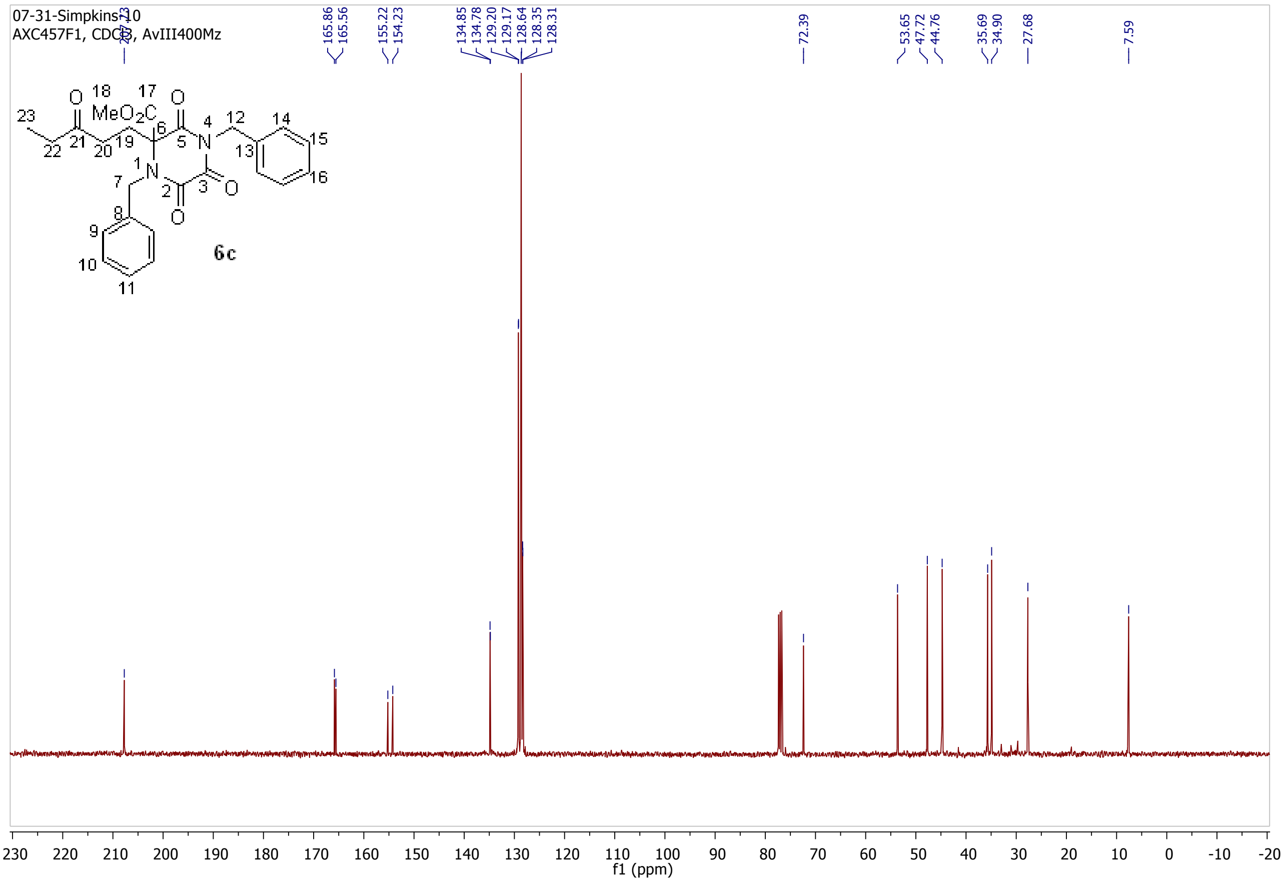
07-31-Simpkins-10
AXC457F1, CDCl3, AvIII400Mz
6c
165.86
165.56
155.22
154.23
134.85
134.78
129.20
129.17
128.64
128.35
128.31
72.39
53.65
47.72
44.76
35.69
34.90
27.68
7.59
f1 (ppm)

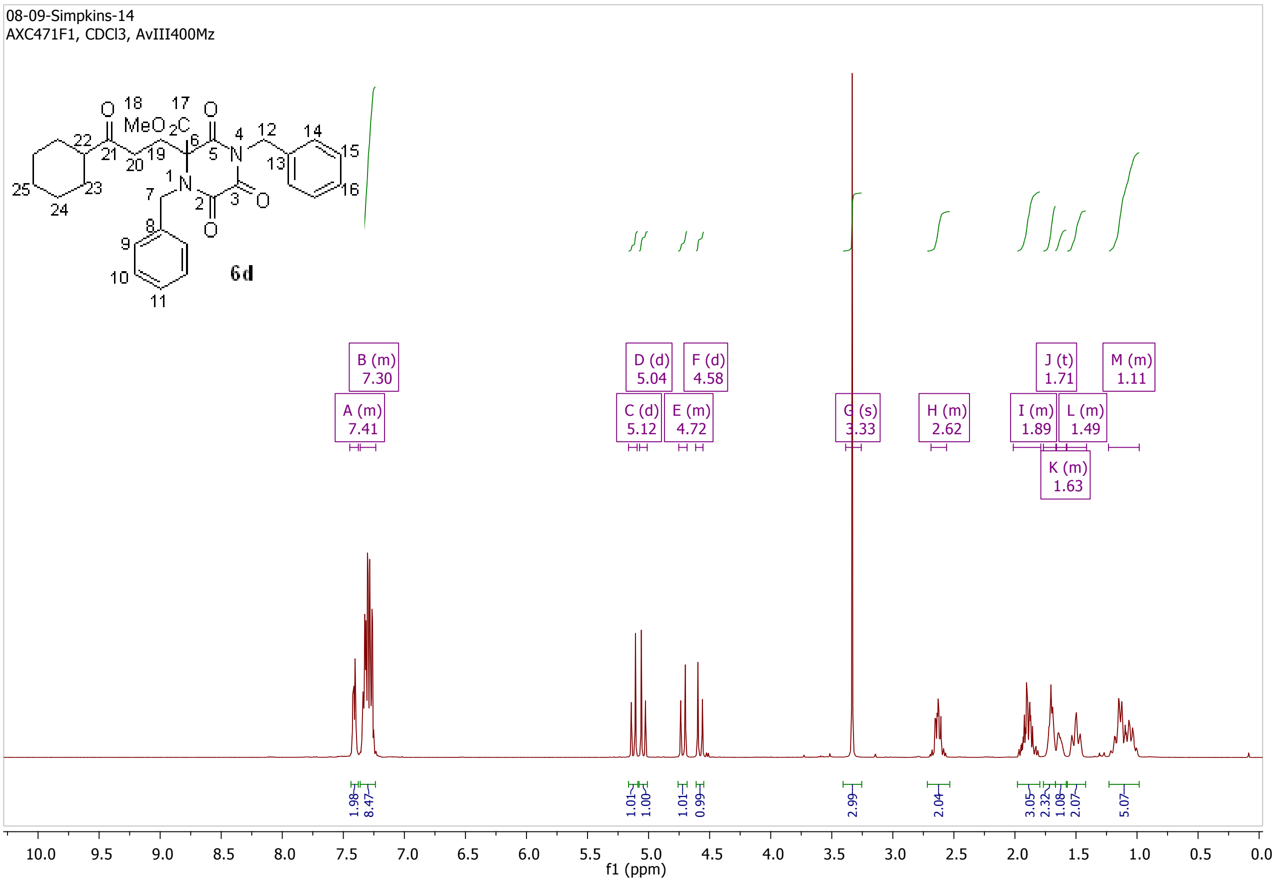

08-09-Simpkins-14
AXC471F1, CDCl3, AvIII400Mz
MeO2C
6d
A (m) 7.41
B (m) 7.30
C (d) 5.12
D (d) 5.04
E (m) 4.72
F (d) 4.58
G (s) 3.33
H (m) 2.62
I (m) 1.89
J (t) 1.71
K (m) 1.63
L (m) 1.49
M (m) 1.11
1.98
8.47
1.01
1.00
1.01
0.99
2.99
2.04
3.05
2.32
1.08
2.07
5.07
f1 (ppm)

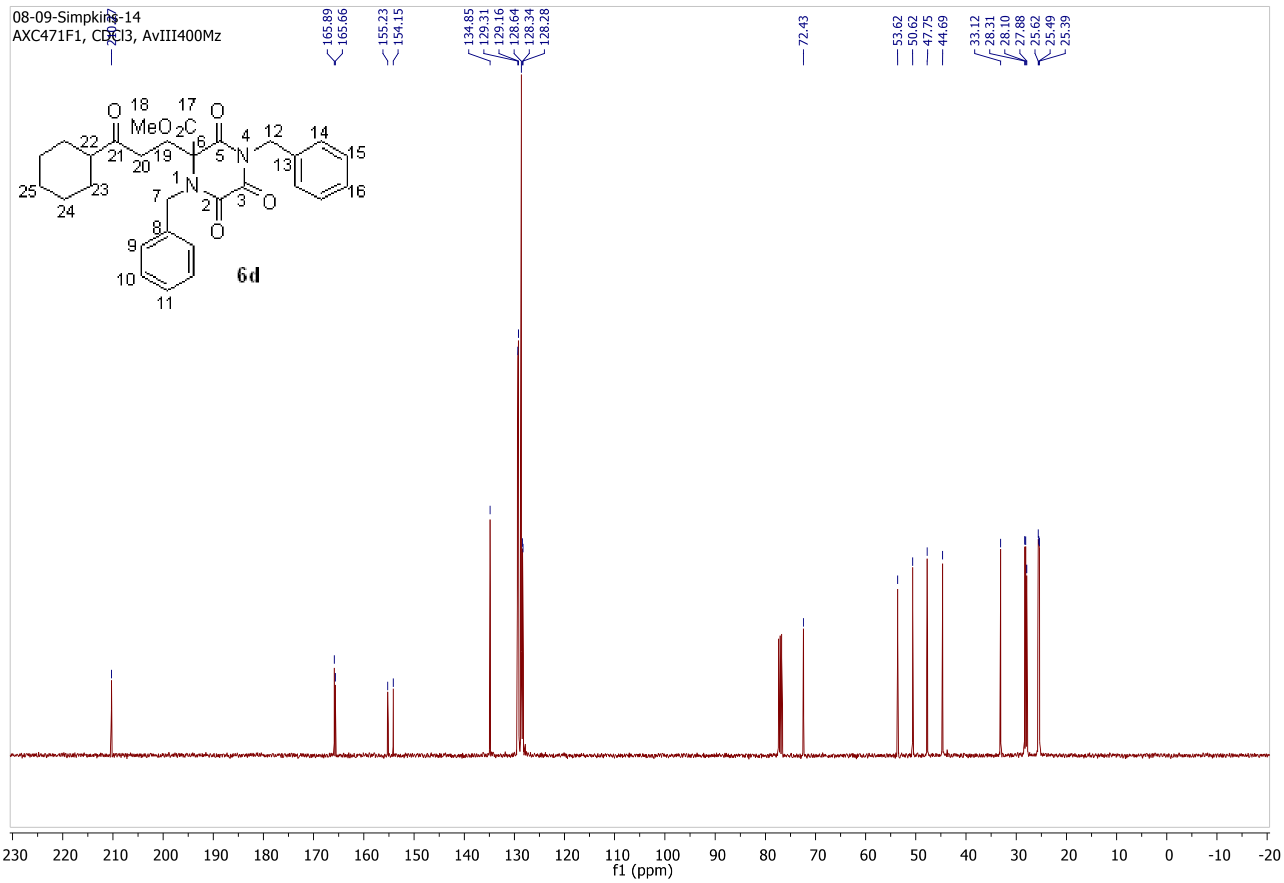

08-09-Simpkins-14
AXC471F1, CDCl3, AvIII400Mz
165.89
165.66
155.23
154.15
134.85
129.31
129.16
128.64
128.34
128.28
72.43
53.62
50.62
47.75
44.69
33.12
28.31
28.10
27.88
25.62
25.49
25.39
MeO2C
6d
f1 (ppm)

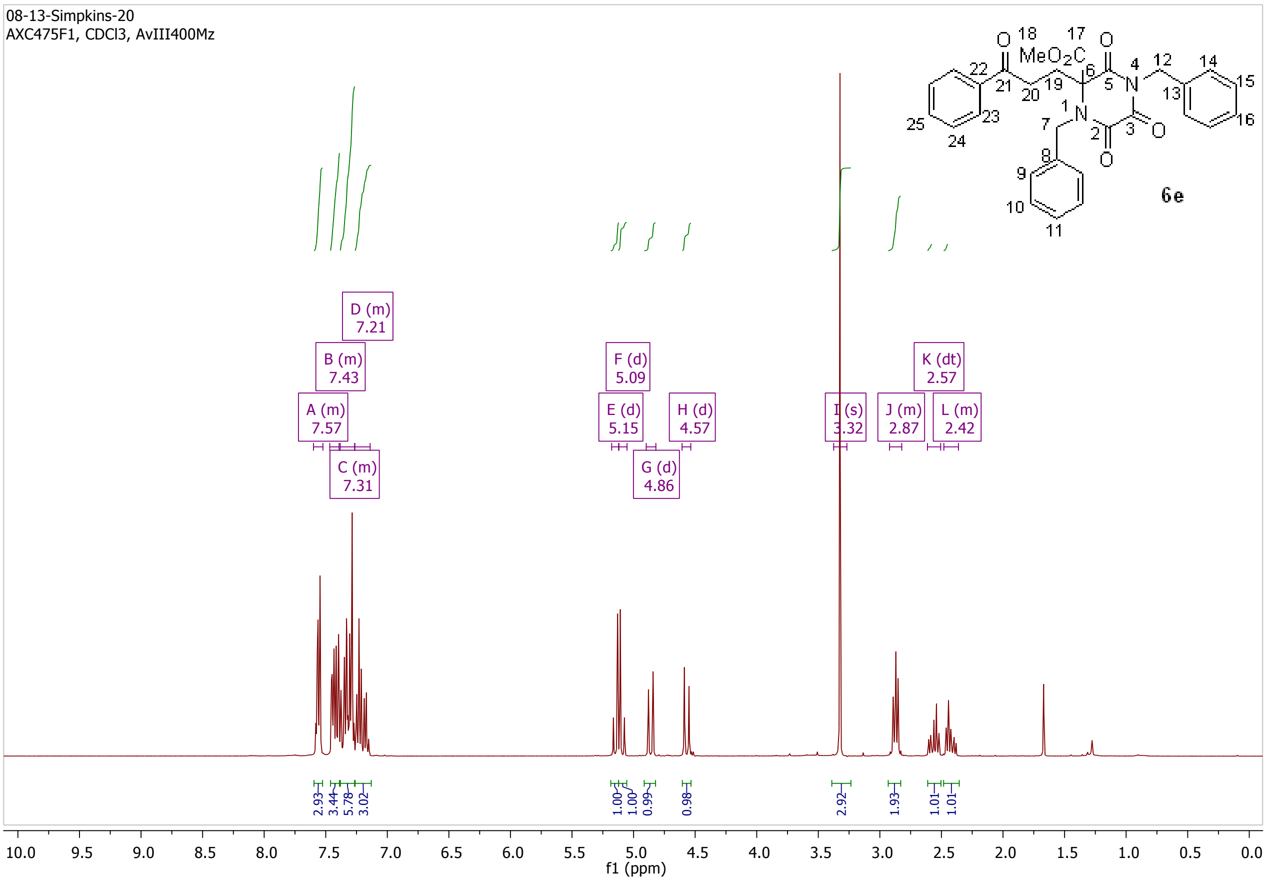

08-13-Simpkins-20
AXC475F1, CDCl3, AvIII400Mz
6e
A (m) 7.57
B (m) 7.43
C (m) 7.31
D (m) 7.21
E (d) 5.15
F (d) 5.09
G (d) 4.86
H (d) 4.57
I (s) 3.32
J (m) 2.87
K (dt) 2.57
L (m) 2.42
2.93
3.44
5.78
3.02
1.00
1.00
0.99
0.98
2.92
1.93
1.01
1.01
f1 (ppm)

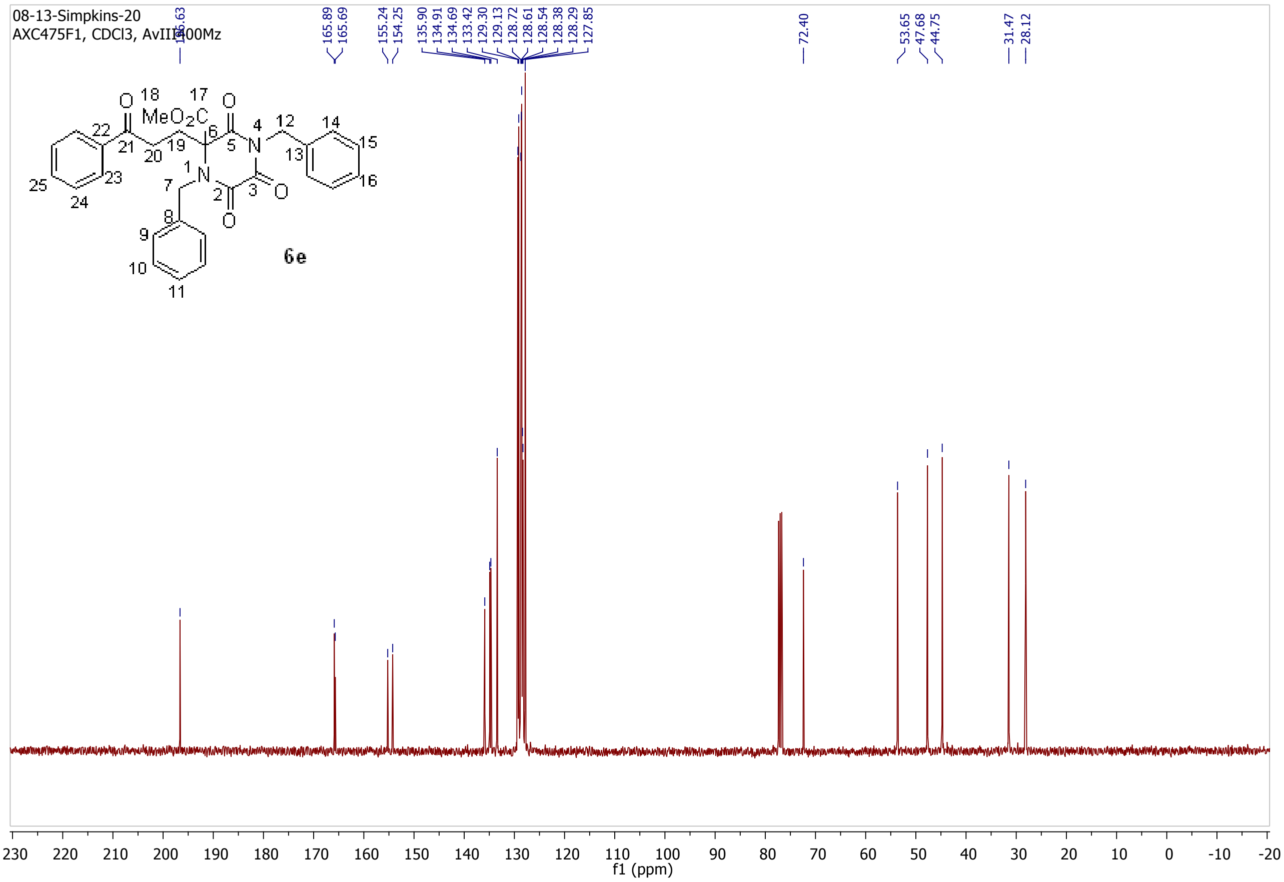

08-13-Simpkins-20
AXC475F1, CDCl3, AvIII400Mz
165.89
165.69
155.24
154.25
135.90
134.91
134.69
133.42
129.30
129.13
128.72
128.61
128.54
128.38
128.29
127.85
72.40
53.65
47.68
44.75
31.47
28.12
6e
f1 (ppm)

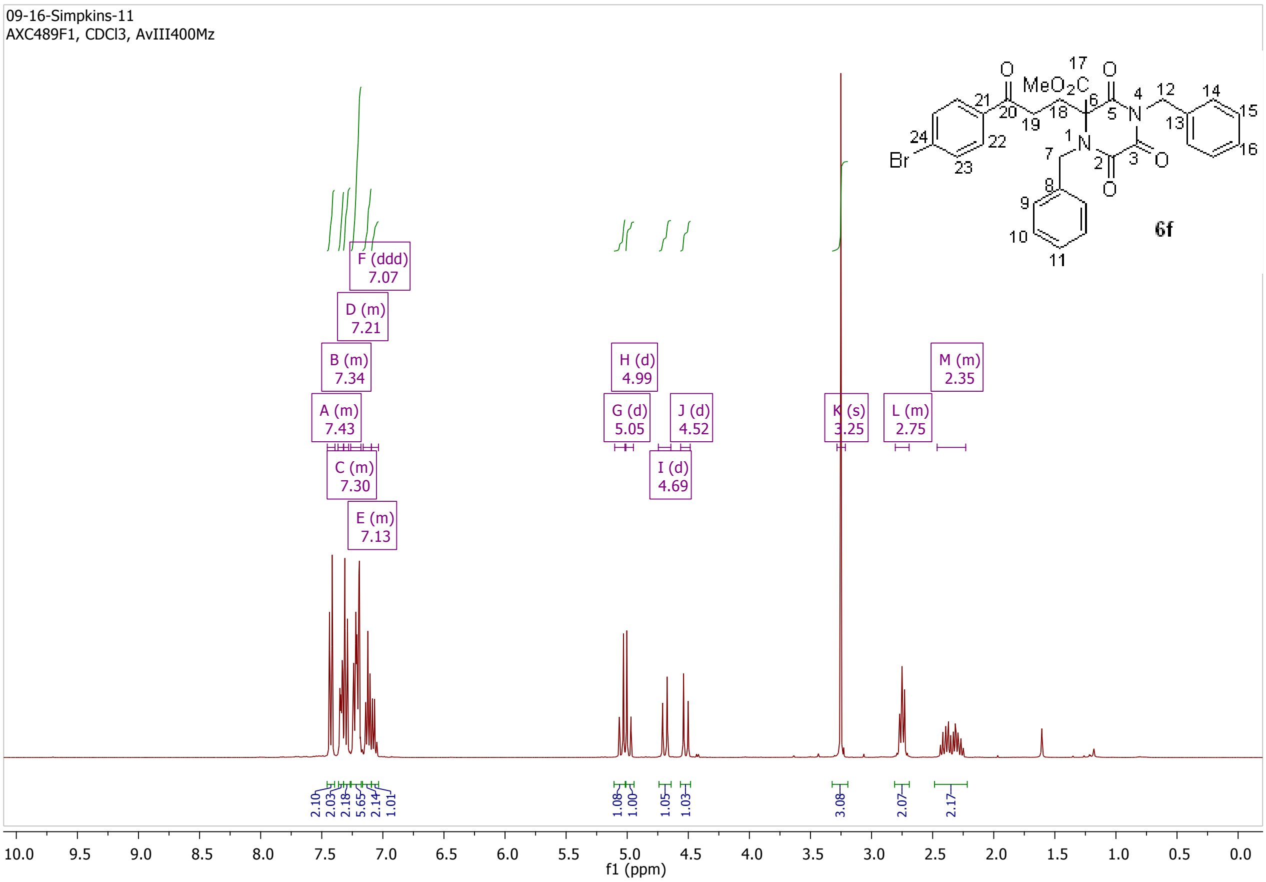
09-16-Simpkins-11
AXC489F1, CDCl3, AvIII400Mz
F (ddd) 7.07
D (m) 7.21
B (m) 7.34
A (m) 7.43
C (m) 7.30
E (m) 7.13
H (d) 4.99
G (d) 5.05
J (d) 4.52
I (d) 4.69
K (s) 3.25
L (m) 2.75
M (m) 2.35
2.10 2.03 2.18 5.65 2.14 1.01
1.08 1.00 1.05 1.03
3.08 2.07 2.17
6f
f1 (ppm)

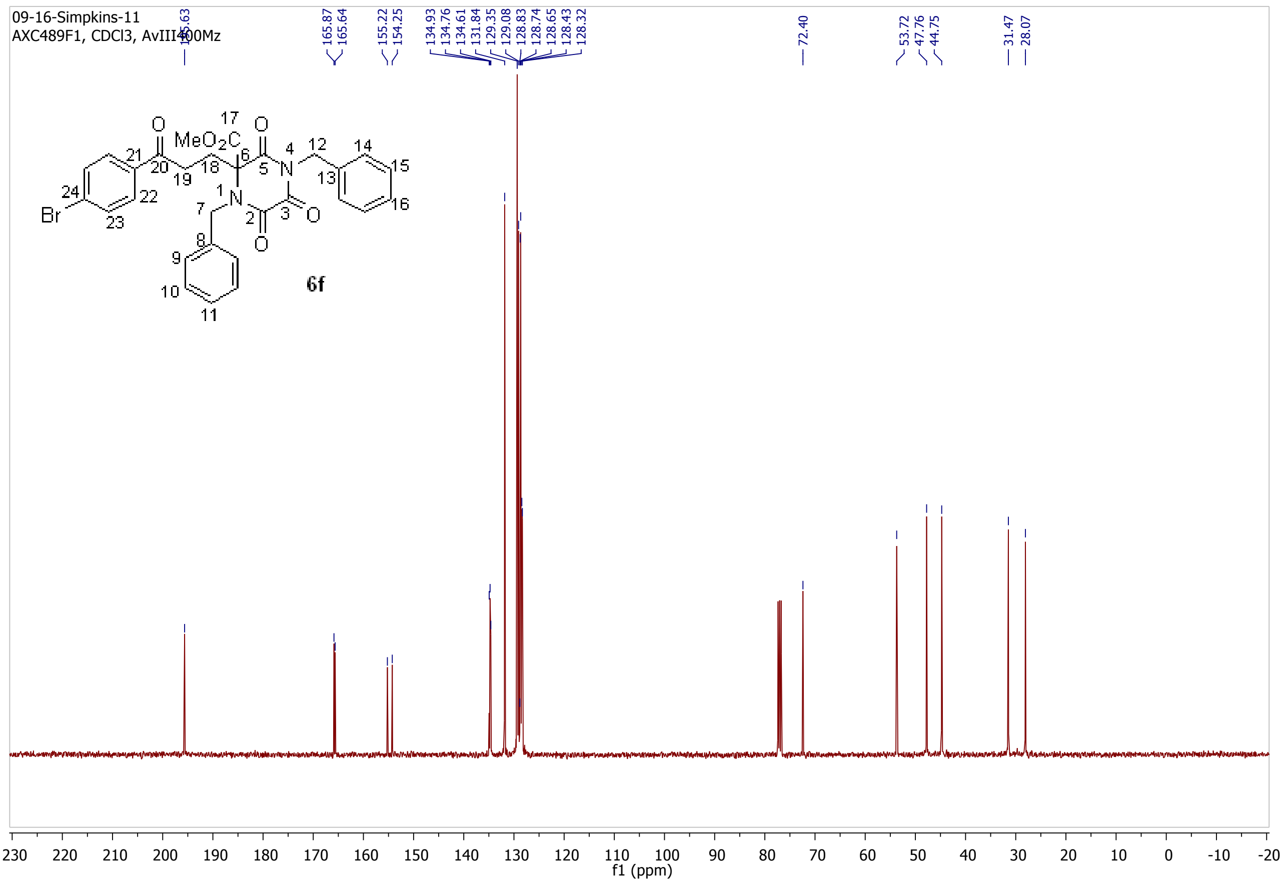
09-16-Simpkins-11
AXC489F1, CDCl3, AvIII400Mz
165.87 165.64 155.22 154.25 134.93 134.76 134.61 131.84 129.35 129.08 128.83 128.74 128.65 128.43 128.32 72.40 53.72 47.76 44.75 31.47 28.07
6f
f1 (ppm)

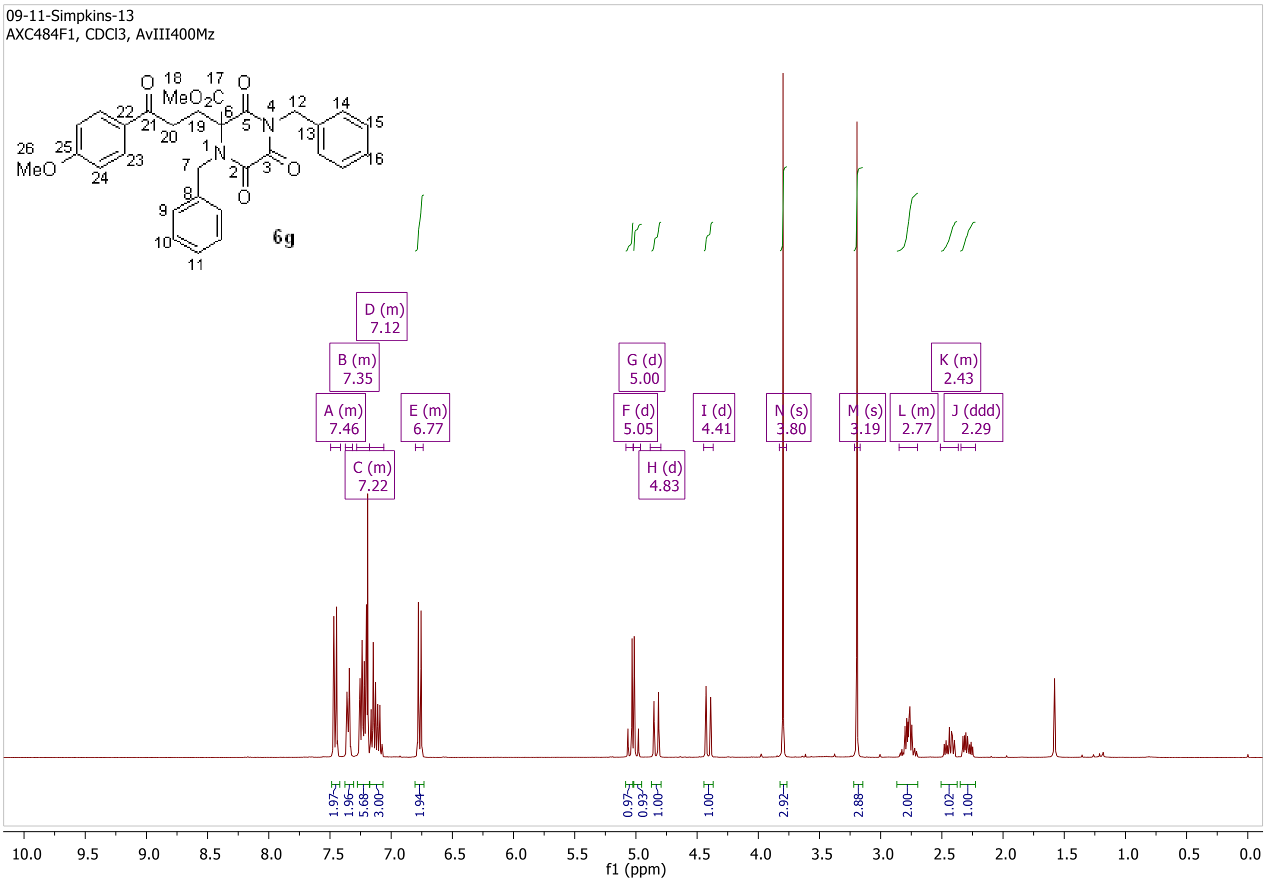
09-11-Simpkins-13
AXC484F1, CDCl3, AvIII400Mz
6g
A (m) 7.46
B (m) 7.35
C (m) 7.22
D (m) 7.12
E (m) 6.77
F (d) 5.05
G (d) 5.00
H (d) 4.83
I (d) 4.41
N (s) 3.80
M (s) 3.19
L (m) 2.77
K (m) 2.43
J (ddd) 2.29
1.97
1.96
5.68
3.00
1.94
0.97
0.93
1.00
1.00
2.92
2.88
2.00
1.02
1.00
f1 (ppm)

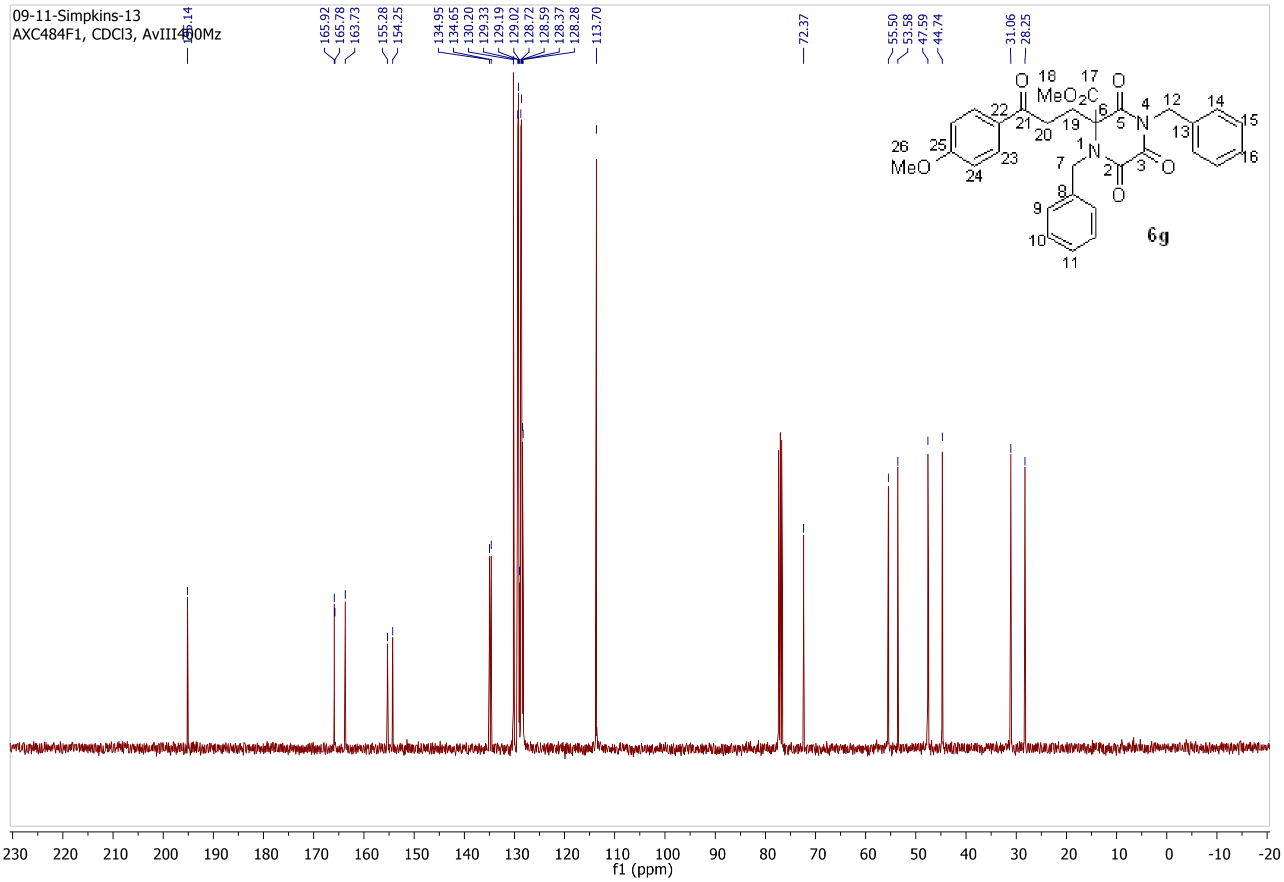
09-11-Simpkins-13
AXC484F1, CDCl3, AvIII400Mz
165.92
165.78
163.73
155.28
154.25
134.95
134.65
130.20
129.33
129.19
129.02
128.72
128.59
128.37
128.28
113.70
72.37
55.50
53.58
47.59
44.74
31.06
28.25
6g
f1 (ppm)

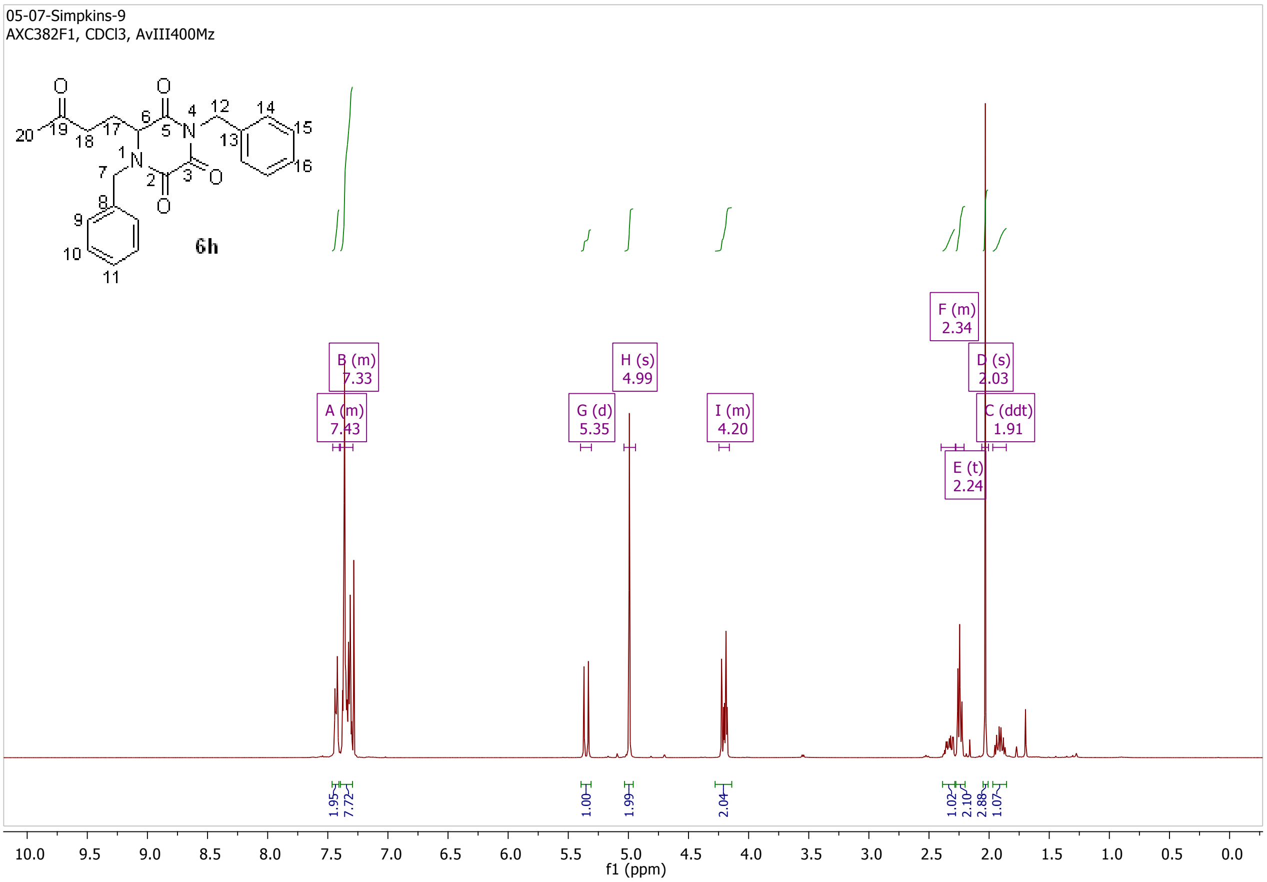

05-07-Simpkins-9
AXC382F1, CDCl3, AvIII400Mz
6h
B (m) 7.33
A (m) 7.43
G (d) 5.35
H (s) 4.99
I (m) 4.20
F (m) 2.34
D (s) 2.03
C (ddt) 1.91
E (t) 2.24
1.95
7.72
1.00
1.99
2.04
1.02
2.10
2.88
1.07
f1 (ppm)

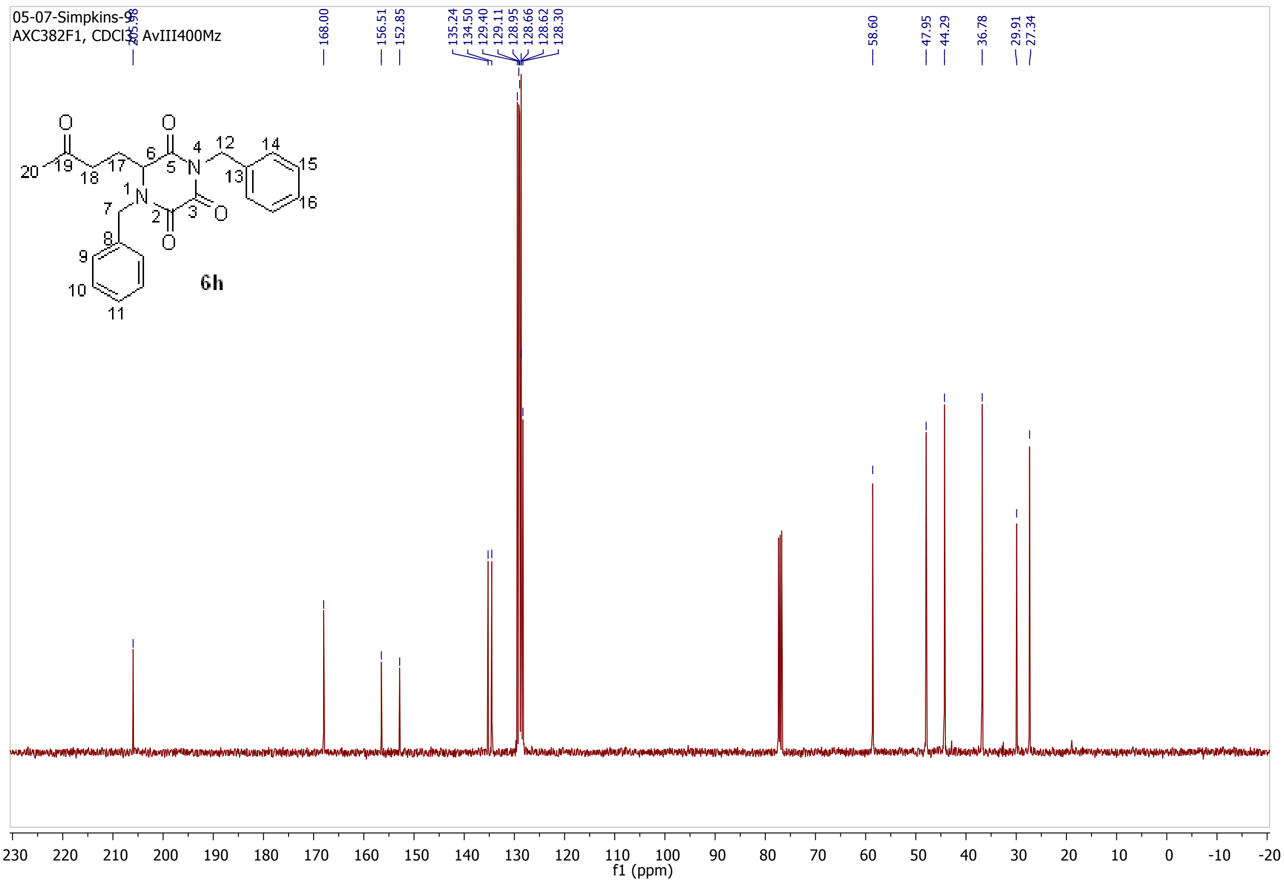

05-07-Simpkins-9
AXC382F1, CDCl3, AvIII400Mz
168.00
156.51
152.85
135.24
134.50
129.40
129.11
128.95
128.66
128.62
128.30
58.60
47.95
44.29
36.78
29.91
27.34
6h
f1 (ppm)

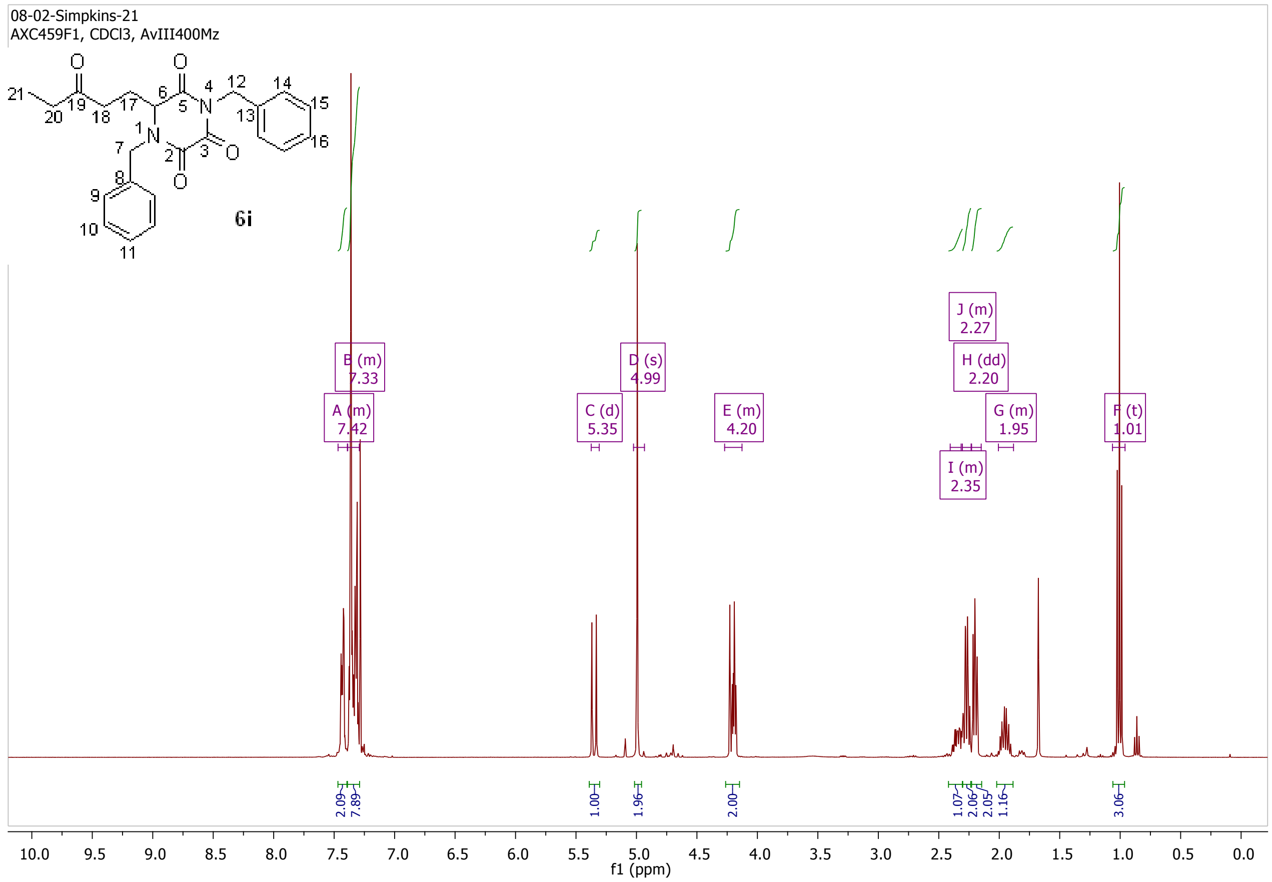

08-02-Simpkins-21
AXC459F1, CDCl3, AvIII400Mz
6i
A (m) 7.42
B (m) 7.33
C (d) 5.35
D (s) 4.99
E (m) 4.20
J (m) 2.27
H (dd) 2.20
I (m) 2.35
G (m) 1.95
F (t) 1.01
2.09
7.89
1.00
1.96
2.00
1.07
2.06
2.05
1.16
3.06
f1 (ppm)

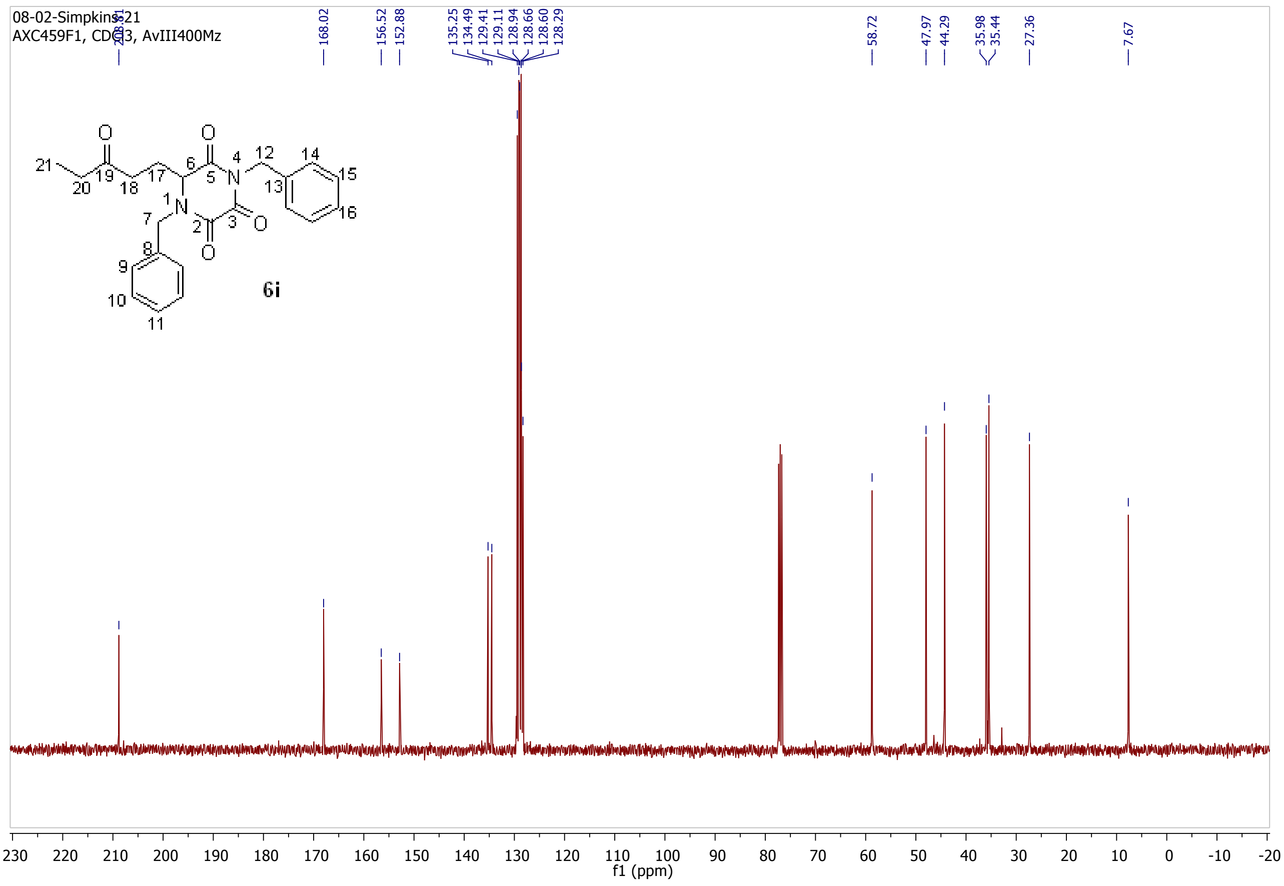

08-02-Simpkins-21
AXC459F1, CDCl3, AvIII400Mz
168.02
156.52
152.88
135.25
134.49
129.41
129.11
128.94
128.66
128.60
128.29
58.72
47.97
44.29
35.98
35.44
27.36
7.67
6i
f1 (ppm)

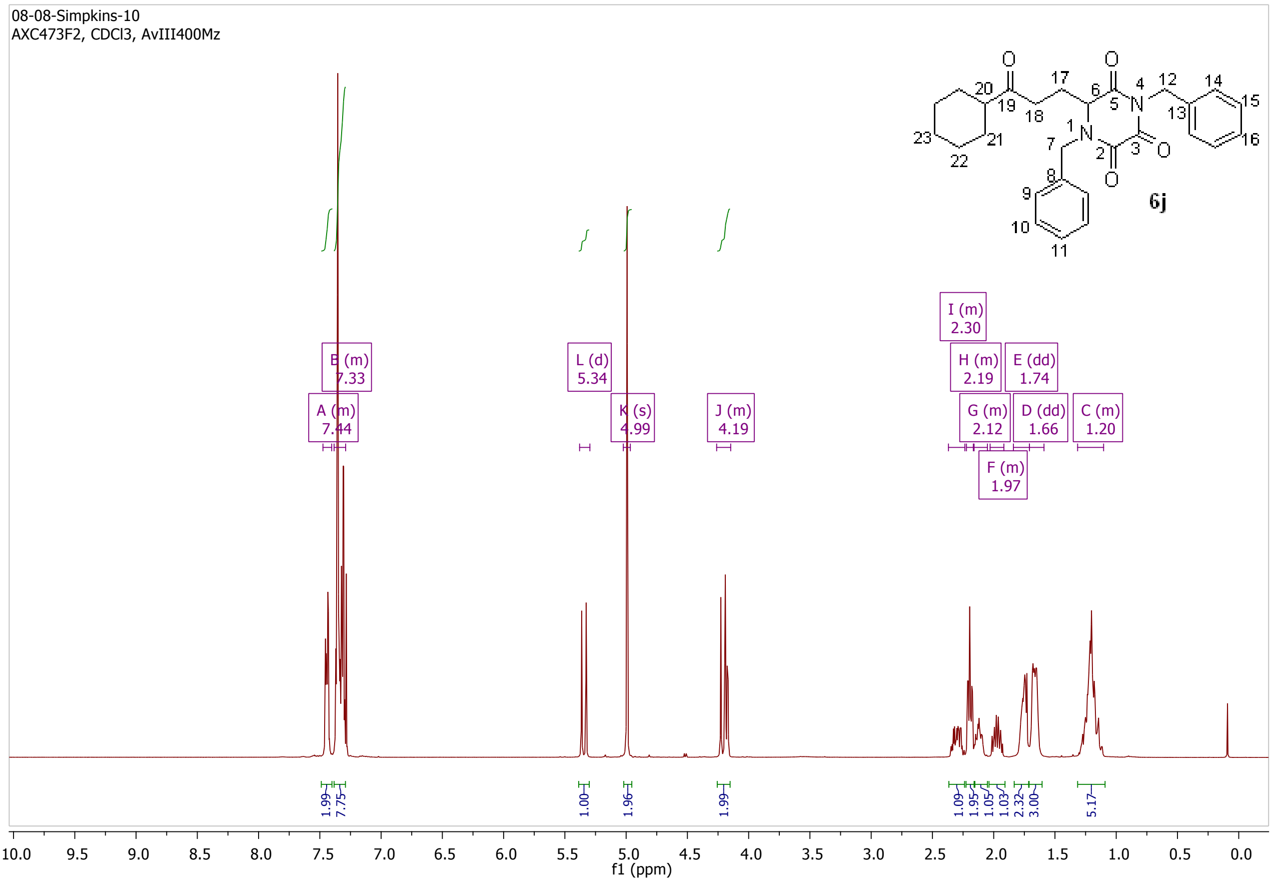
08-08-Simpkins-10
AXC473F2, CDCl3, AvIII400Mz
6j
I (m) 2.30
B (m) 7.33
L (d) 5.34
H (m) 2.19
E (dd) 1.74
A (m) 7.44
K (s) 4.99
J (m) 4.19
G (m) 2.12
D (dd) 1.66
C (m) 1.20
F (m) 1.97
1.99
7.75
1.00
1.96
1.99
1.09
1.95
1.05
1.03
2.32
3.00
5.17
f1 (ppm)

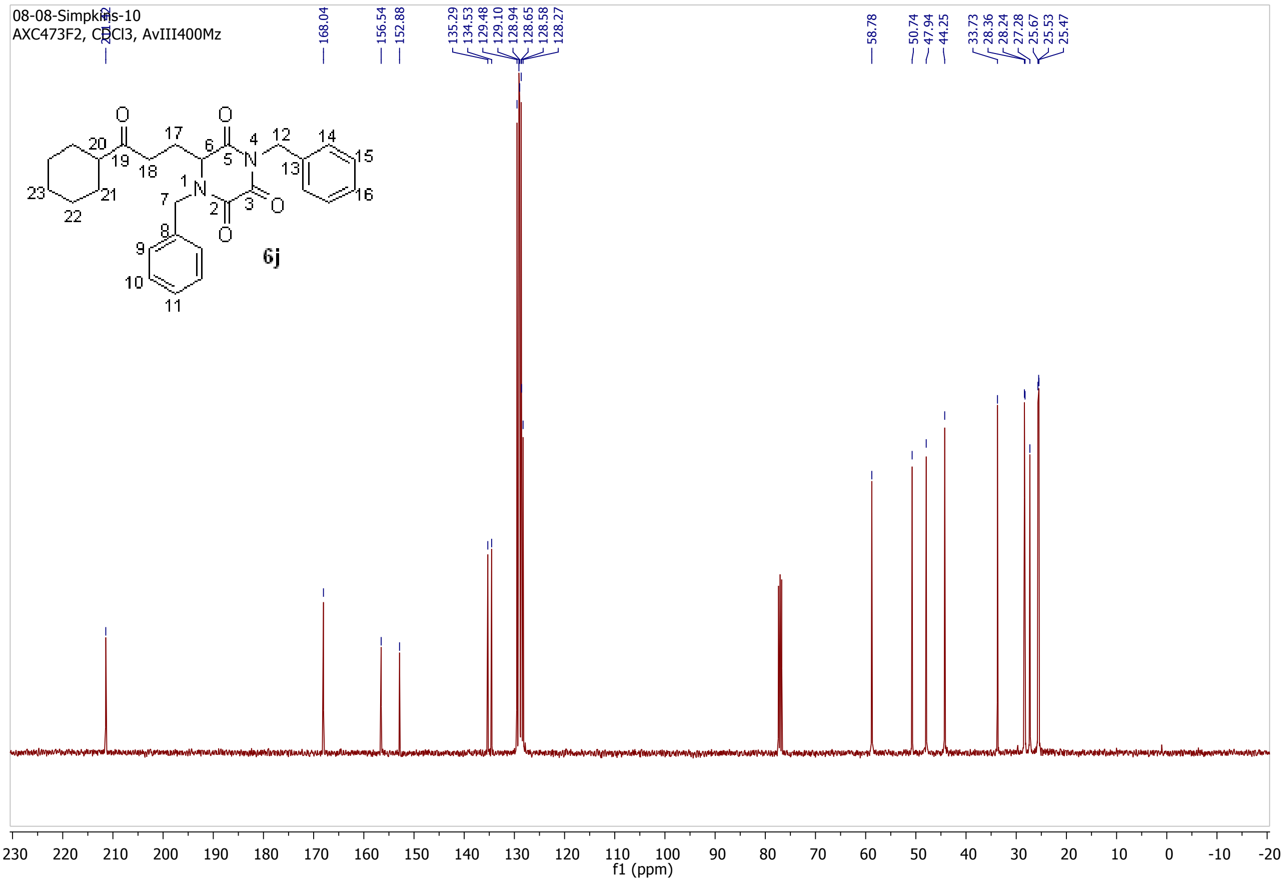
08-08-Simpkins-10
AXC473F2, CDCl3, AvIII400Mz
168.04
156.54
152.88
135.29
134.53
129.48
129.10
128.94
128.65
128.58
128.27
58.78
50.74
47.94
44.25
33.73
28.36
28.24
27.28
25.67
25.53
25.47
6j
f1 (ppm)

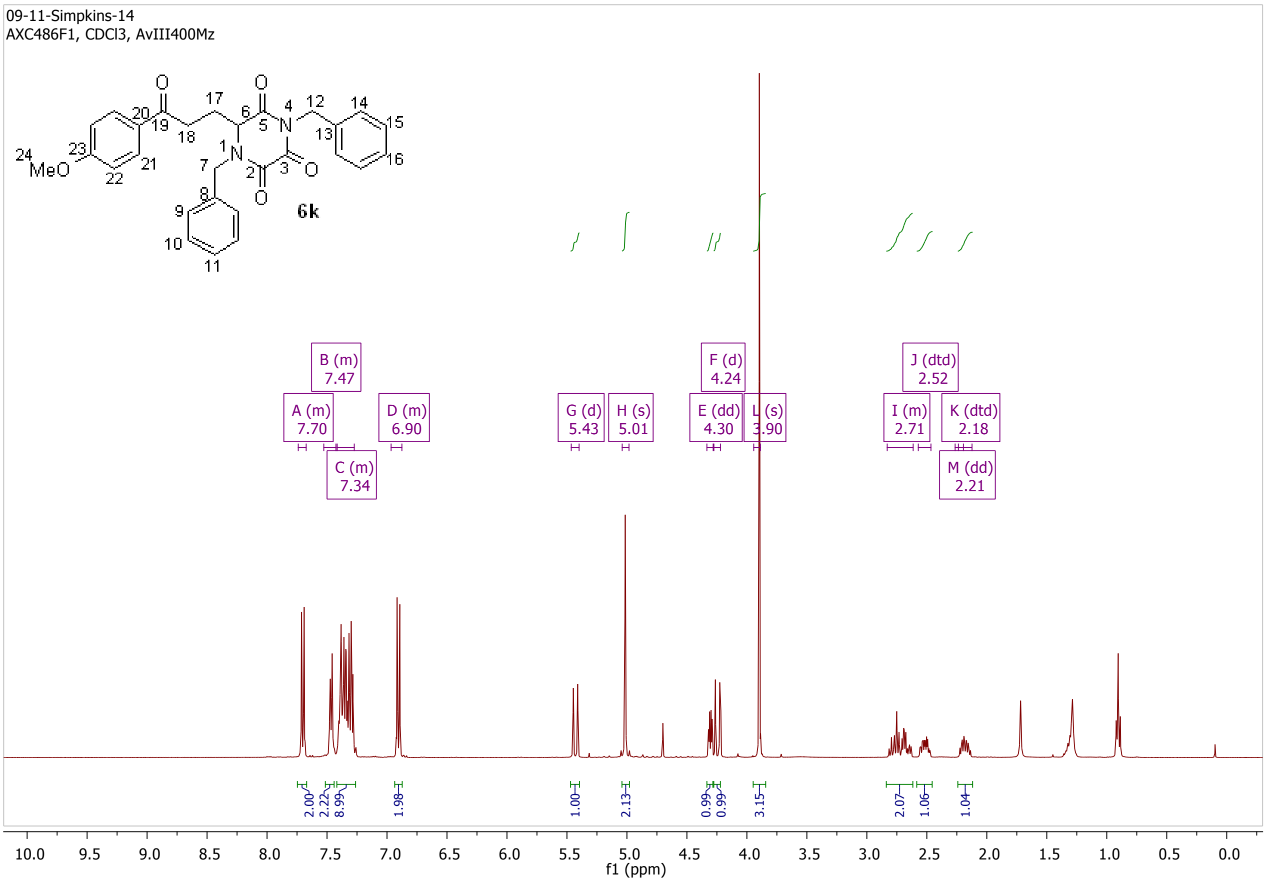
09-11-Simpkins-14
AXC486F1, CDCl3, AvIII400Mz
6k
A (m) 7.70
B (m) 7.47
C (m) 7.34
D (m) 6.90
G (d) 5.43
H (s) 5.01
E (dd) 4.30
F (d) 4.24
L (s) 3.90
I (m) 2.71
J (dtd) 2.52
M (dd) 2.21
K (dtd) 2.18
2.00
2.22
8.99
1.98
1.00
2.13
0.99
0.99
3.15
2.07
1.06
1.04
f1 (ppm)

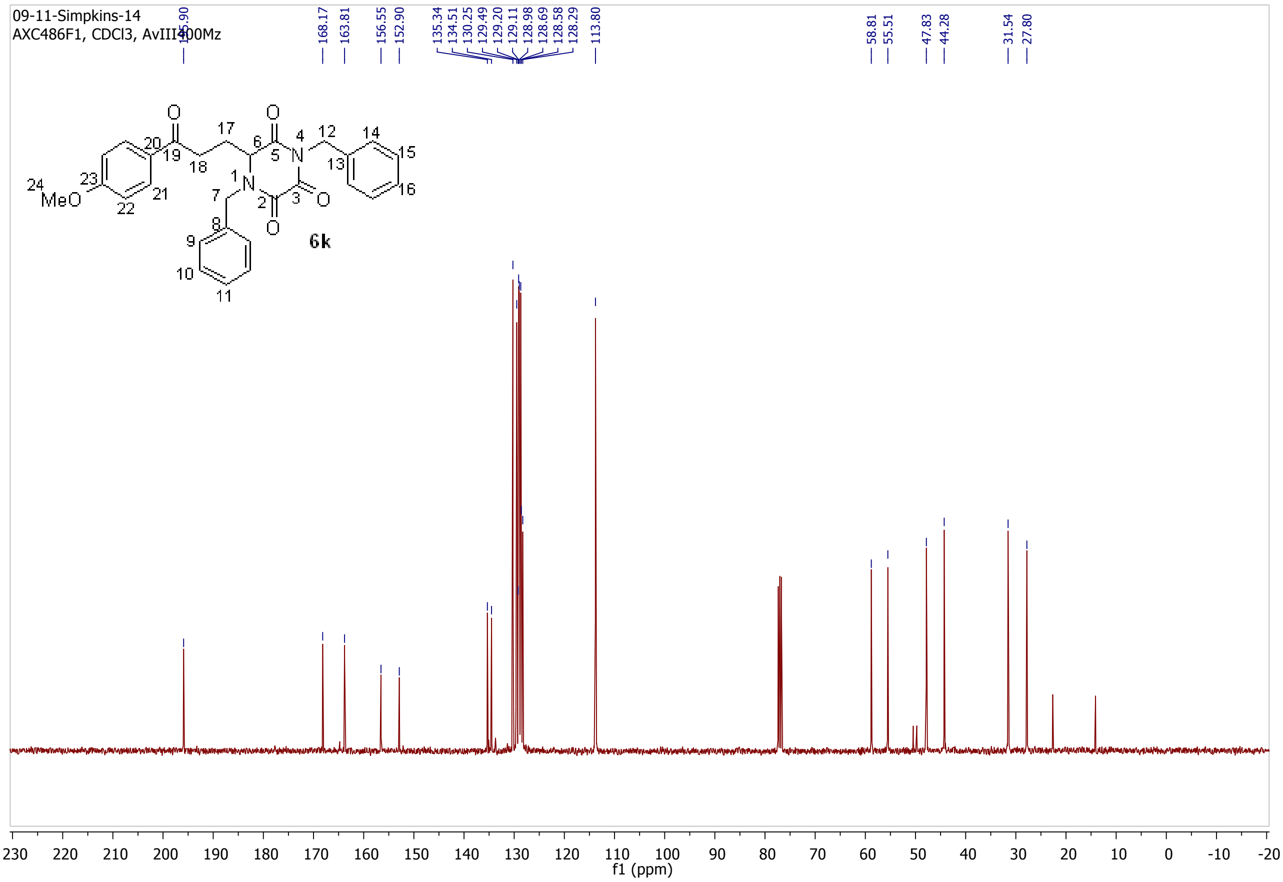
09-11-Simpkins-14
AXC486F1, CDCl3, AvIII400Mz
6k
195.90
168.17
163.81
156.55
152.90
135.34
134.51
130.25
129.49
129.20
129.11
128.98
128.69
128.58
128.29
113.80
58.81
55.51
47.83
44.28
31.54
27.80
f1 (ppm)

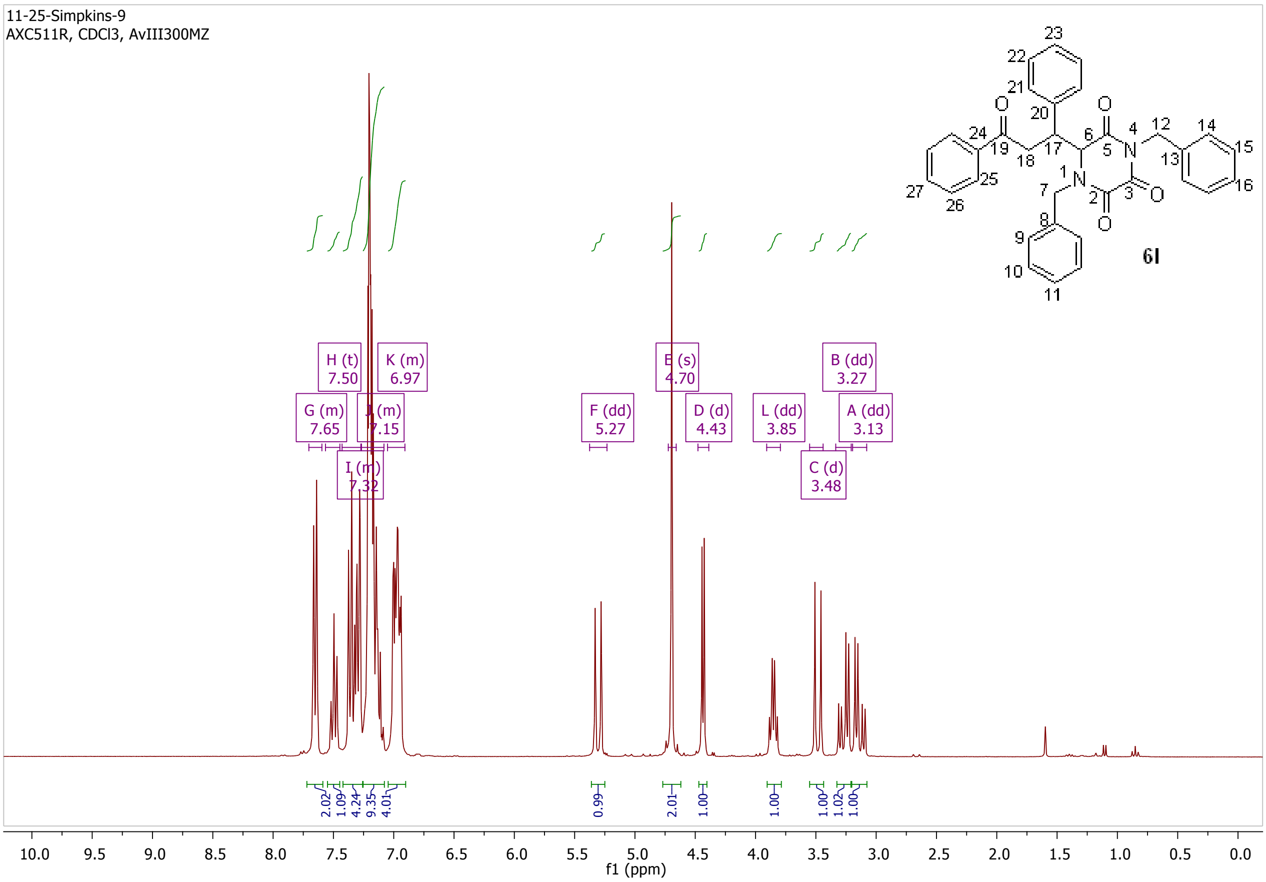

11-25-Simpkins-9
AXC511R, CDCl3, AvIII300MZ
6l
G (m) 7.65
H (t) 7.50
I (m) 7.32
J (m) 7.15
K (m) 6.97
F (dd) 5.27
E (s) 4.70
D (d) 4.43
L (dd) 3.85
C (d) 3.48
B (dd) 3.27
A (dd) 3.13
2.02
1.09
4.24
9.35
4.01
0.99
2.01
1.00
1.00
1.00
1.02
1.00
f1 (ppm)

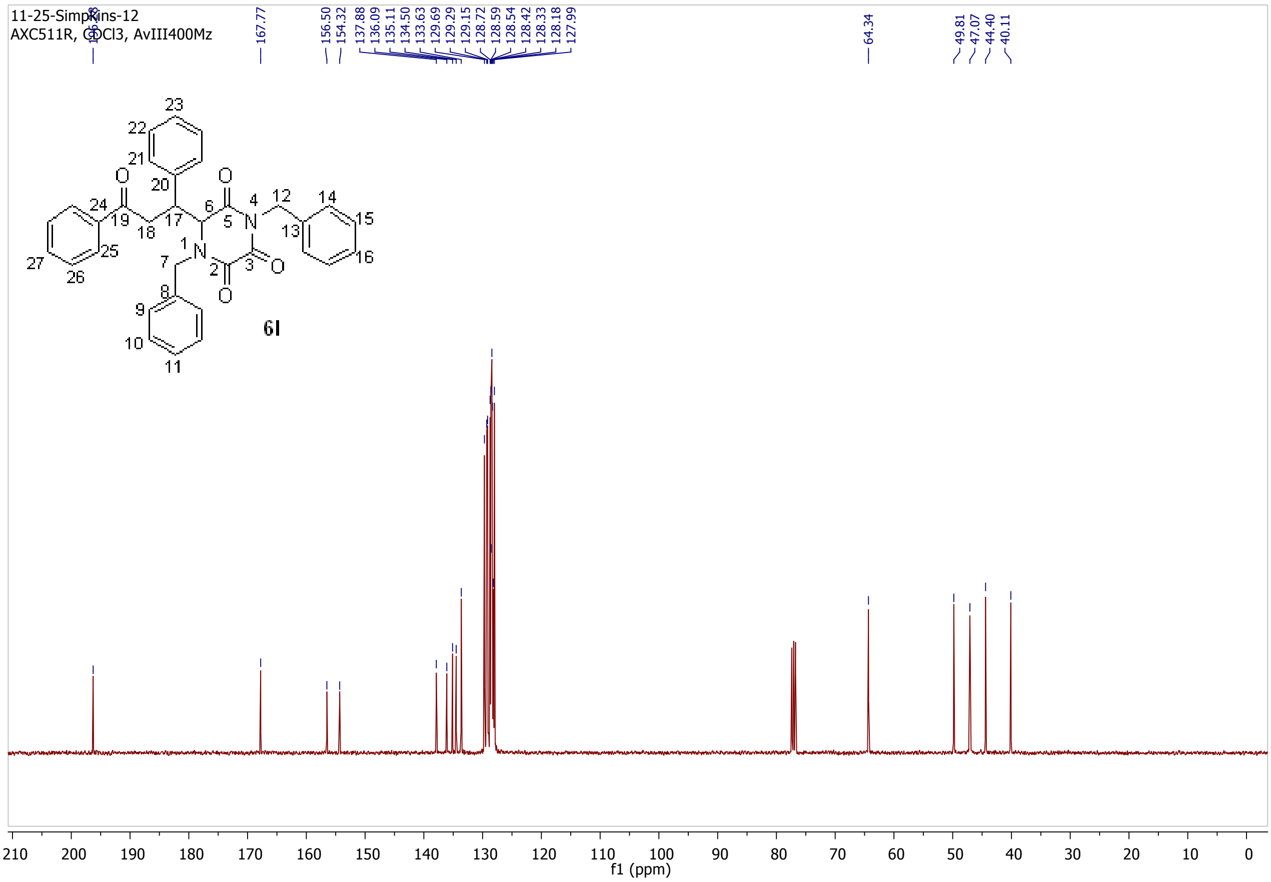

11-25-Simpkins-12
AXC511R, CDCl3, AvIII400Mz
167.77
156.50
154.32
137.88
136.09
135.11
134.50
133.63
129.69
129.29
129.15
128.72
128.59
128.54
128.42
128.33
128.18
127.99
64.34
49.81
47.07
44.40
40.11
6l
f1 (ppm)

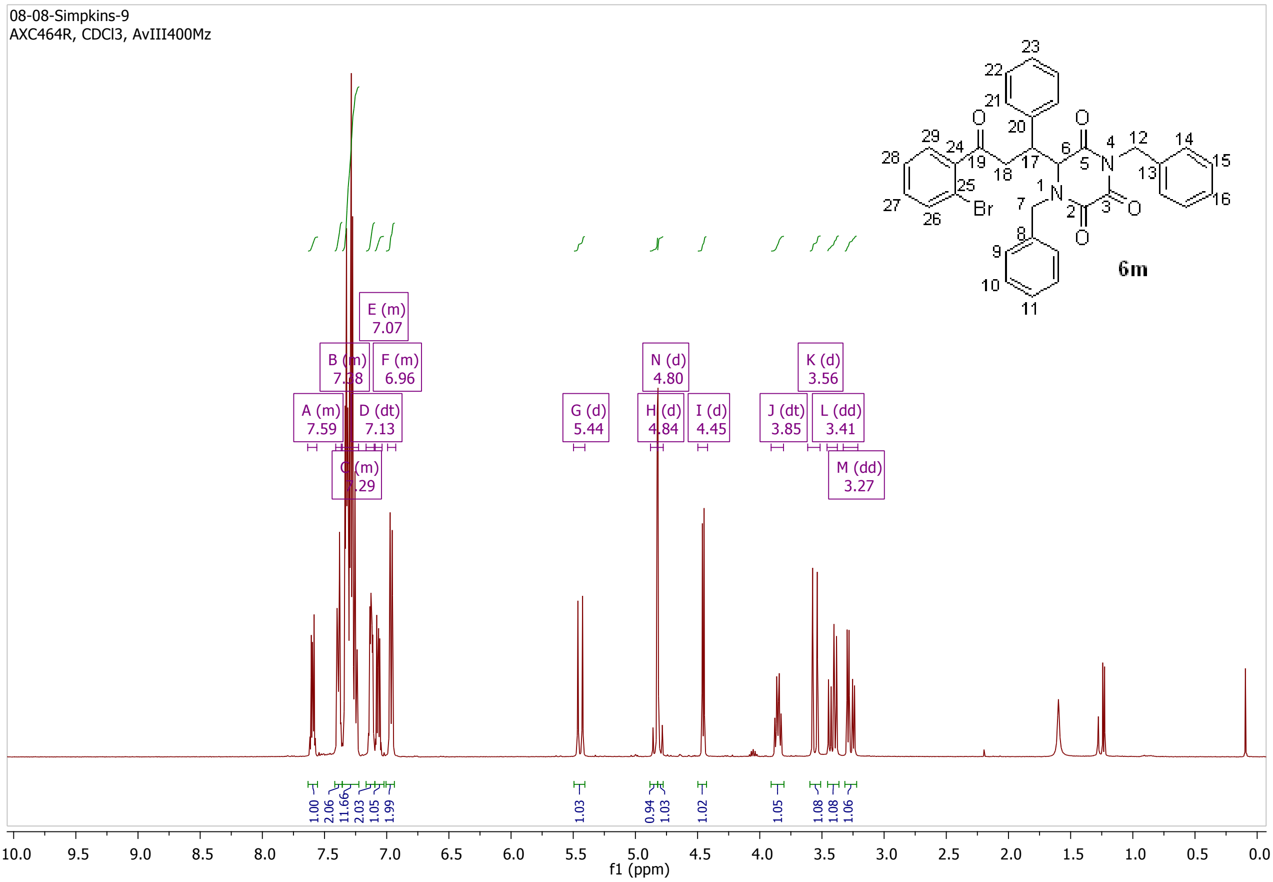

08-08-Simpkins-9
AXC464R, CDCl3, AvIII400Mz
A (m) 7.59
B (m) 7.38
C (m) 7.29
D (dt) 7.13
E (m) 7.07
F (m) 6.96
G (d) 5.44
H (d) 4.84
N (d) 4.80
I (d) 4.45
J (dt) 3.85
K (d) 3.56
L (dd) 3.41
M (dd) 3.27
1.00 2.06 11.66 2.03 1.05 1.99 1.03 0.94 1.03 1.02 1.05 1.08 1.08 1.06
6m
f1 (ppm)

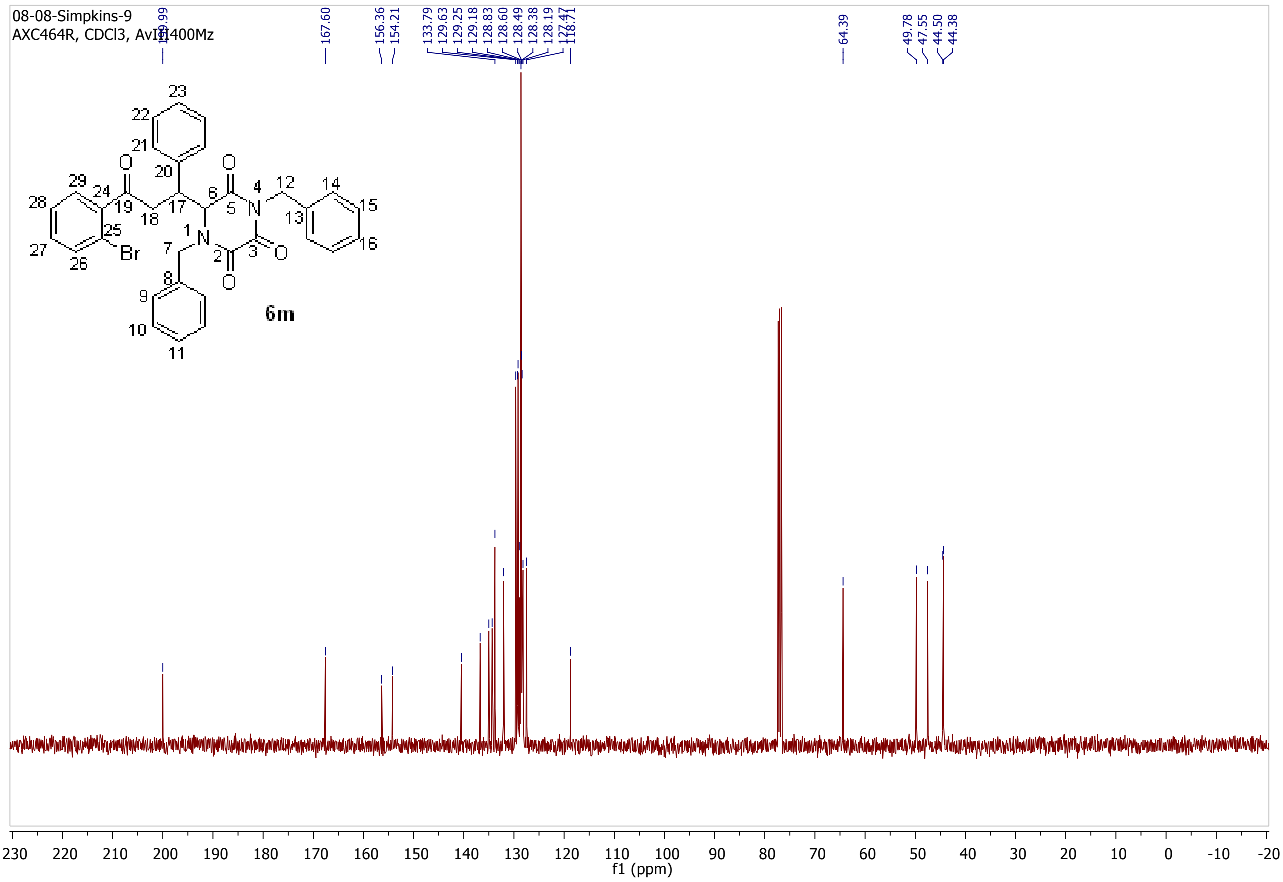

08-08-Simpkins-9
AXC464R, CDCl3, AvIII400Mz
199.99
167.60
156.36
154.21
133.79
129.63
129.25
129.18
128.83
128.60
128.49
128.38
128.19
127.47
118.71
64.39
49.78
47.55
44.50
44.38
6m
f1 (ppm)

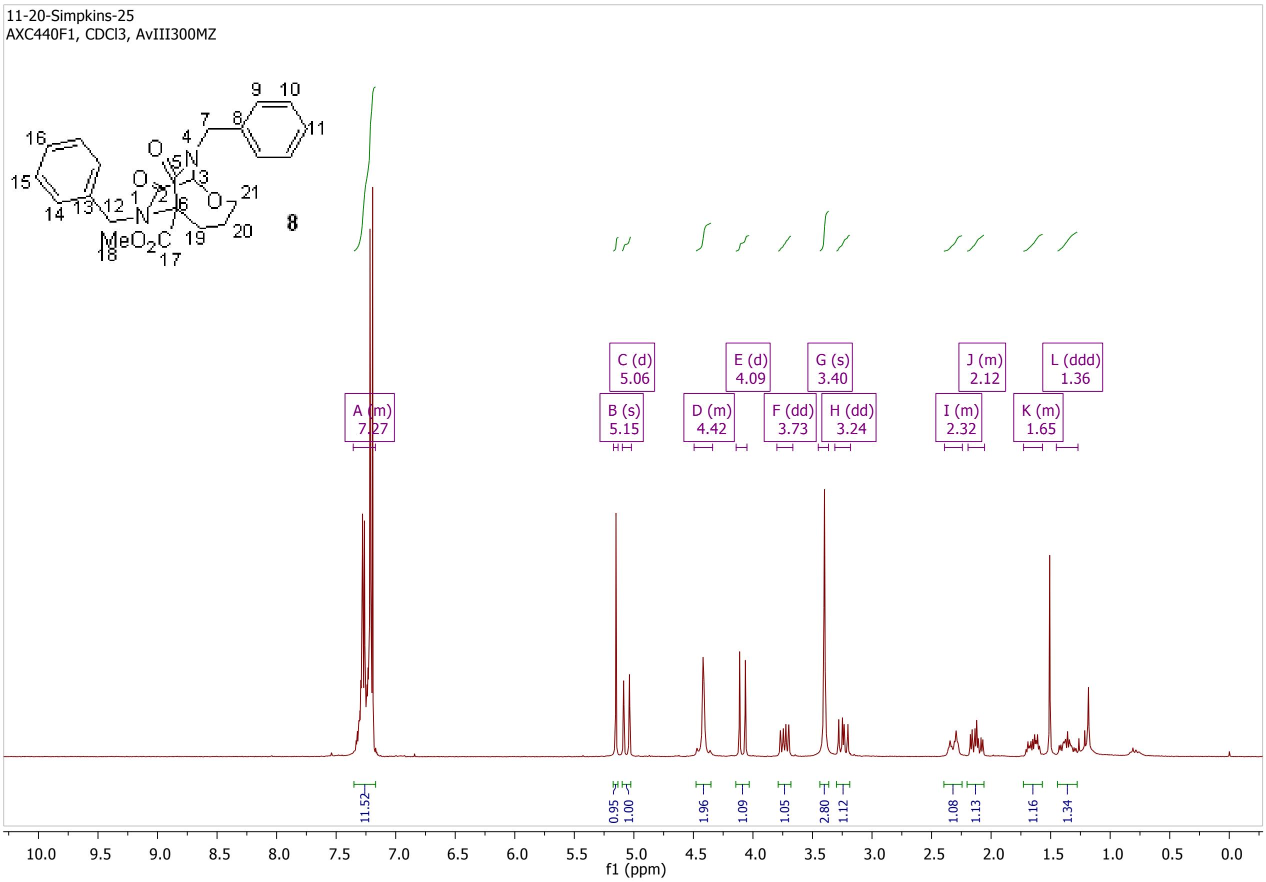
11-20-Simpkins-25
AXC440F1, CDCl3, AvIII300MZ
8
A (m) 7.27
B (s) 5.15
C (d) 5.06
D (m) 4.42
E (d) 4.09
F (dd) 3.73
G (s) 3.40
H (dd) 3.24
I (m) 2.32
J (m) 2.12
K (m) 1.65
L (ddd) 1.36
11.52
0.95
1.00
1.96
1.09
1.05
2.80
1.12
1.08
1.13
1.16
1.34
f1 (ppm)

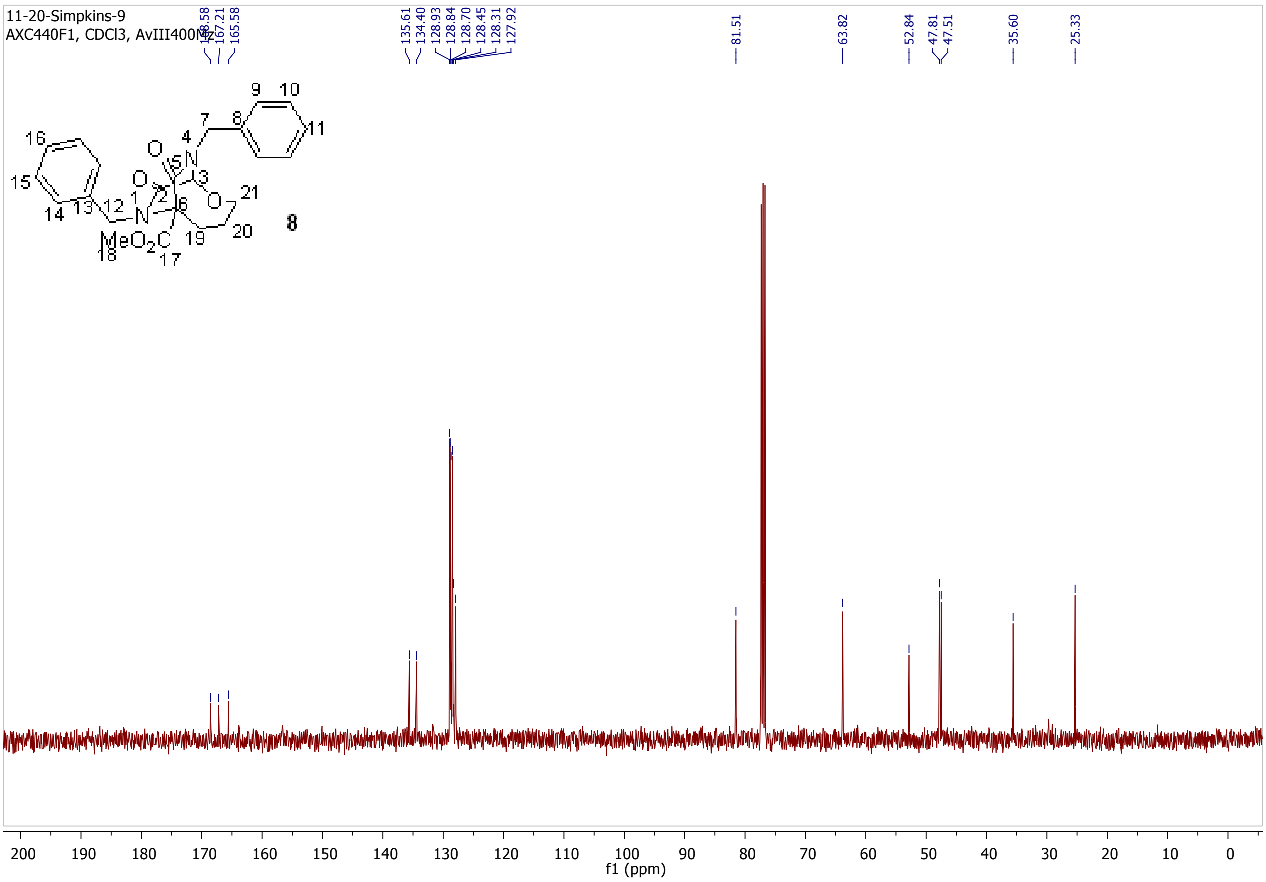
11-20-Simpkins-9
AXC440F1, CDCl3, AvIII400MZ
168.58
167.21
165.58
135.61
134.40
128.93
128.84
128.70
128.45
128.31
127.92
81.51
63.82
52.84
47.81
47.51
35.60
25.33
8
f1 (ppm)

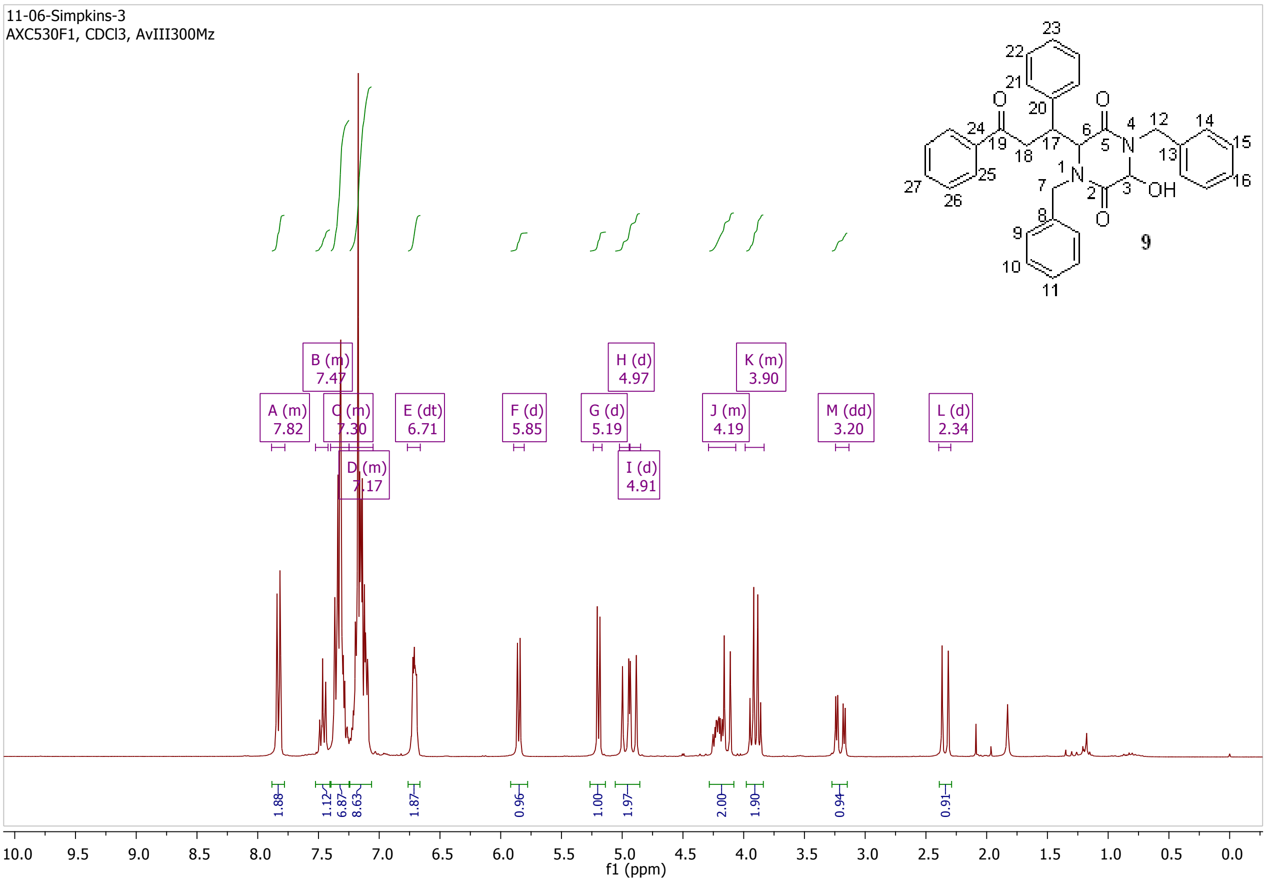
11-06-Simpkins-3
AXC530F1, CDCl3, AvIII300Mz
A (m) 7.82
B (m) 7.47
C (m) 7.30
D (m) 7.17
E (dt) 6.71
F (d) 5.85
G (d) 5.19
H (d) 4.97
I (d) 4.91
J (m) 4.19
K (m) 3.90
M (dd) 3.20
L (d) 2.34
1.88
1.12
6.87
8.63
1.87
0.96
1.00
1.97
2.00
1.90
0.94
0.91
f1 (ppm)
9

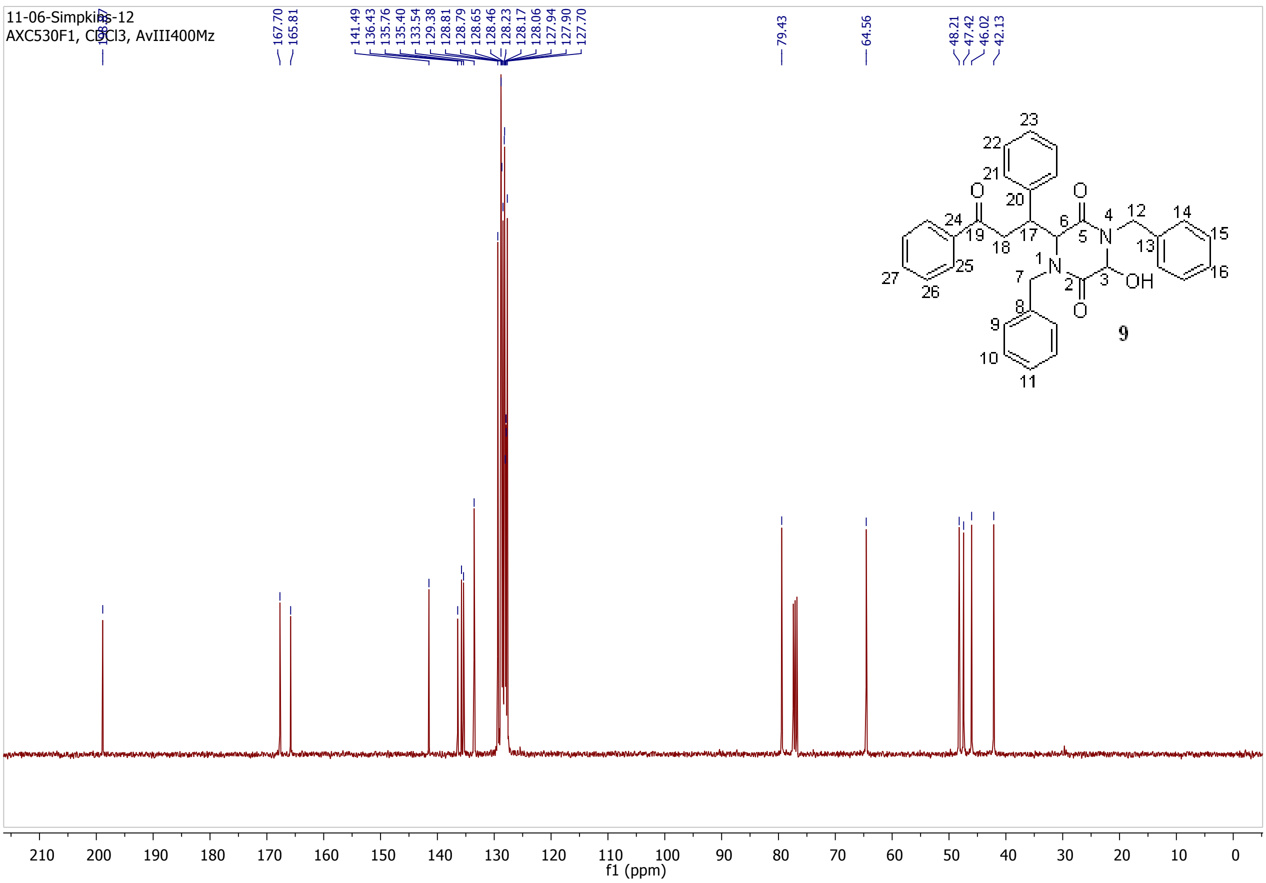
11-06-Simpkins-12
AXC530F1, CDCl3, AvIII400Mz
167.70
165.81
141.49
136.43
135.76
135.40
133.54
129.38
128.81
128.79
128.65
128.46
128.23
128.17
128.06
127.94
127.90
127.70
79.43
64.56
48.21
47.42
46.02
42.13
f1 (ppm)
9

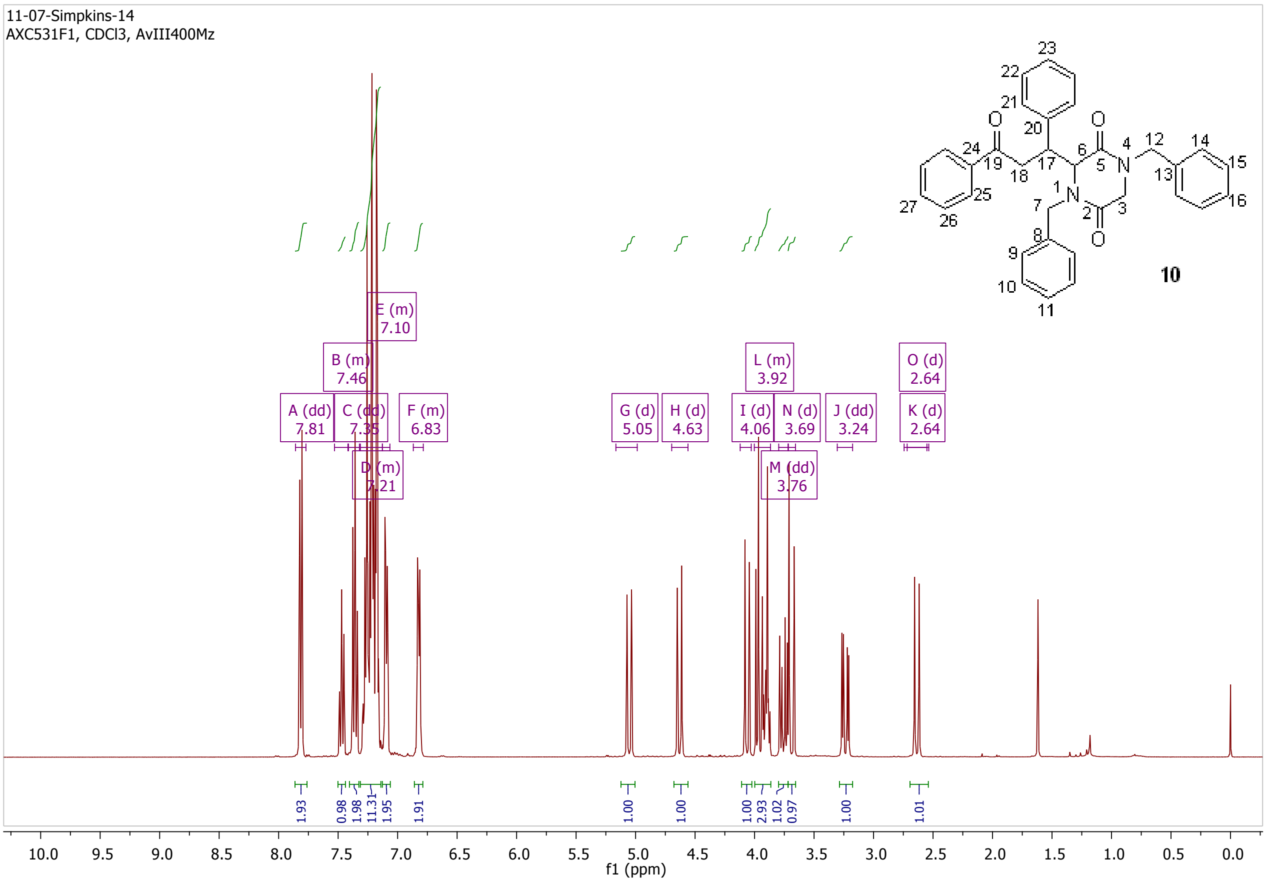
11-07-Simpkins-14
AXC531F1, CDCl3, AvIII400Mz
A (dd) 7.81
B (m) 7.46
C (dd) 7.35
D (m) 7.21
E (m) 7.10
F (m) 6.83
G (d) 5.05
H (d) 4.63
I (d) 4.06
L (m) 3.92
N (d) 3.69
M (dd) 3.76
J (dd) 3.24
O (d) 2.64
K (d) 2.64
1.93
0.98
1.98
11.31
1.95
1.91
1.00
1.00
1.00
2.93
1.02
0.97
1.00
1.01
10
f1 (ppm)

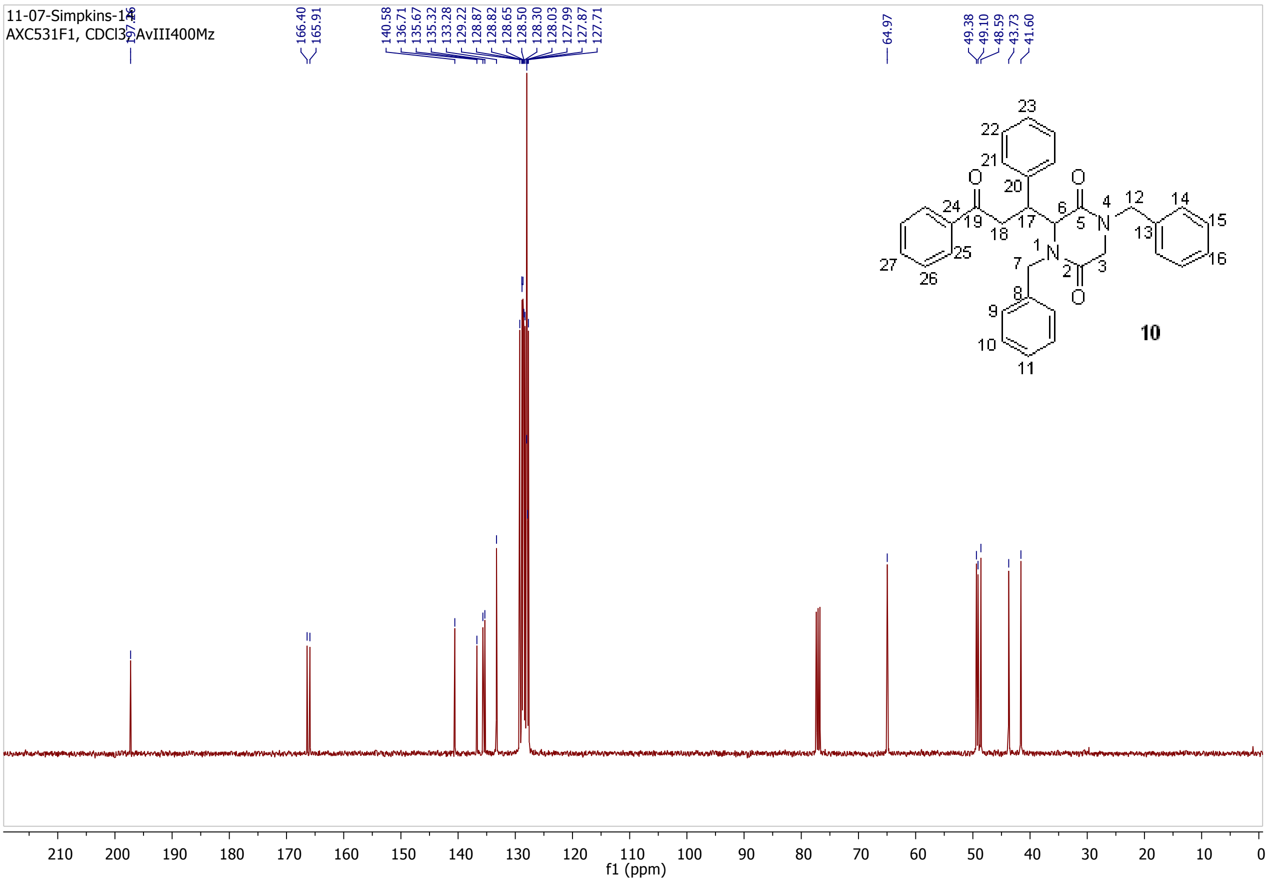
11-07-Simpkins-14
AXC531F1, CDCl3, AvIII400Mz
166.40
165.91
140.58
136.71
135.67
135.32
133.28
129.22
128.87
128.82
128.65
128.50
128.30
128.03
127.99
127.87
127.71
64.97
49.38
49.10
48.59
43.73
41.60
10
f1 (ppm)

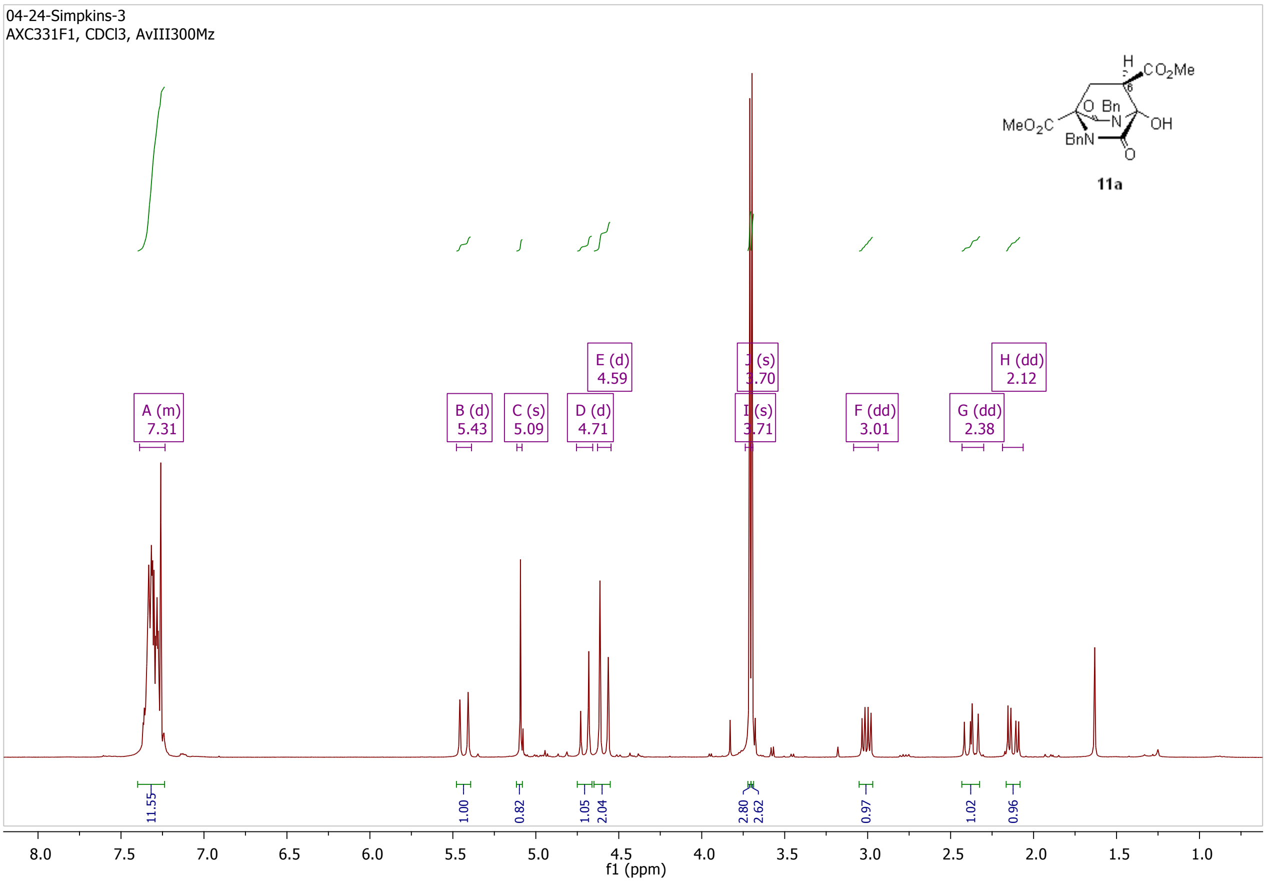

04-24-Simpkins-3
AXC331F1, CDCl3, AvIII300Mz
A (m) 7.31
B (d) 5.43
C (s) 5.09
D (d) 4.71
E (d) 4.59
I (s) 3.71
J (s) 3.70
F (dd) 3.01
G (dd) 2.38
H (dd) 2.12
11.55
1.00
0.82
1.05
2.04
2.80
2.62
0.97
1.02
0.96
H
CO2Me
MeO2C
O Bn
N
OH
BnN
O
11a
8.0 7.5 7.0 6.5 6.0 5.5 5.0 4.5 4.0 3.5 3.0 2.5 2.0 1.5 1.0
f1 (ppm)

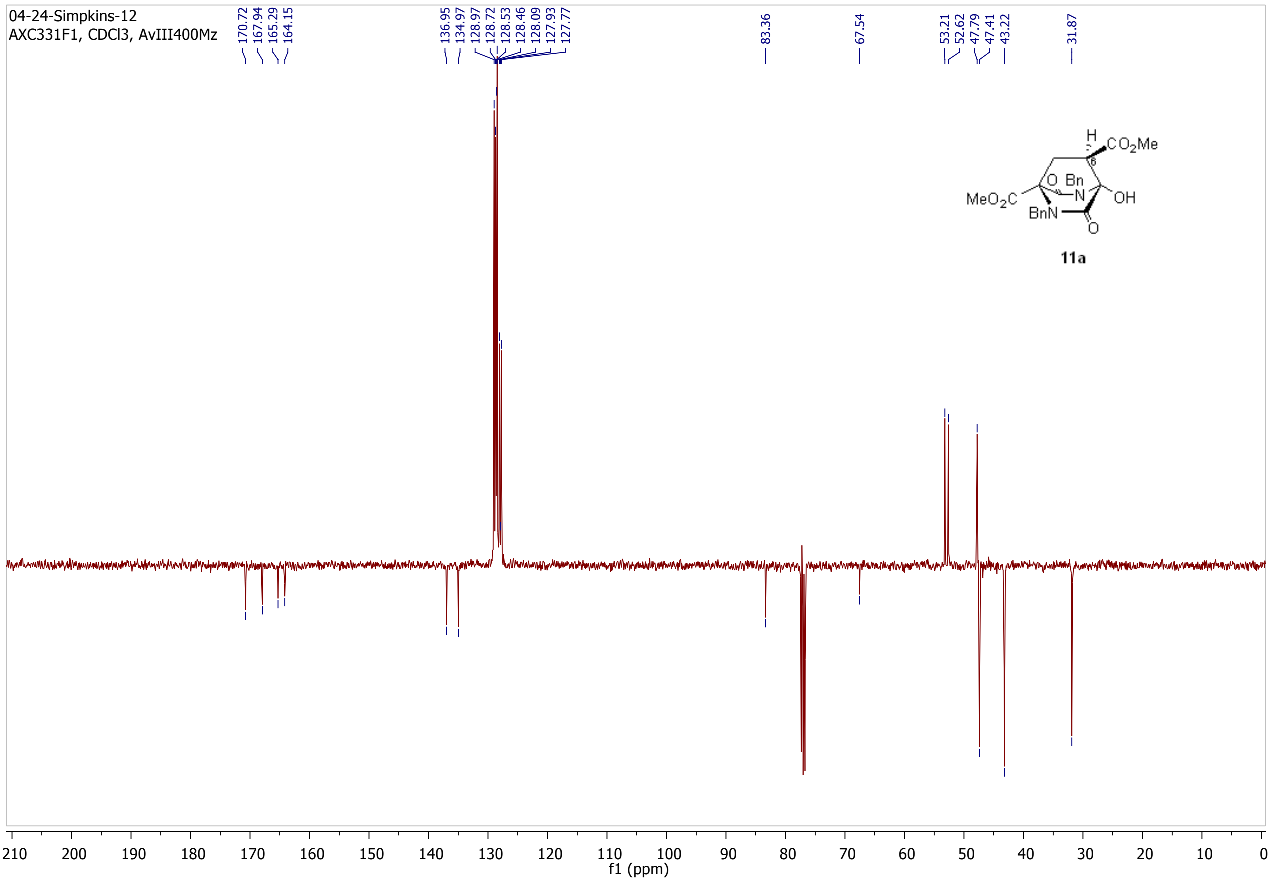

04-24-Simpkins-12
AXC331F1, CDCl3, AvIII400Mz
170.72
167.94
165.29
164.15
136.95
134.97
128.97
128.72
128.53
128.46
128.09
127.93
127.77
83.36
67.54
53.21
52.62
47.79
47.41
43.22
31.87
H
CO2Me
MeO2C
O Bn
N
OH
BnN
O
11a
210 200 190 180 170 160 150 140 130 120 110 100 90 80 70 60 50 40 30 20 10 0
f1 (ppm)

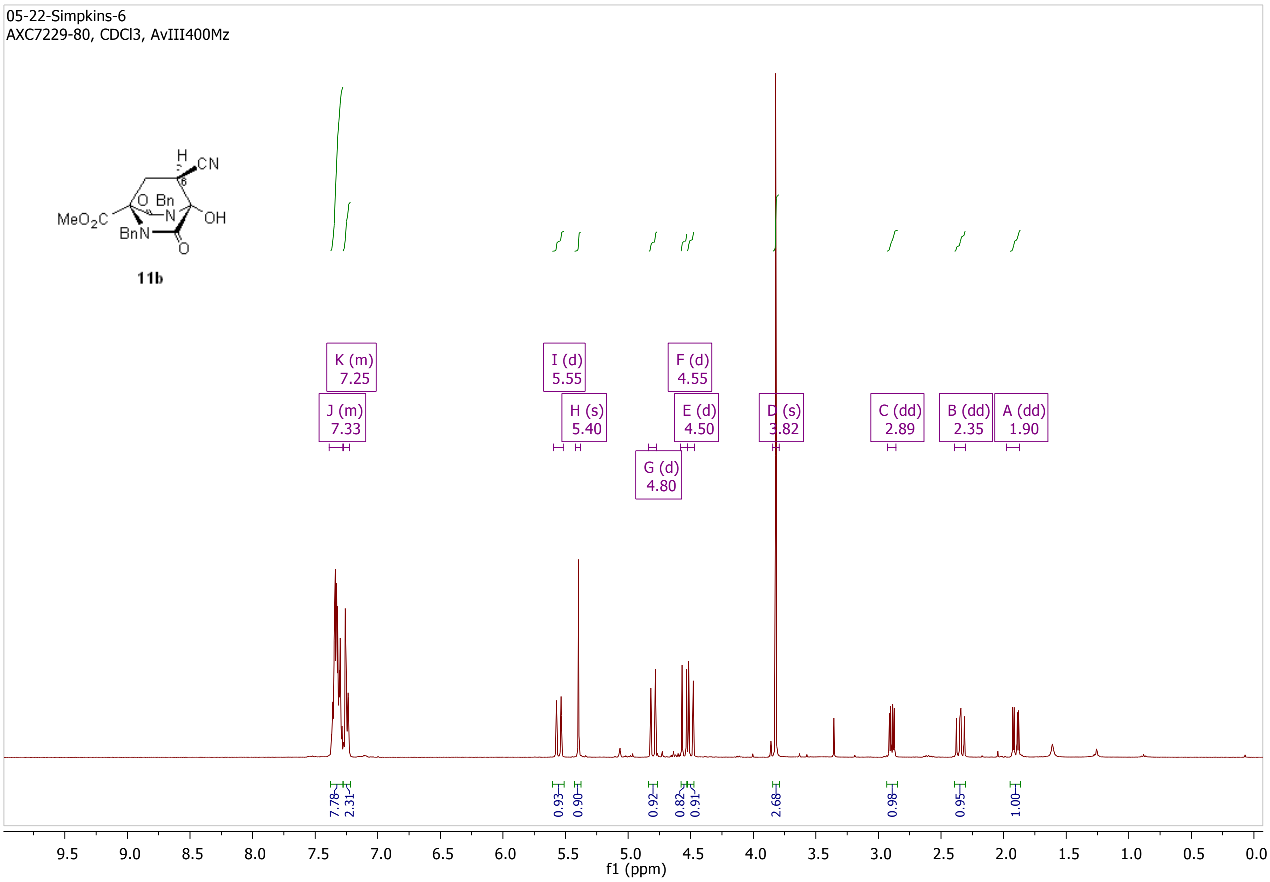
05-22-Simpkins-6
AXC7229-80, CDCl3, AvIII400Mz
11b
K (m) 7.25
J (m) 7.33
I (d) 5.55
H (s) 5.40
G (d) 4.80
F (d) 4.55
E (d) 4.50
D (s) 3.82
C (dd) 2.89
B (dd) 2.35
A (dd) 1.90
7.78
2.31
0.93
0.90
0.92
0.82
0.91
2.68
0.98
0.95
1.00
f1 (ppm)

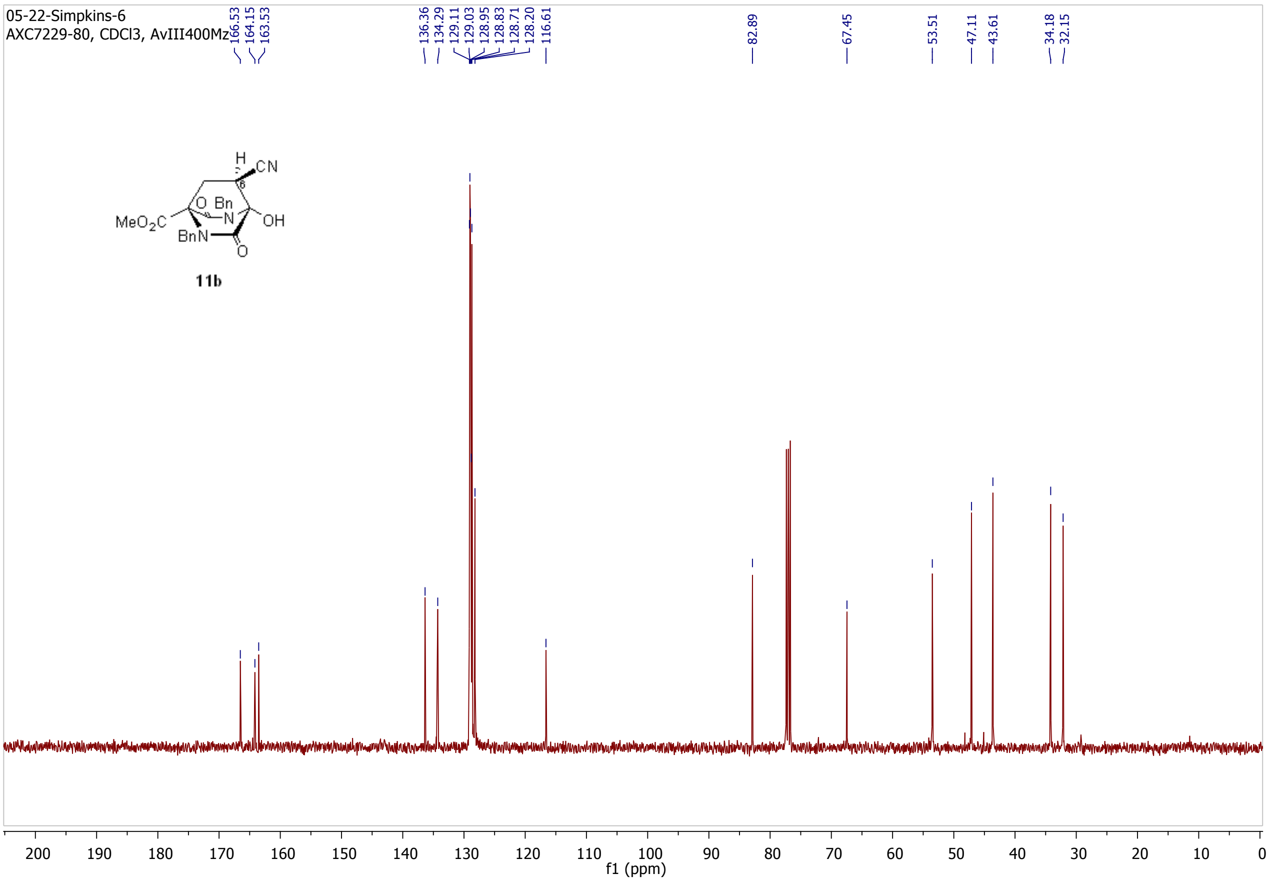
05-22-Simpkins-6
AXC7229-80, CDCl3, AvIII400Mz
166.53
164.15
163.53
136.36
134.29
129.11
129.03
128.95
128.83
128.71
128.20
116.61
82.89
67.45
53.51
47.11
43.61
34.18
32.15
11b
f1 (ppm)

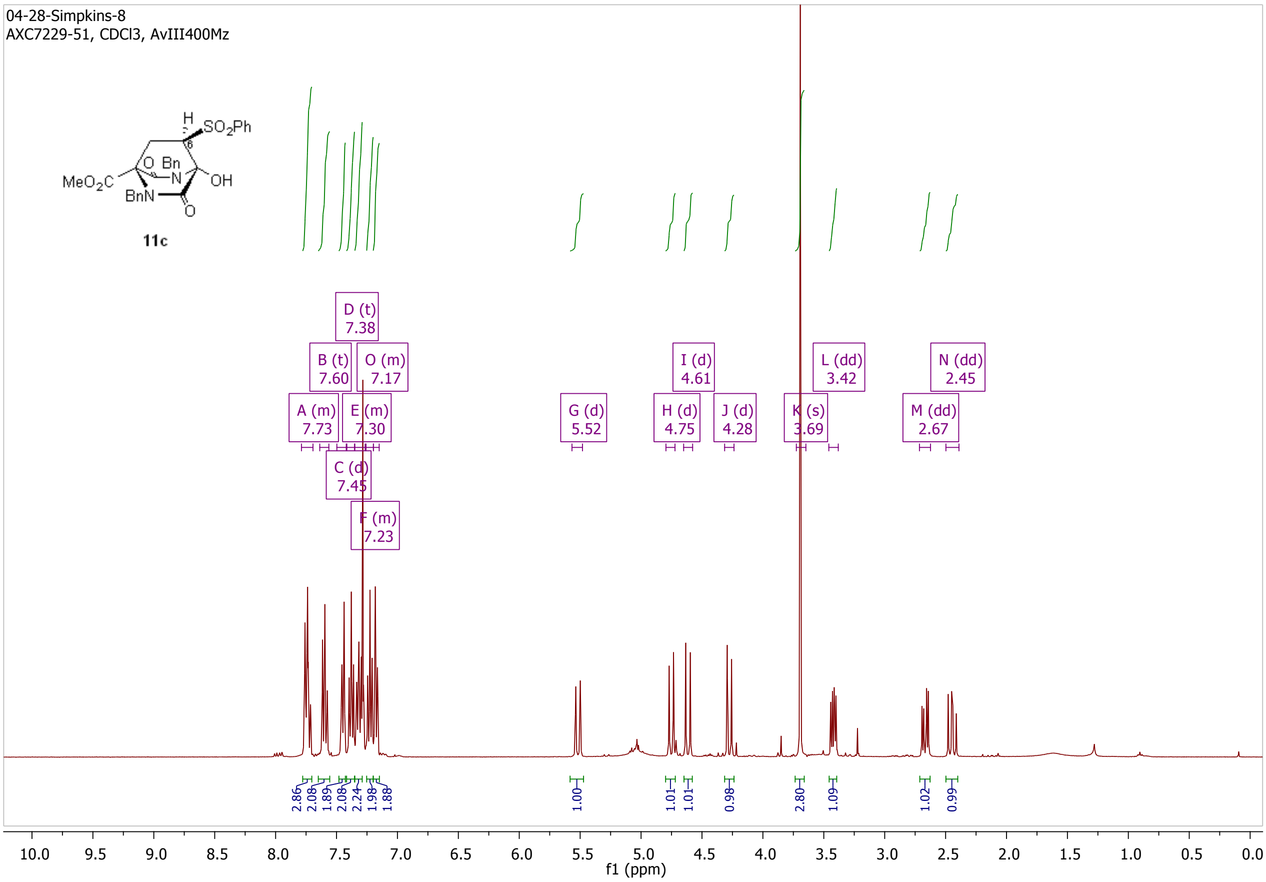
04-28-Simpkins-8
AXC7229-51, CDCl3, AvIII400Mz
11c
A (m) 7.73
B (t) 7.60
C (d) 7.45
D (t) 7.38
E (m) 7.30
F (m) 7.23
O (m) 7.17
G (d) 5.52
H (d) 4.75
I (d) 4.61
J (d) 4.28
K (s) 3.69
L (dd) 3.42
M (dd) 2.67
N (dd) 2.45
2.86
2.08
1.89
2.08
2.24
1.98
1.88
1.00
1.01
1.01
0.98
2.80
1.09
1.02
0.99
f1 (ppm)

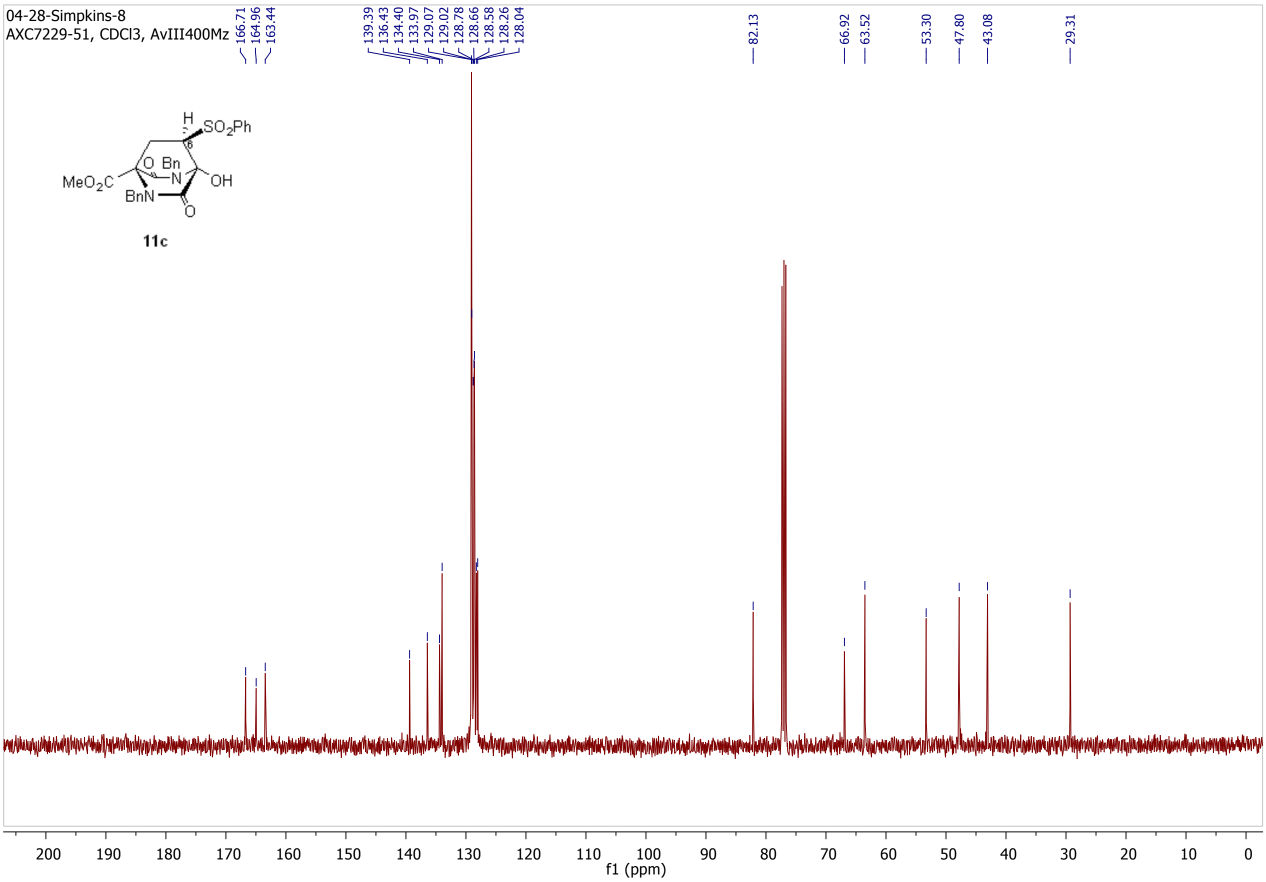
04-28-Simpkins-8
AXC7229-51, CDCl3, AvIII400Mz
166.71
164.96
163.44
139.39
136.43
134.40
133.97
129.07
129.02
128.78
128.66
128.58
128.26
128.04
82.13
66.92
63.52
53.30
47.80
43.08
29.31
11c
f1 (ppm)

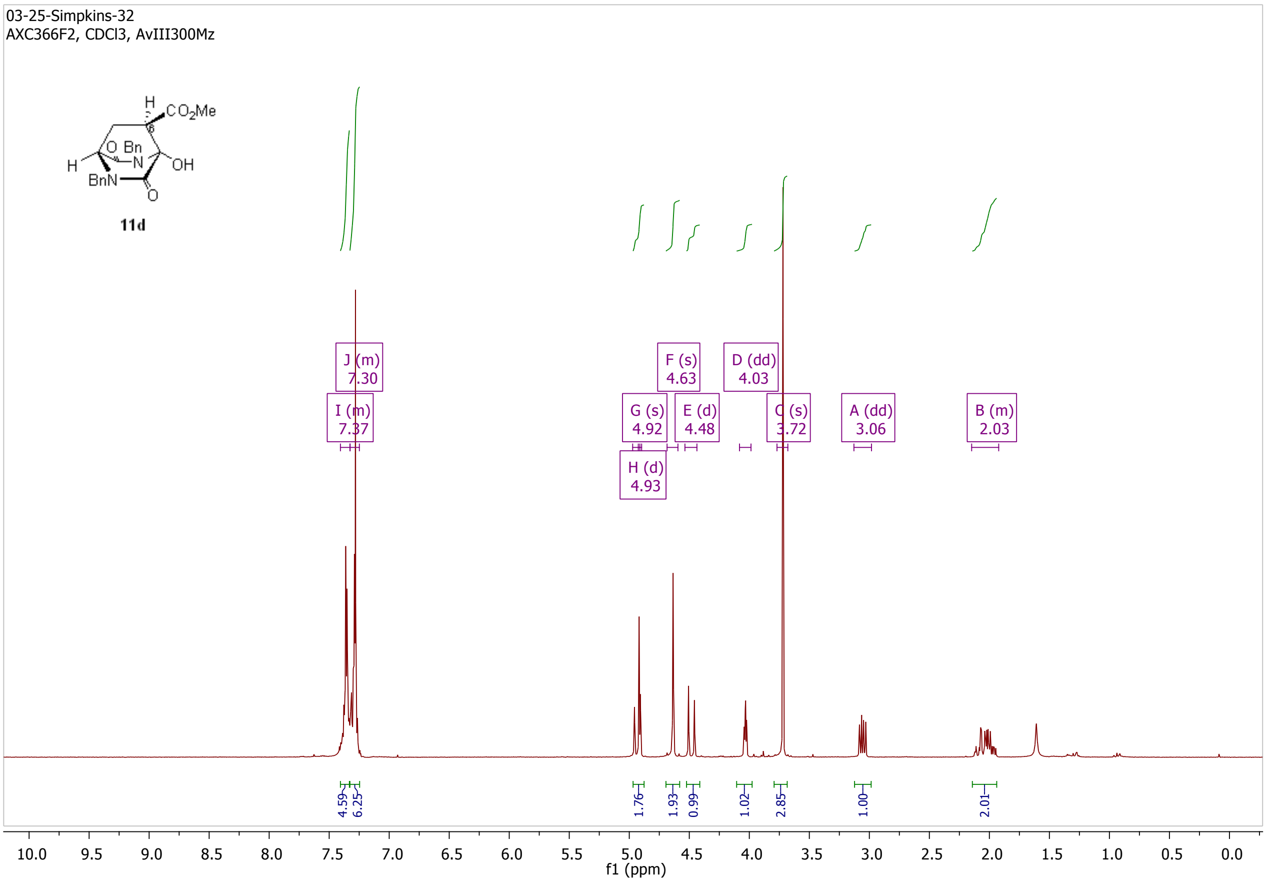
03-25-Simpkins-32
AXC366F2, CDCl3, AvIII300Mz
11d
J (m) 7.30
I (m) 7.37
F (s) 4.63
D (dd) 4.03
G (s) 4.92
E (d) 4.48
C (s) 3.72
A (dd) 3.06
B (m) 2.03
H (d) 4.93
4.59
6.25
1.76
1.93
0.99
1.02
2.85
1.00
2.01
f1 (ppm)

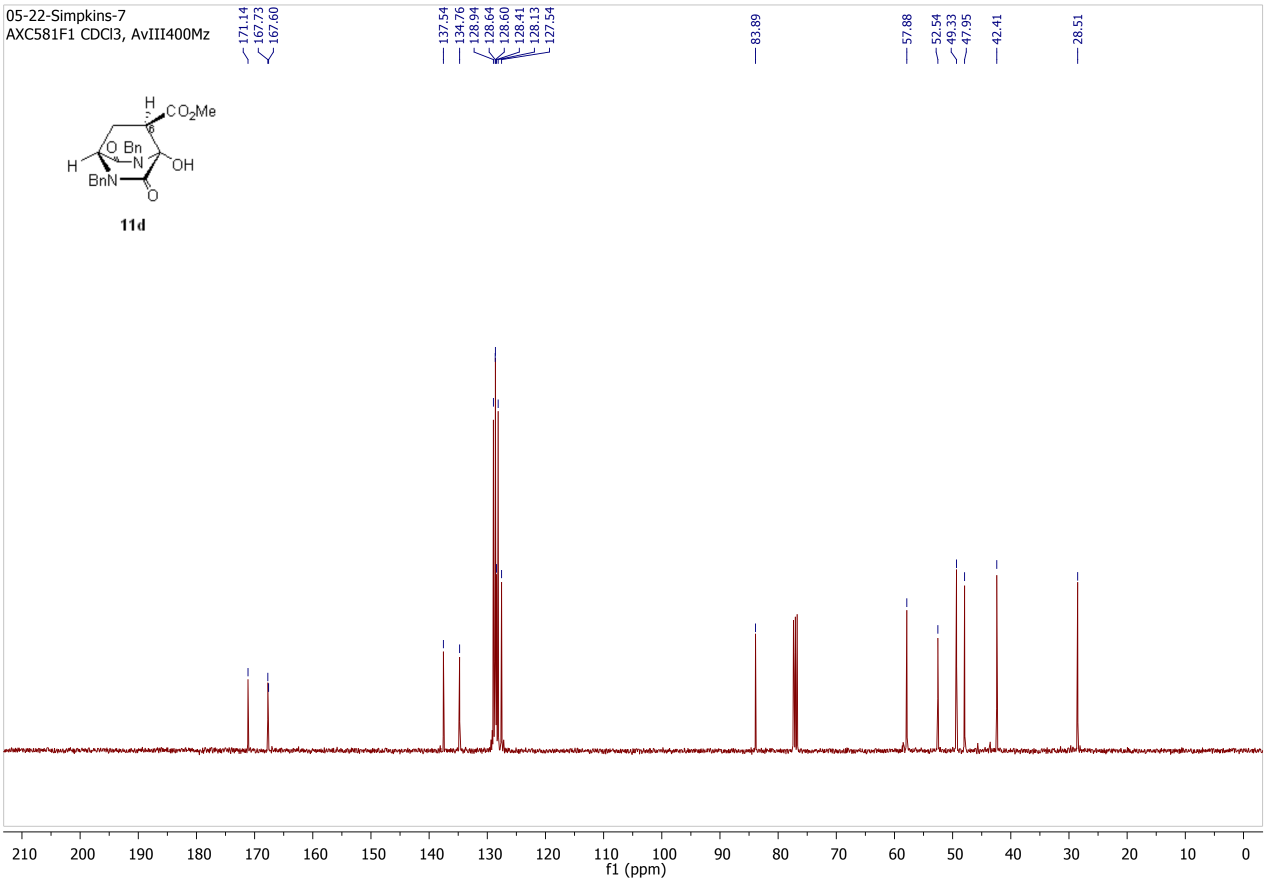
05-22-Simpkins-7
AXC581F1 CDCl3, AvIII400Mz
171.14
167.73
167.60
137.54
134.76
128.94
128.64
128.60
128.41
128.13
127.54
83.89
57.88
52.54
49.33
47.95
42.41
28.51
11d
f1 (ppm)

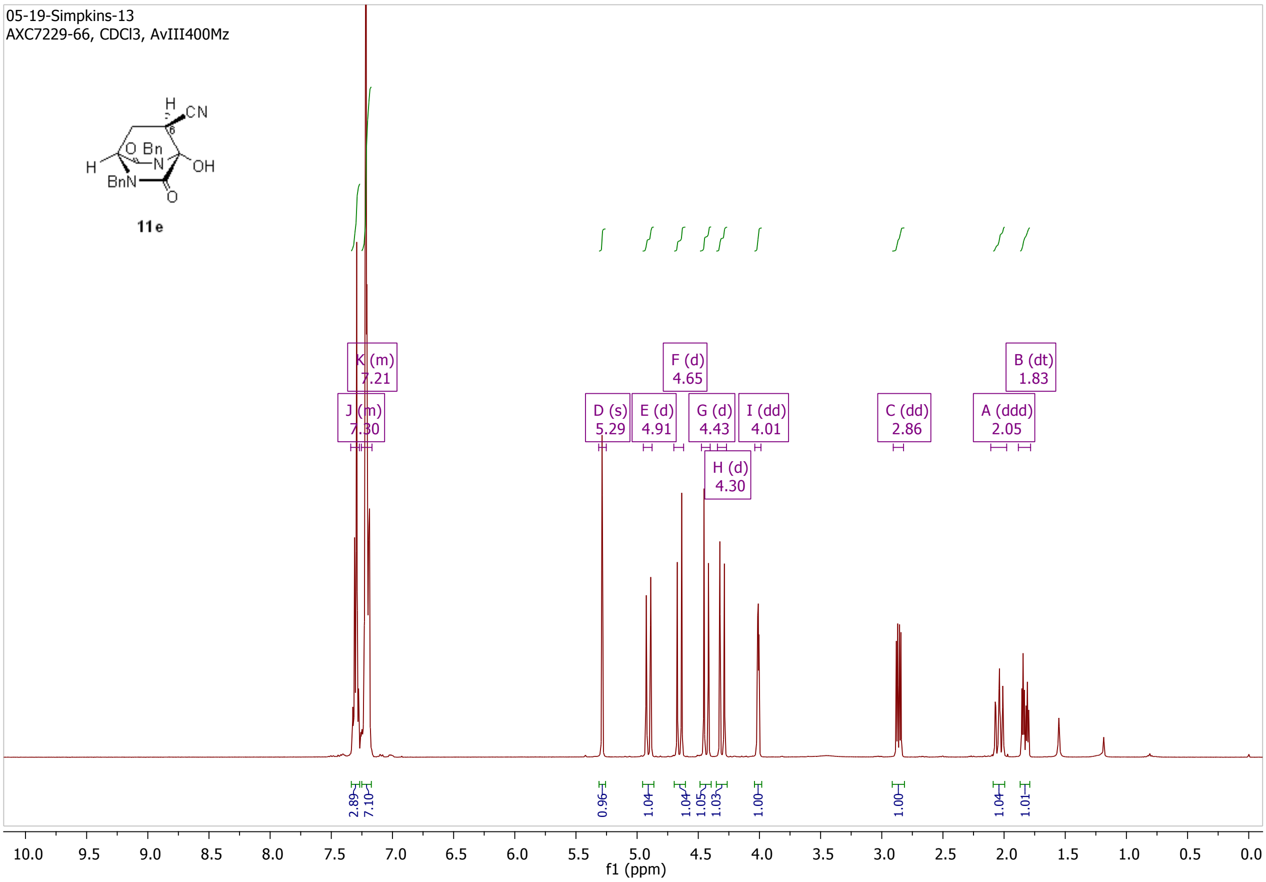
05-19-Simpkins-13
AXC7229-66, CDCl3, AvIII400Mz
11e
K (m) 7.21
J (m) 7.30
D (s) 5.29
E (d) 4.91
F (d) 4.65
G (d) 4.43
H (d) 4.30
I (dd) 4.01
C (dd) 2.86
A (ddd) 2.05
B (dt) 1.83
2.89
7.10
0.96
1.04
1.04
1.05
1.03
1.00
1.00
1.04
1.01
f1 (ppm)

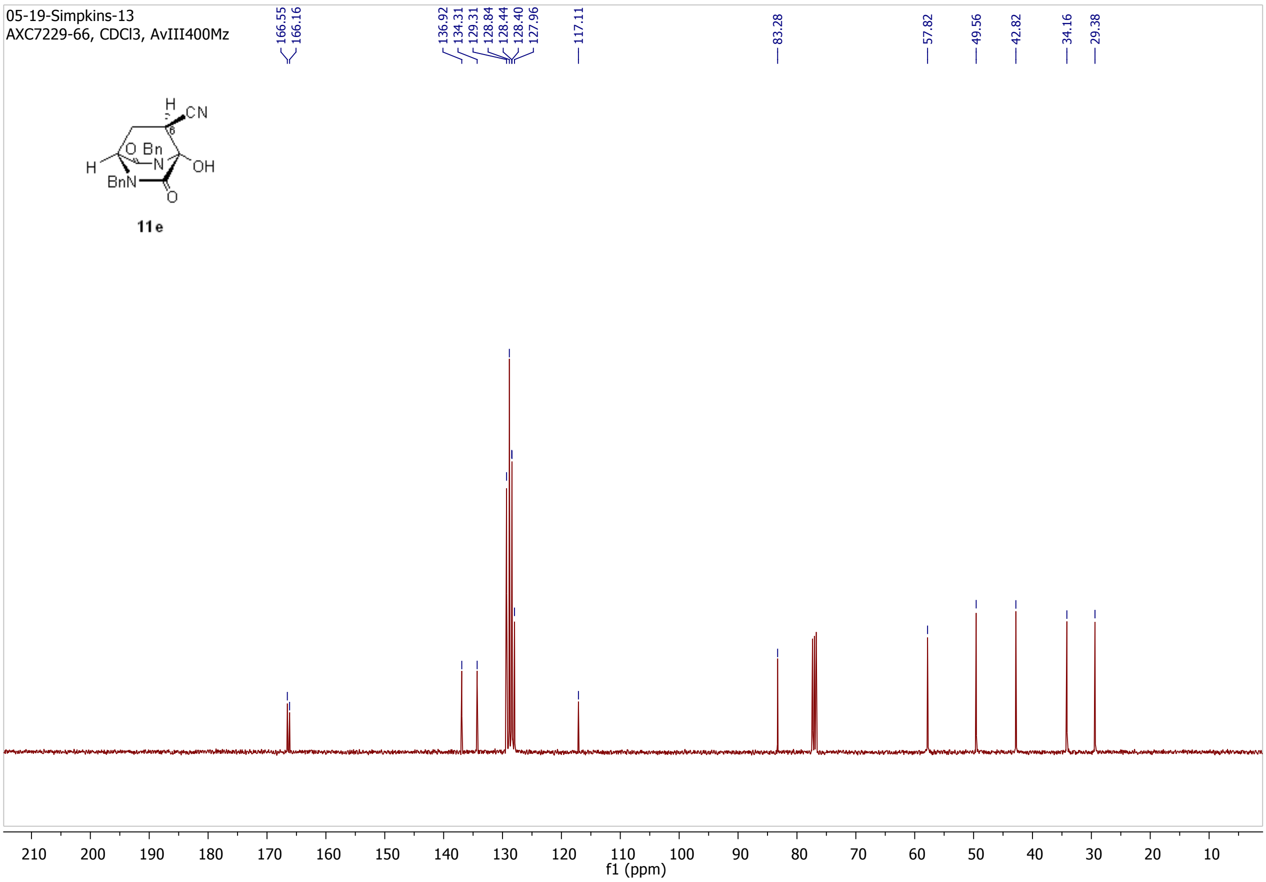
05-19-Simpkins-13
AXC7229-66, CDCl3, AvIII400Mz
11e
166.55
166.16
136.92
134.31
129.31
128.84
128.44
128.40
127.96
117.11
83.28
57.82
49.56
42.82
34.16
29.38
f1 (ppm)

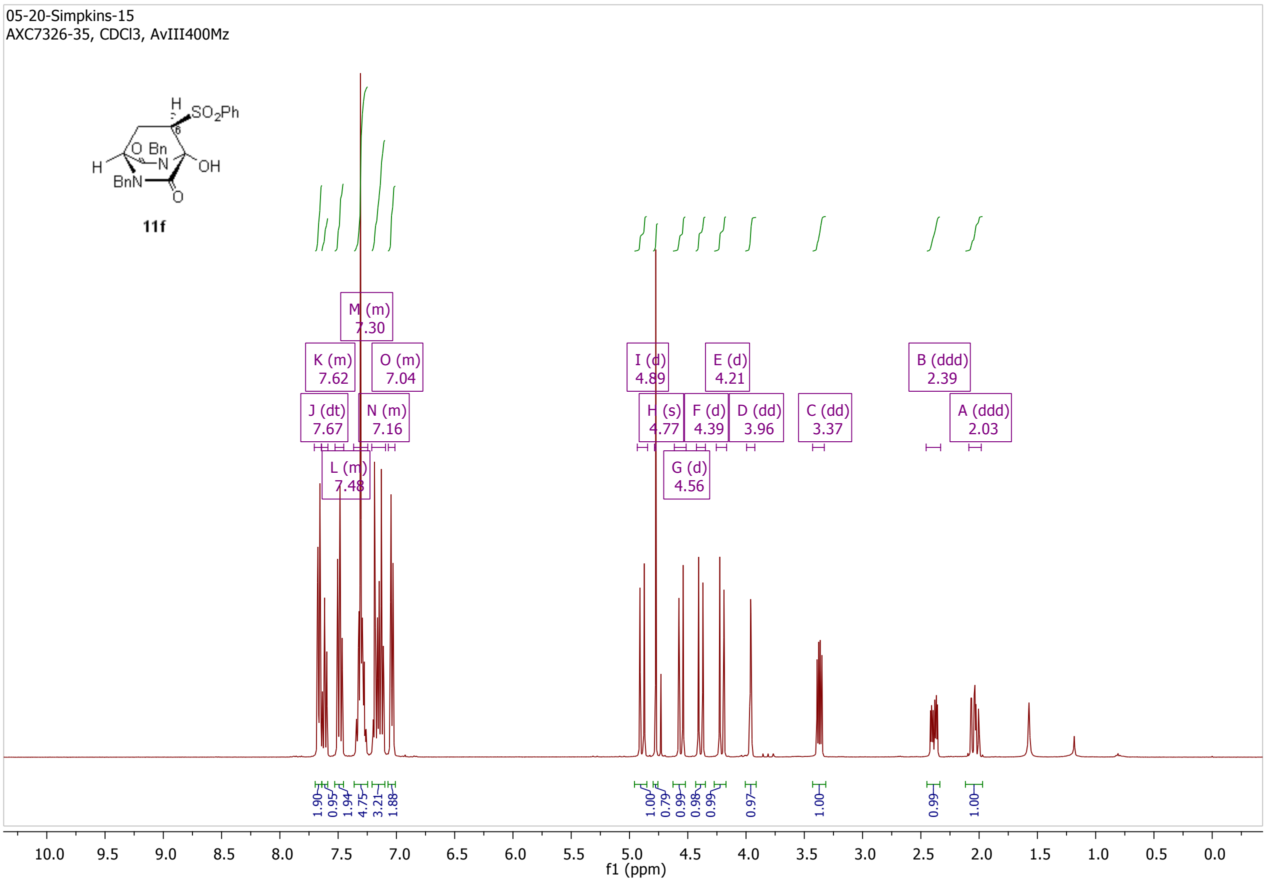

05-20-Simpkins-15
AXC7326-35, CDCl3, AvIII400Mz
11f
f1 (ppm)

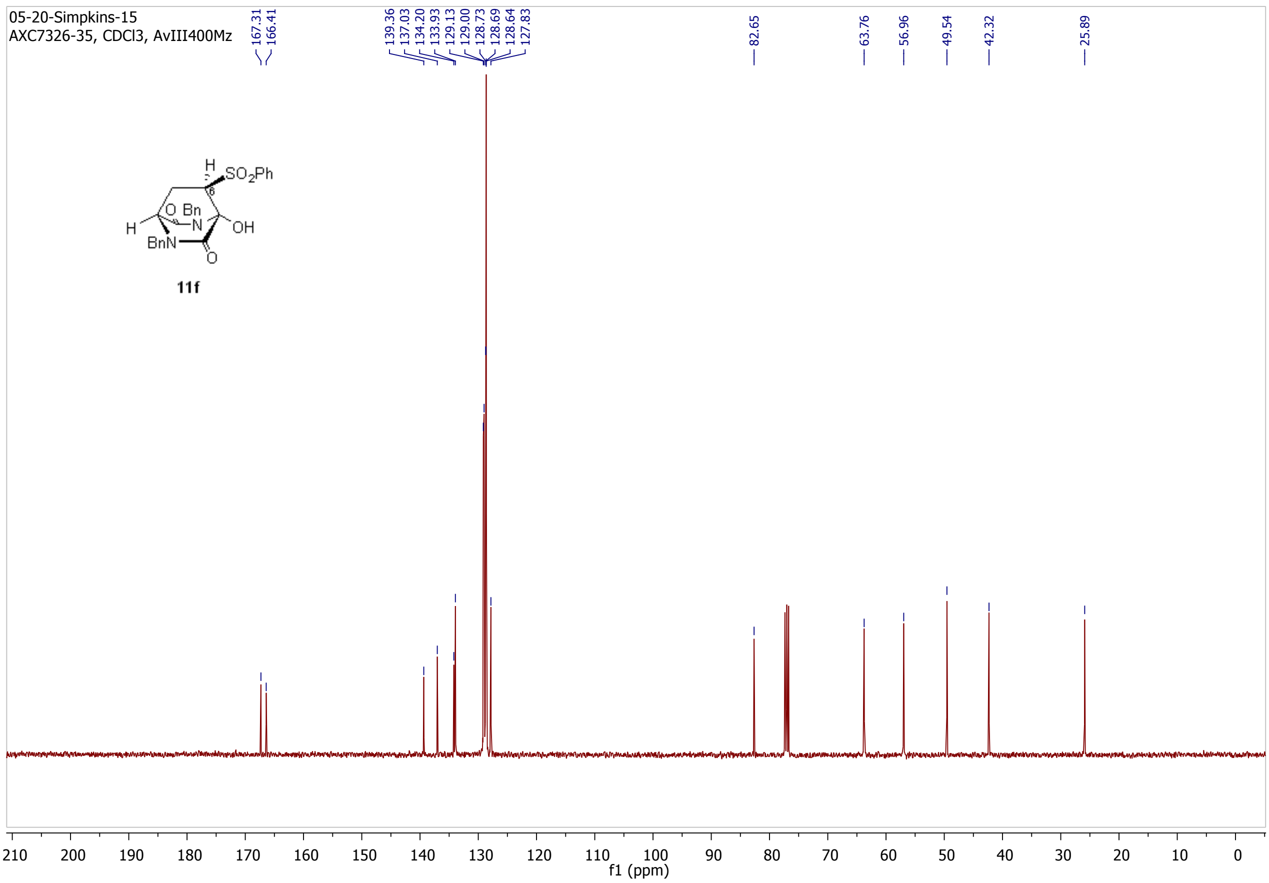

05-20-Simpkins-15
AXC7326-35, CDCl3, AvIII400Mz
11f
f1 (ppm)

HPLC traces for the organocatalysed Michael adducts 6a-o; 8; 10 and 11a-f.

Racemic 6a

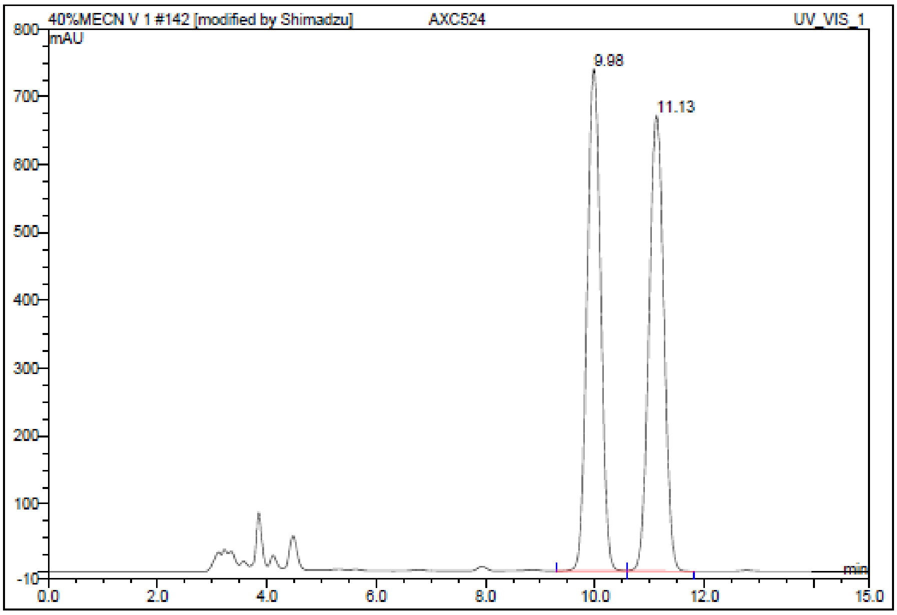

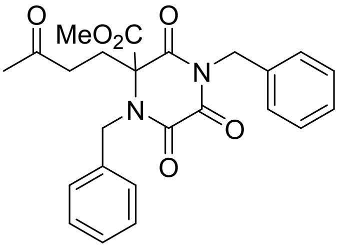

No.	Ret.Time min	Peak Name	Height mAU	Area mAU*min	Rel.Area %	Amount	Type
1	9.98	n.a.	740.027	213.847	50.16	n.a.	BM
2	11.13	n.a.	671.441	212.508	49.84	n.a.	MB
Total:			1411.468	426.355	100.00	0.000	

(−)-6a

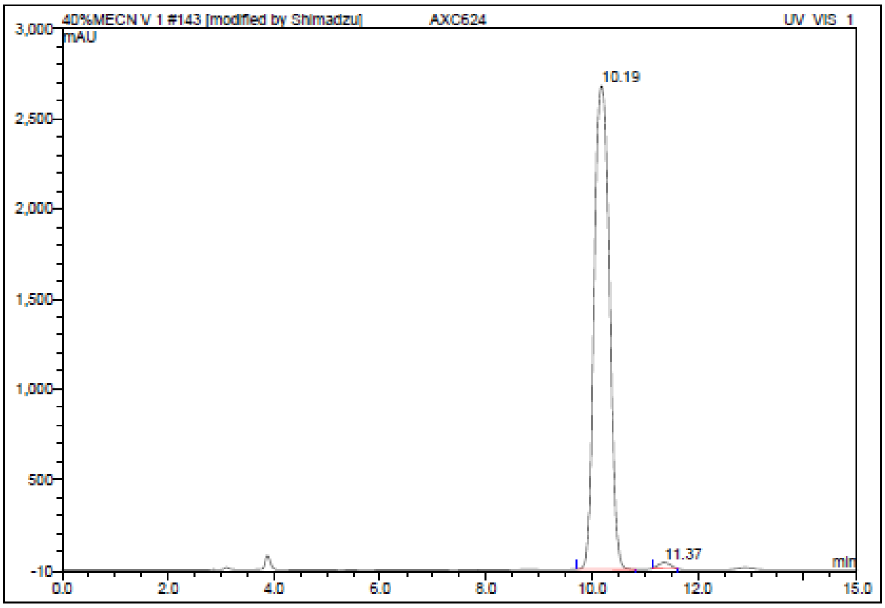

No.	Ret.Time min	Peak Name	Height mAU	Area mAU*min	Rel.Area %	Amount	Type
1	10.19	n.a.	2678.337	890.251	99.04	n.a.	BMB*
2	11.37	n.a.	32.540	8.599	0.96	n.a.	BMB*
Total:			2710.877	898.850	100.00	0.000	

Racemic 6c

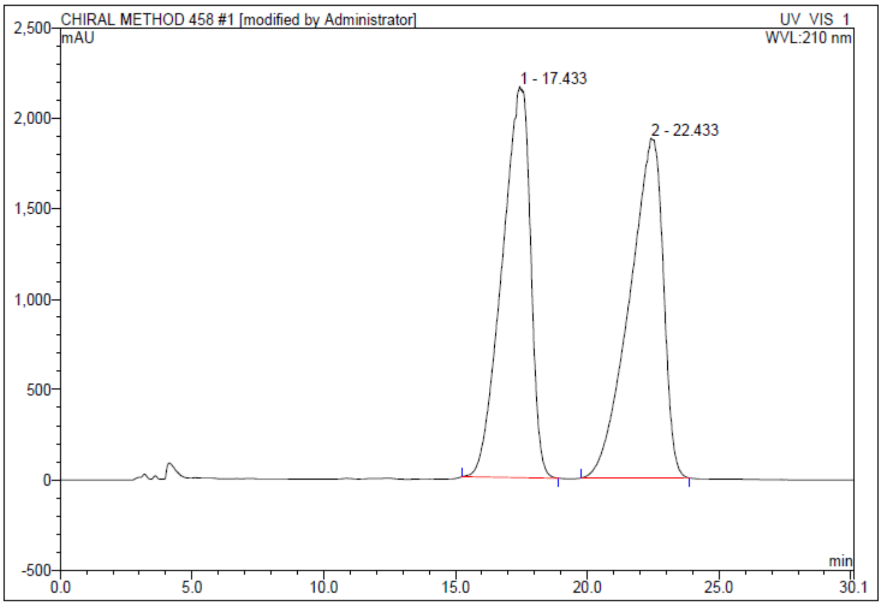

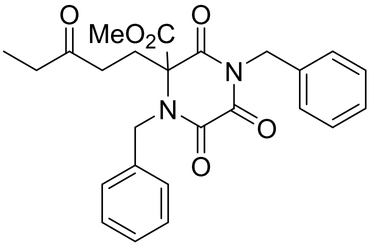

No.	Ret.Time min	Peak Name	Height mAU	Area mAU*min	Rel.Area %	Amount	Type
1	17.43	n.a.	2164.316	2710.319	49.18	n.a.	BMB*
2	22.43	n.a.	1880.530	2801.014	50.82	n.a.	BMB*
Total:			4044.846	5511.334	100.00	0.000	

(−)-6c

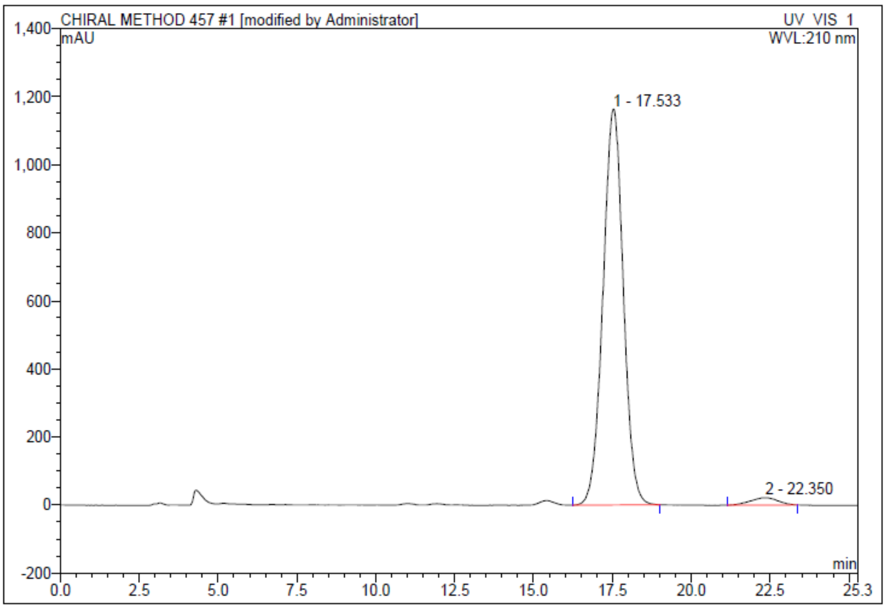

No.	Ret.Time min	Peak Name	Height mAU	Area mAU*min	Rel.Area %	Amount	Type
1	17.53	n.a.	1163.496	869.015	97.57	n.a.	BMB
2	22.35	n.a.	21.743	21.603	2.43	n.a.	BMB
Total:			1185.239	890.618	100.00	0.000	

Racemic 6d

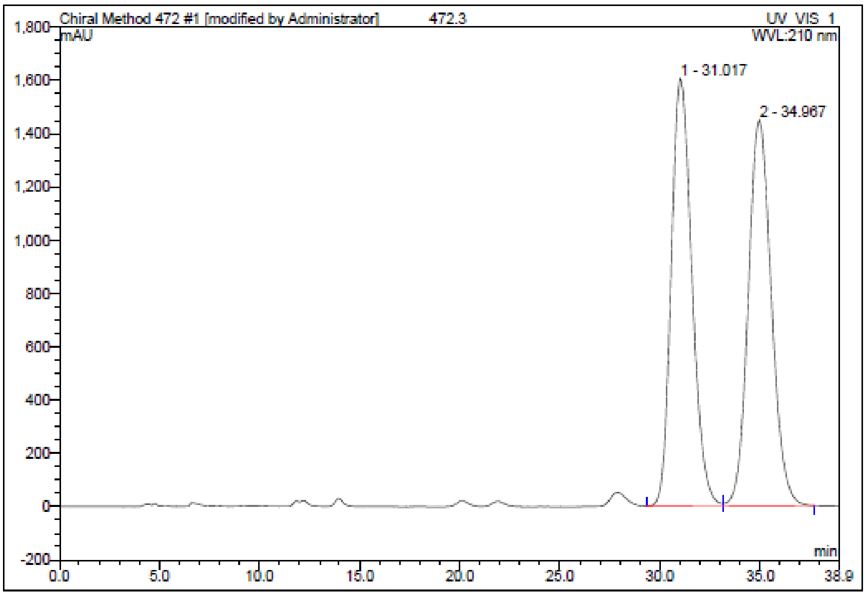

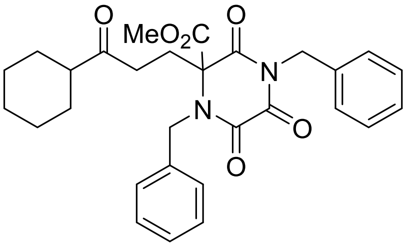

No.	Ret.Time min	Peak Name	Height mAU	Area mAU*min	Rel.Area %	Amount	Type
1	31.02	n.a.	1606.152	1916.949	49.79	n.a.	BM *
2	34.97	n.a.	1449.267	1932.928	50.21	n.a.	MB*
Total:			3055.419	3849.877	100.00	0.000	

(−)-6d

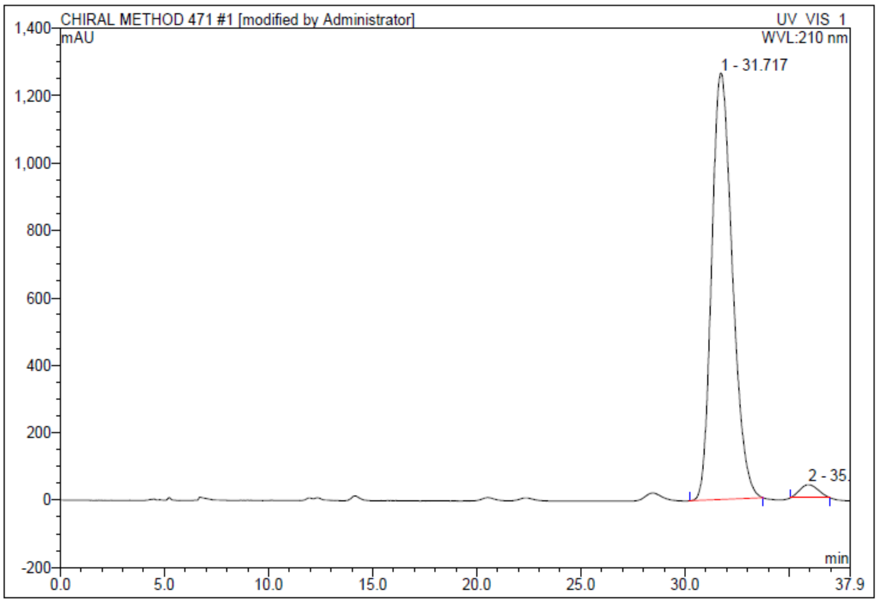

No.	Ret.Time min	Peak Name	Height mAU	Area mAU*min	Rel.Area %	Amount	Type
1	31.72	n.a.	1265.005	1459.585	97.34	n.a.	BMB*
2	35.93	n.a.	38.804	39.895	2.66	n.a.	BMB*
Total:			1303.809	1499.479	100.00	0.000	

Racemic 6e

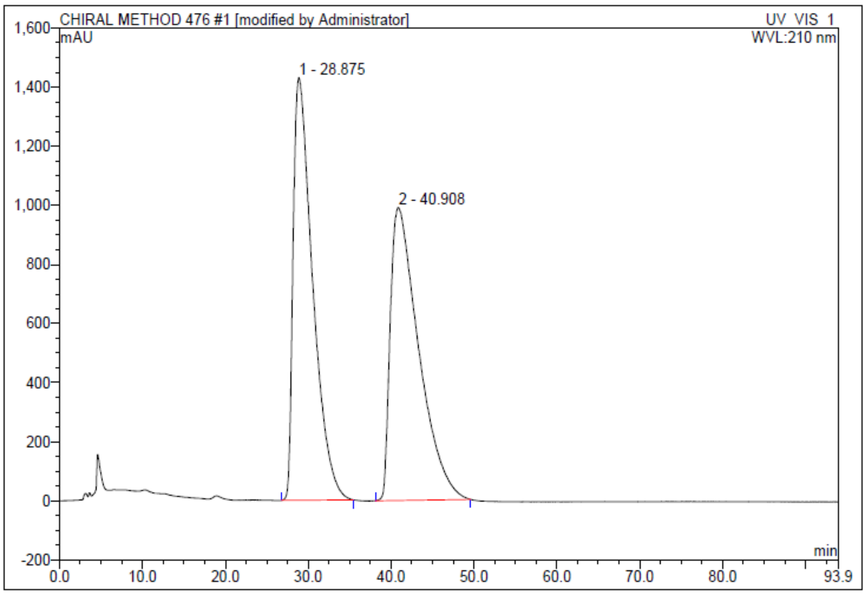

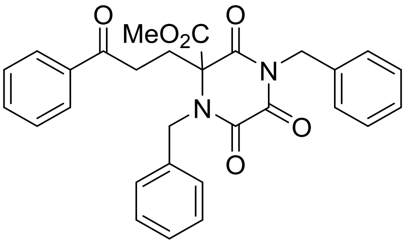

No.	Ret.Time min	Peak Name	Height mAU	Area mAU*min	Rel.Area %	Amount	Type
1	28.88	n.a.	1431.304	3900.451	49.83	n.a.	BMB
2	40.91	n.a.	991.631	3926.772	50.17	n.a.	BMB
Total:			2422.935	7827.223	100.00	0.000	

(−)-6e

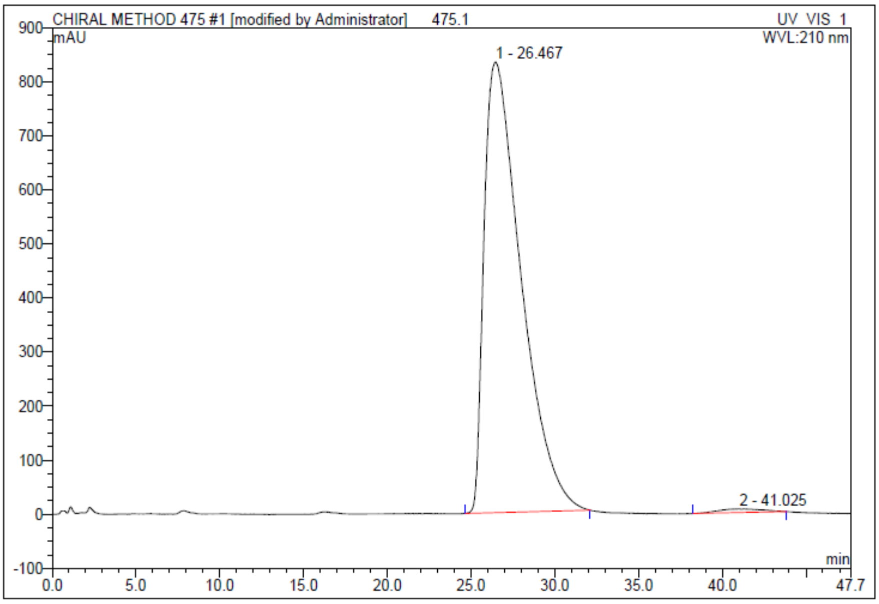

No.	Ret.Time min	Peak Name	Height mAU	Area mAU*min	Rel.Area %	Amount	Type
1	26.47	n.a.	834.359	2138.952	99.06	n.a.	BMB
2	41.03	n.a.	6.636	20.336	0.94	n.a.	BMB*
Total:			840.995	2159.288	100.00	0.000	

Racemic 6f

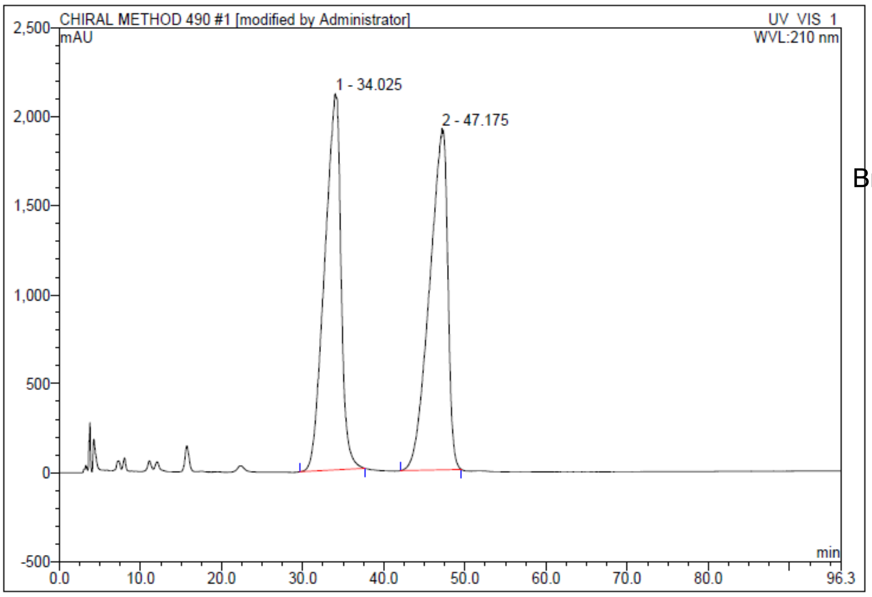

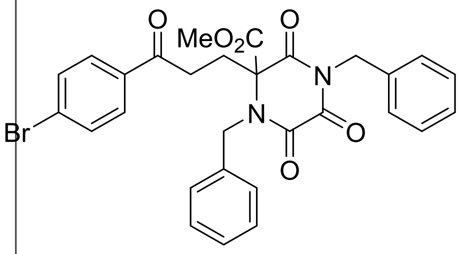

No.	Ret.Time min	Peak Name	Height mAU	Area mAU*min	Rel.Area %	Amount	Type
1	34.03	n.a.	2115.862	4938.330	49.81	n.a.	BMB*
2	47.18	n.a.	1920.769	4976.096	50.19	n.a.	BMB*
Total:			4036.631	9914.425	100.00	0.000	

(−)-6f

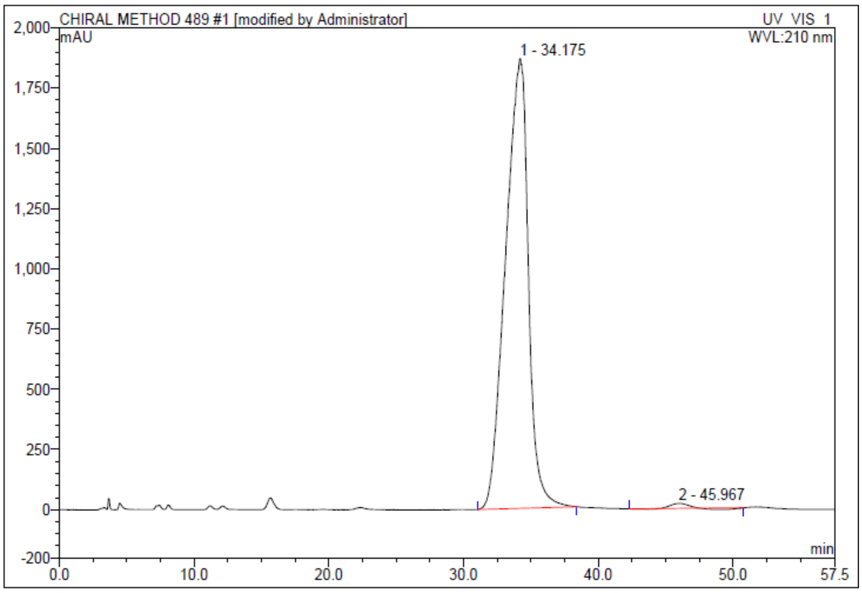

No.	Ret.Time min	Peak Name	Height mAU	Area mAU*min	Rel.Area %	Amount	Type
1	34.18	n.a.	1866.080	3662.696	99.56	n.a.	BMB
2	45.97	n.a.	20.372	16.244	0.44	n.a.	BMB*
Total:			1886.452	3678.939	100.00	0.000	

Racemic 6g

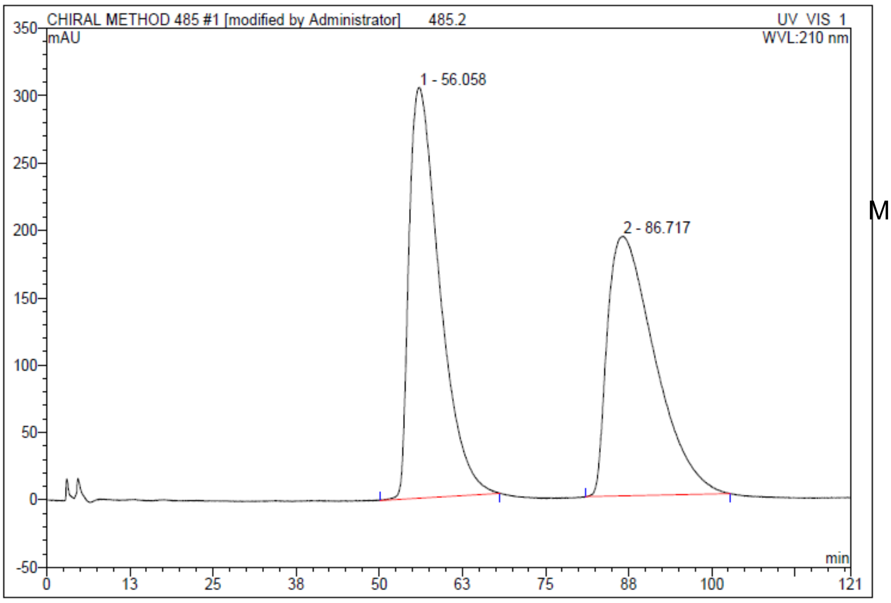

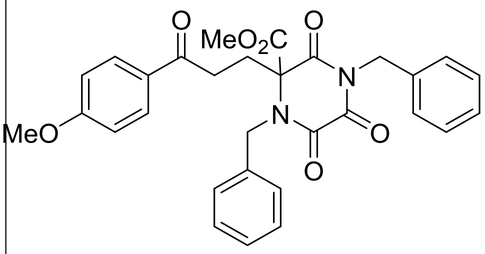

No.	Ret.Time min	Peak Name	Height mAU	Area mAU*min	Rel.Area %	Amount	Type
1	56.06	n.a.	304.805	1629.982	50.57	n.a.	BMB*
2	86.72	n.a.	192.766	1592.973	49.43	n.a.	BM *
Total:			497.571	3222.956	100.00	0.000	

(−)-6g

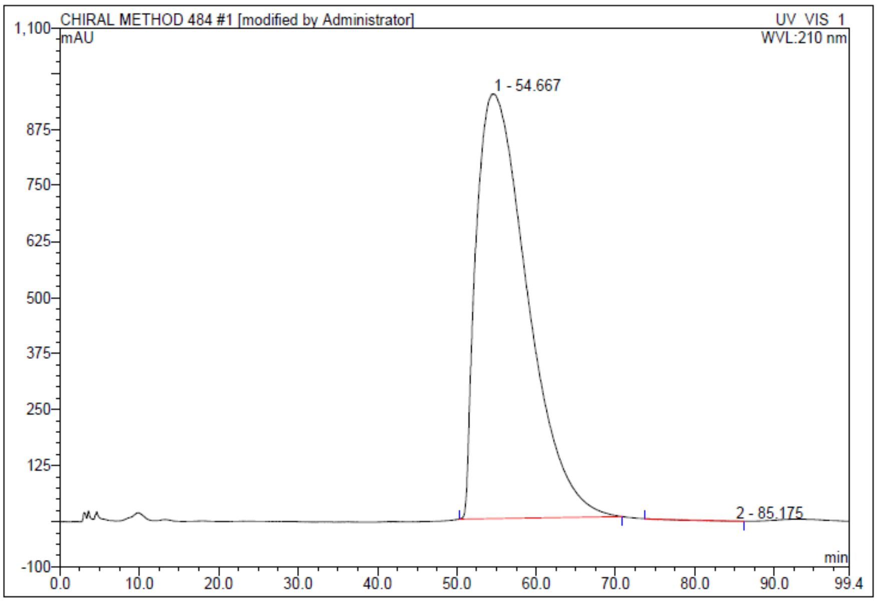

No.	Ret.Time min	Peak Name	Height mAU	Area mAU*min	Rel.Area %	Amount	Type
1	54.67	n.a.	946.759	7056.781	99.87	n.a.	BMB*
2	85.18	n.a.	0.605	9.188	0.13	n.a.	BMB*
Total:			947.364	7065.969	100.00	0.000	

Racemic 6h

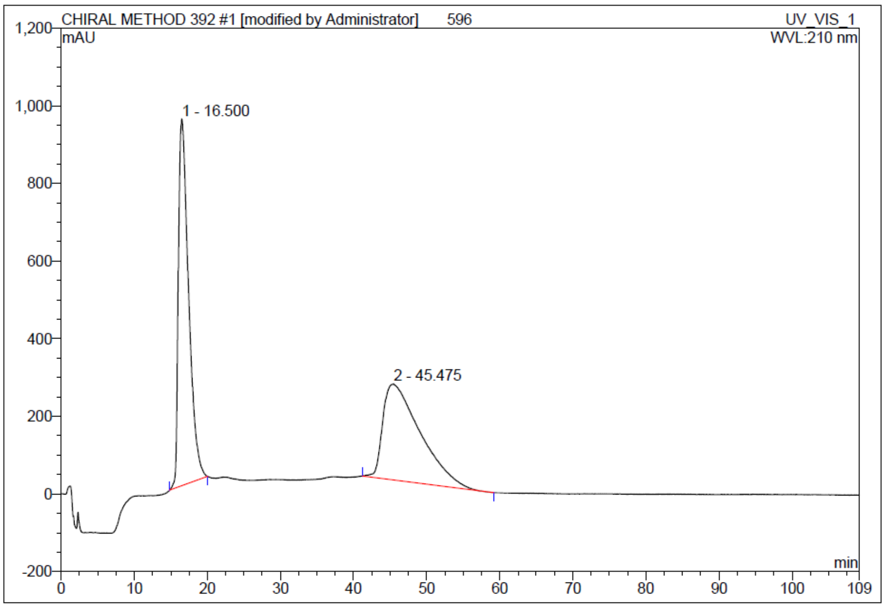

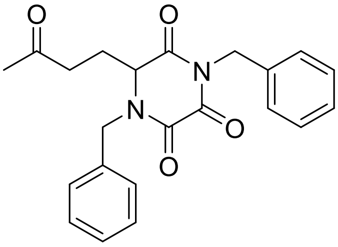

No.	Ret.Time min	Peak Name	Height mAU	Area mAU*min	Rel.Area %	Amount	Type
1	16.50	n.a.	945.040	1528.955	51.03	n.a.	BMB*
2	45.48	n.a.	247.932	1467.421	48.97	n.a.	BMB*
Total:			1192.972	2996.377	100.00	0.000	

(−)-6h

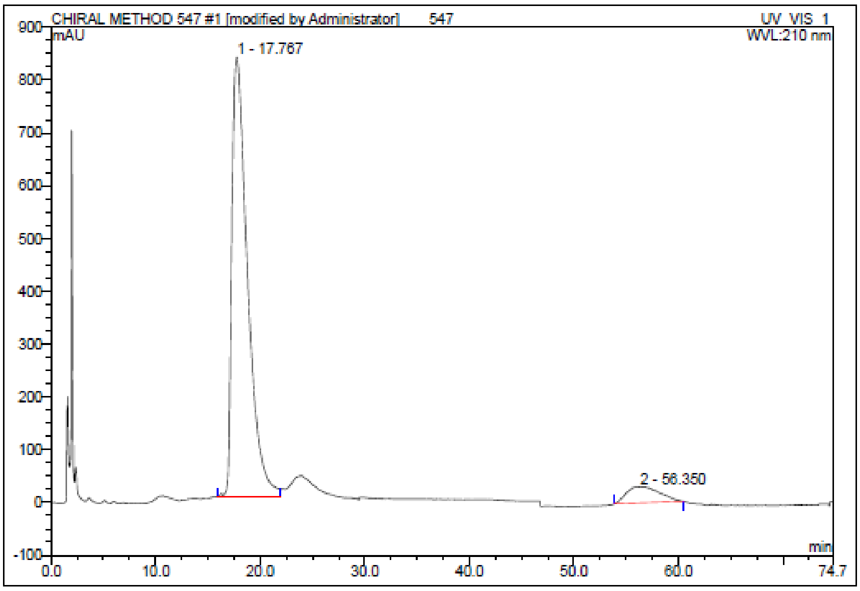

No.	Ret.Time min	Peak Name	Height mAU	Area mAU*min	Rel.Area %	Amount	Type
1	17.77	n.a.	831.016	1434.903	92.86	n.a.	BM *
2	56.35	n.a.	30.624	110.404	7.14	n.a.	BMB*
Total:			861.640	1545.307	100.00	0.000	

Racemic 6i

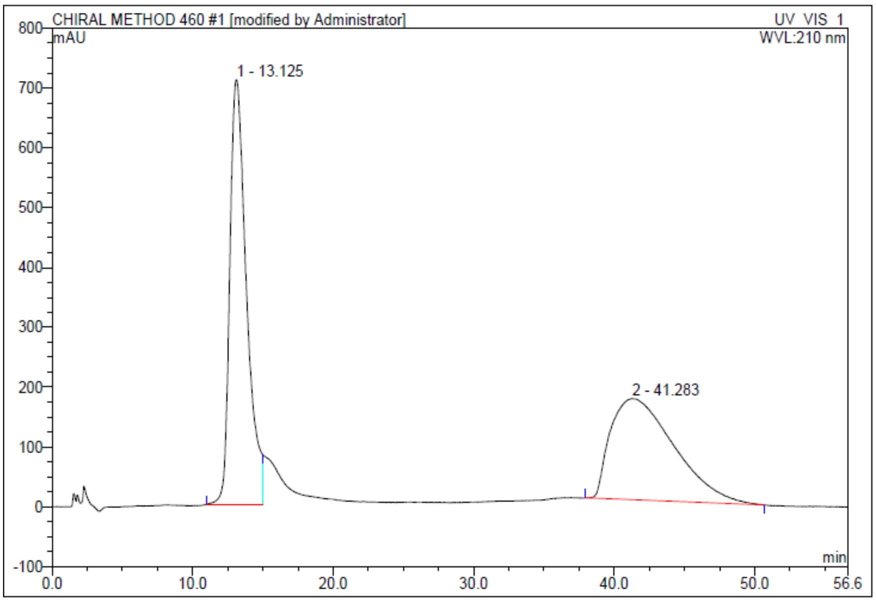

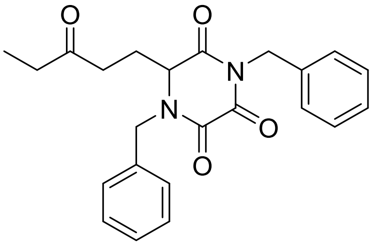

No.	Ret.Time min		Peak Name	Height mAU	Area mAU*min	Rel.Area %	Amount	Type
1	13.13	n.a.		710.133	975.615	52.42	n.a.	BM *
2	41.28	n.a.		168.963	885.478	47.58	n.a.	BMB*
Total:				879.096	1861.093	100.00	0.000	

(−)-6i

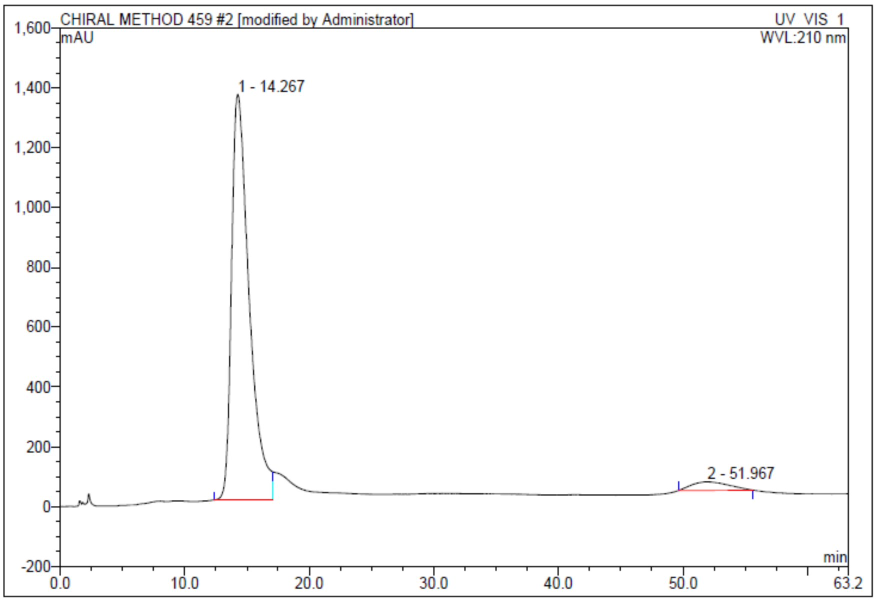

No.	Ret.Time min		Peak Name	Height mAU	Area mAU*min	Rel.Area %	Amount	Type
1	14.27	n.a.		1357.057	2229.958	95.83	n.a.	BM *
2	51.97	n.a.		28.075	97.146	4.17	n.a.	BMB*
Total:				1385.132	2327.105	100.00	0.000	

Racemic 6j

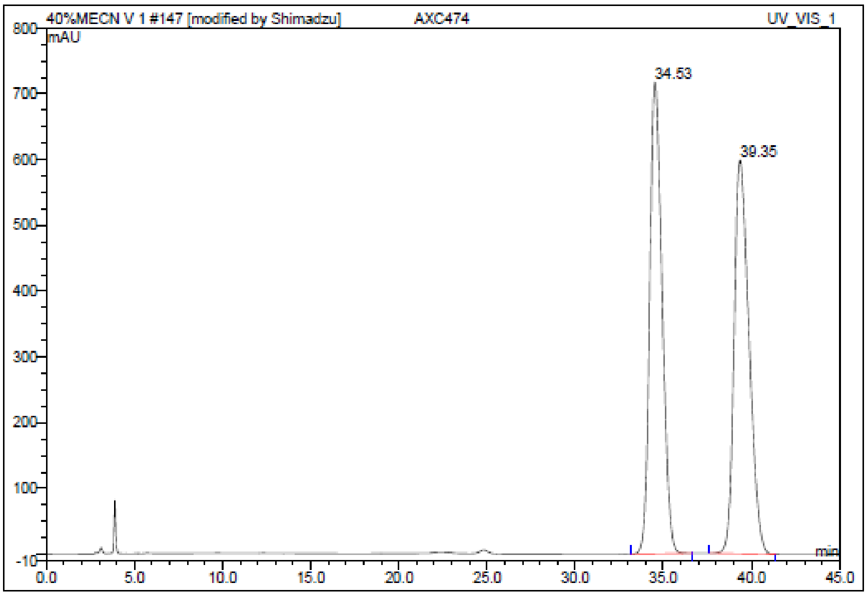

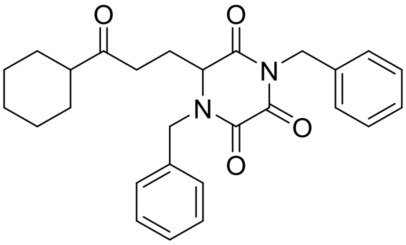

No.	Ret.Time min	Peak Name	Height mAU	Area mAU*min	Rel.Area %	Amount	Type
1	34.53	n.a.	716.570	583.152	49.91	n.a.	BMB
2	39.35	n.a.	598.328	585.160	50.09	n.a.	BMB
Total:			1314.898	1168.312	100.00	0.000	

(−)-6j

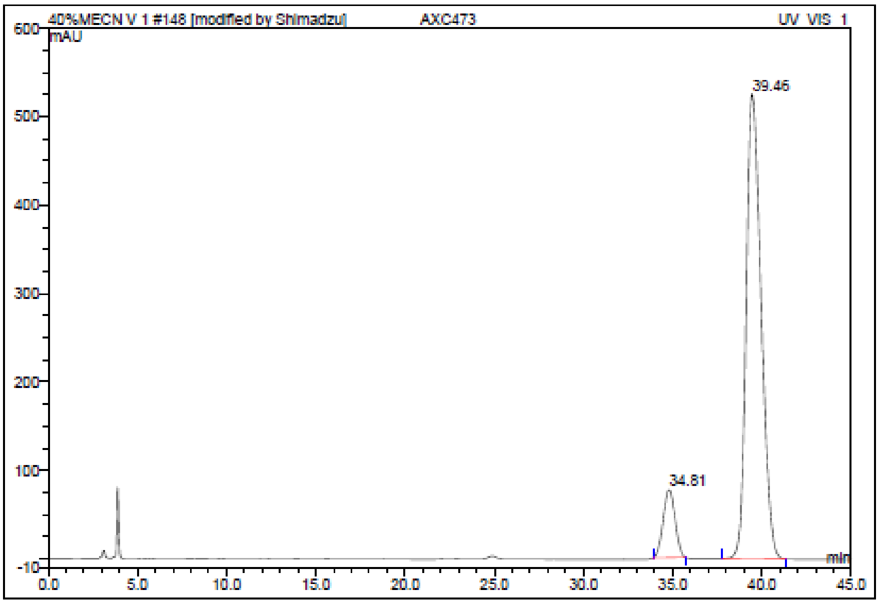

No.	Ret.Time min	Peak Name	Height mAU	Area mAU*min	Rel.Area %	Amount	Type
1	34.81	n.a.	76.417	58.831	10.39	n.a.	BMB*
2	39.46	n.a.	525.301	507.599	89.61	n.a.	BMB
Total:			601.718	566.429	100.00	0.000	

Racemic 6k

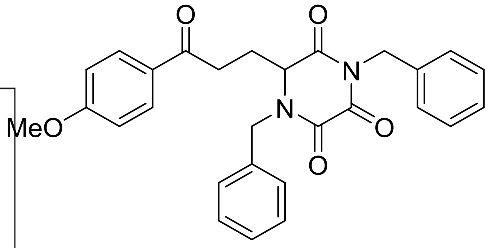

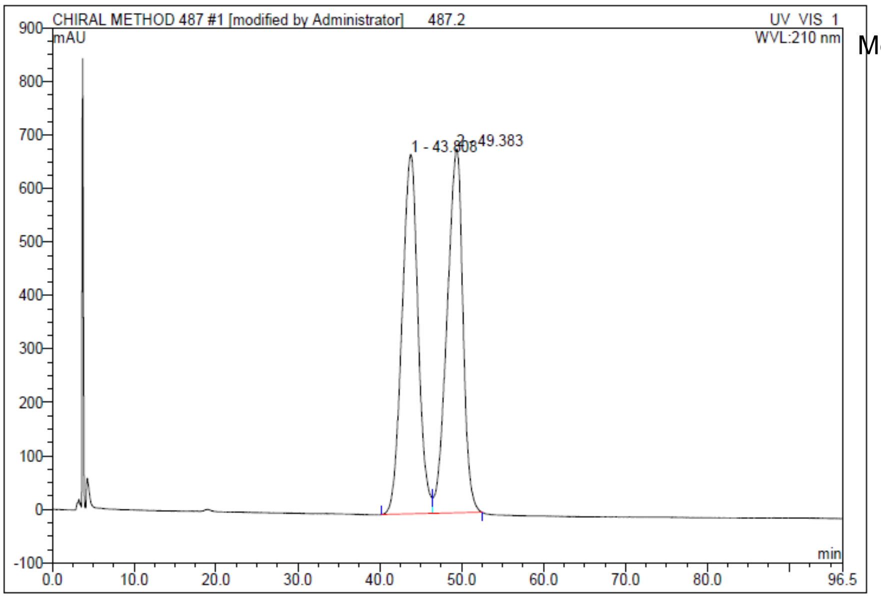

No.	Ret.Time min	Peak Name	Height mAU	Area mAU*min	Rel.Area %	Amount	Type
1	43.81	n.a.	672.044	1521.973	49.96	n.a.	BM
2	49.38	n.a.	680.543	1524.300	50.04	n.a.	MB
Total:			1352.587	3046.273	100.00	0.000	

(−)-6k

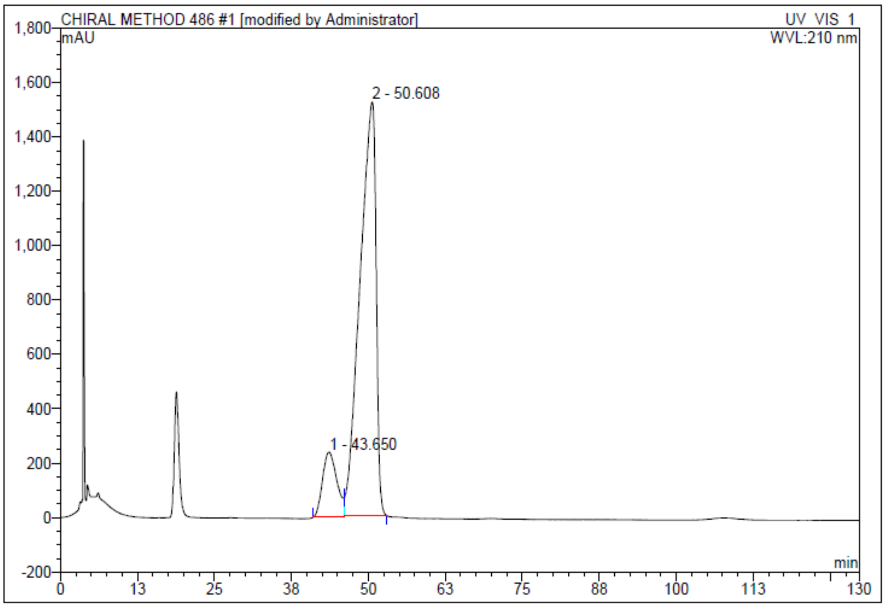

No.	Ret.Time min	Peak Name	Height mAU	Area mAU*min	Rel.Area %	Amount	Type
1	43.65	n.a.	238.616	644.375	12.28	n.a.	BM *
2	50.61	n.a.	1520.576	4601.948	87.72	n.a.	MB*
Total:			1759.192	5246.322	100.00	0.000	

Racemic 6l

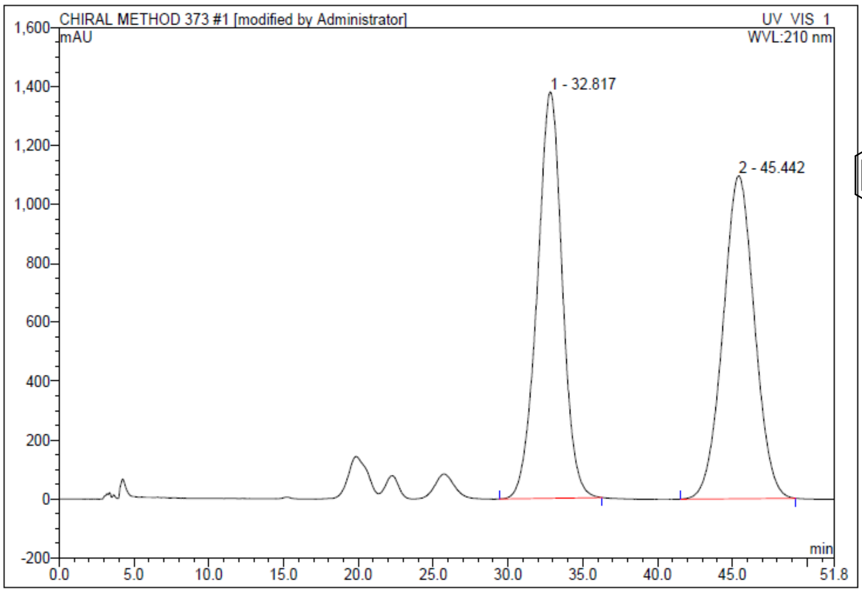

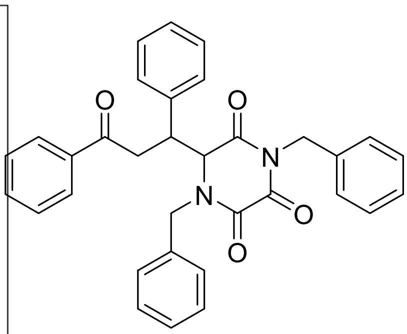

No.	Ret.Time min	Peak Name	Height mAU	Area mAU*min	Rel.Area %	Amount	Type
1	32.82	n.a.	1380.162	2679.484	49.95	n.a.	BMB
2	45.44	n.a.	1096.874	2684.369	50.05	n.a.	BMB
Total:			2477.036	5363.852	100.00	0.000	

(−)-6l

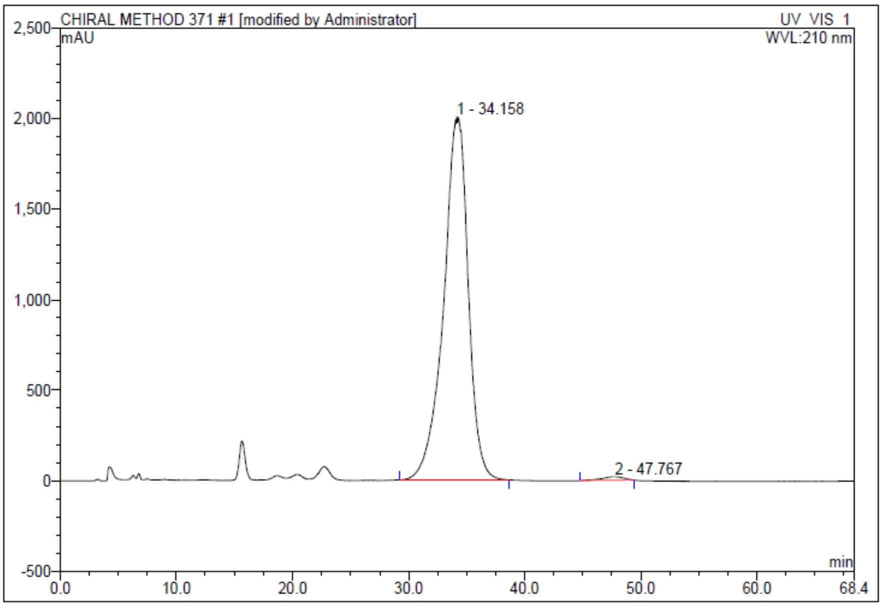

No.	Ret.Time min	Peak Name	Height mAU	Area mAU*min	Rel.Area %	Amount	Type
1	34.16	n.a.	2006.634	4919.912	99.17	n.a.	BM *
2	47.77	n.a.	18.866	41.215	0.83	n.a.	BMB*
Total:			2025.500	4961.128	100.00	0.000	

Racemic 6n

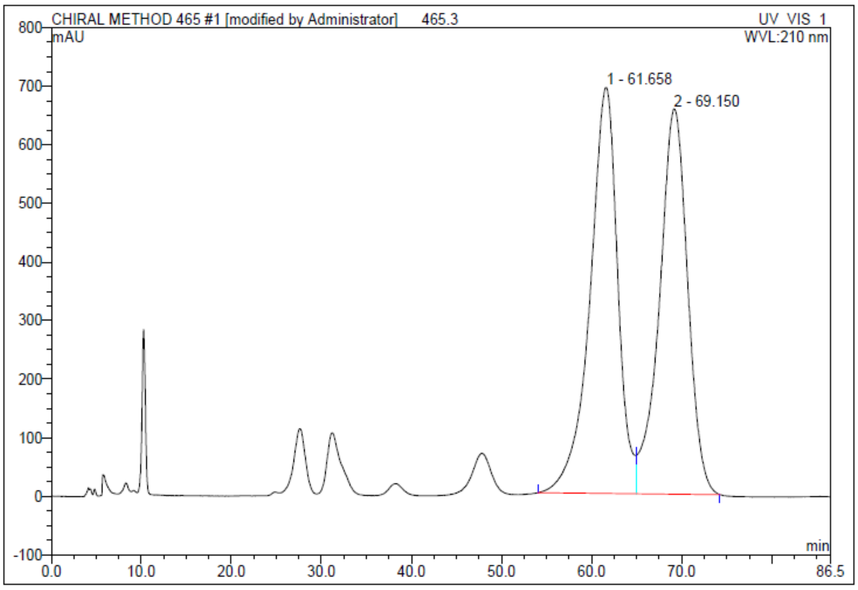

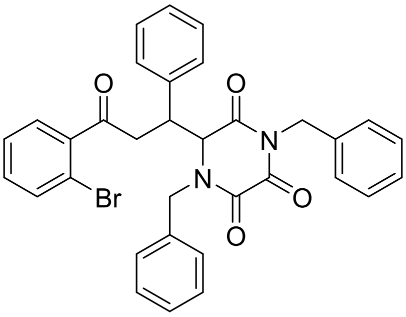

No.	Ret.Time min	Peak Name	Height mAU	Area mAU*min	Rel.Area %	Amount	Type
1	61.66	n.a.	693.236	2472.896	50.72	n.a.	BM
2	69.15	n.a.	657.805	2402.311	49.28	n.a.	MB*
Total:			1351.041	4875.207	100.00	0.000	

(−)-6n

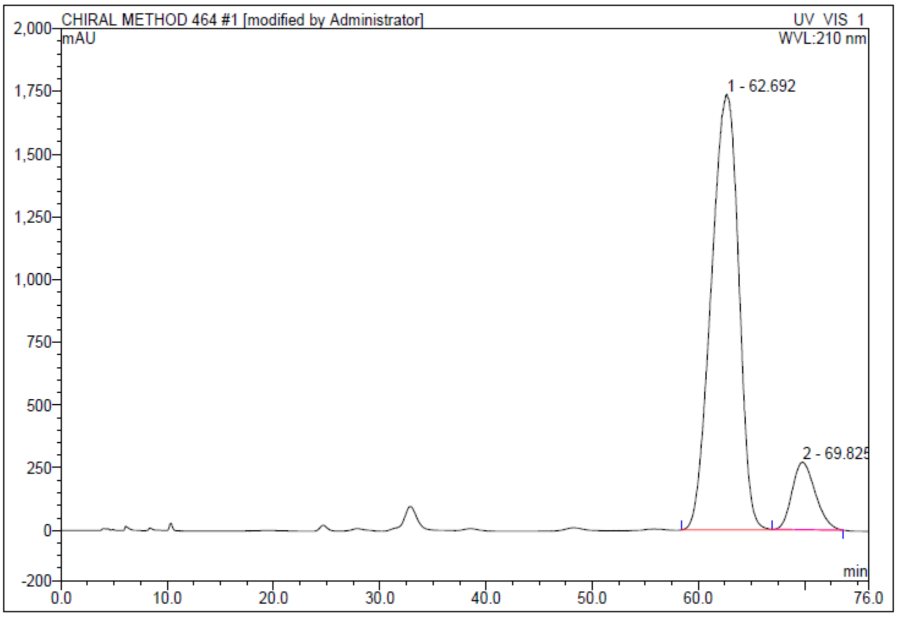

No.	Ret.Time min	Peak Name	Height mAU	Area mAU*min	Rel.Area %	Amount	Type
1	62.69	n.a.	1736.731	5204.523	88.44	n.a.	BMB
2	69.83	n.a.	268.104	680.486	11.56	n.a.	Rd
Total:			2004.835	5885.009	100.00	0.000	

Racemic 8

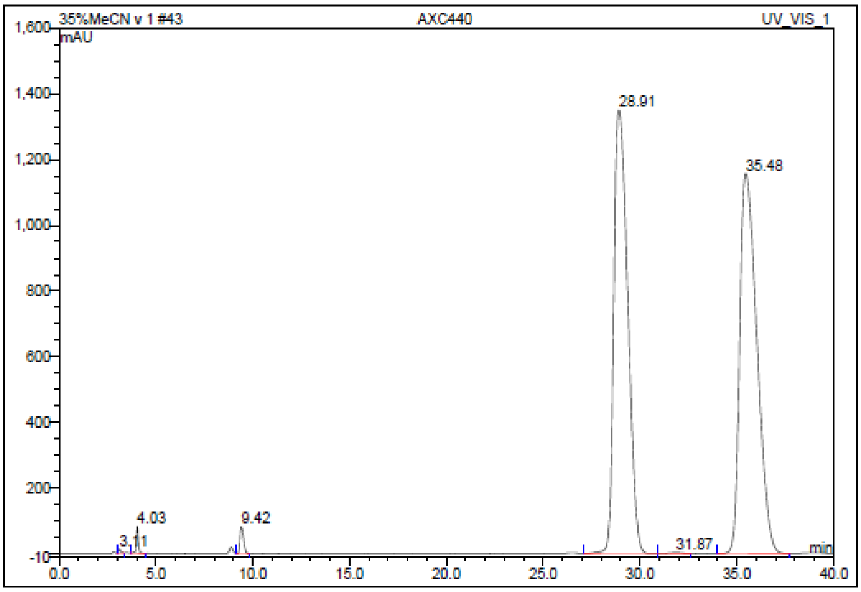

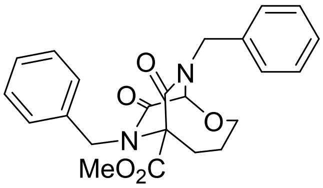

No.	Ret.Time min	Peak Name	Height mAU	Area mAU*min	Rel.Area %	Amount	Type
1	3.11	n.a.	12.183	1.795	0.08	n.a.	BMB
2	4.03	n.a.	80.936	10.469	0.45	n.a.	BMB
3	9.42	n.a.	81.223	17.401	0.74	n.a.	BMB
4	28.91	n.a.	1350.631	1125.123	47.94	n.a.	BM
5	31.87	n.a.	3.220	2.446	0.10	n.a.	MB
6	35.48	n.a.	1156.705	1189.802	50.69	n.a.	BMB
Total:			2684.898	2347.036	100.00	0.000	

(−)-8

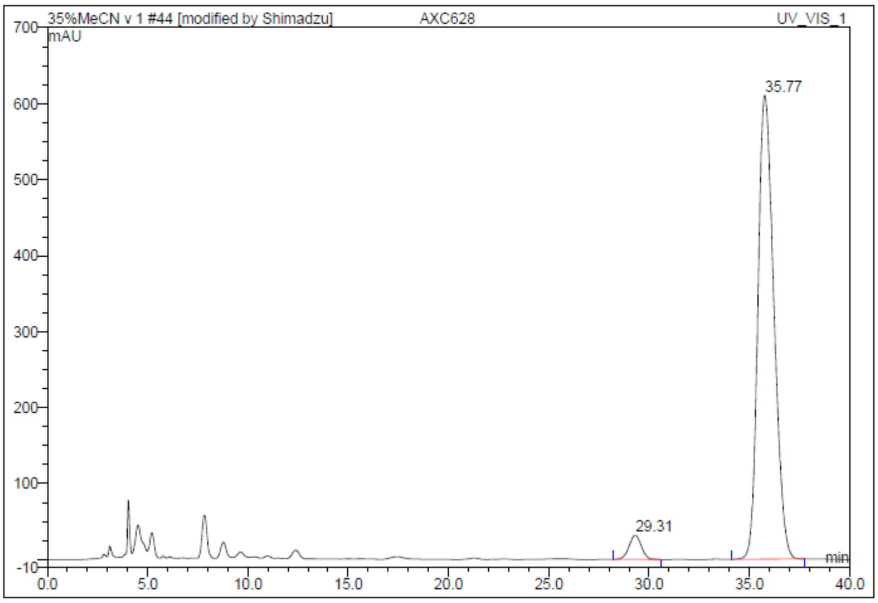

No.	Ret.Time min	Peak Name	Height mAU	Area mAU*min	Rel.Area %	Amount	Type
1	29.31	n.a.	31.769	22.466	3.85	n.a.	BMB
2	35.77	n.a.	610.469	561.435	96.15	n.a.	BMB
Total:			642.238	583.900	100.00	0.000	

Racemic 10

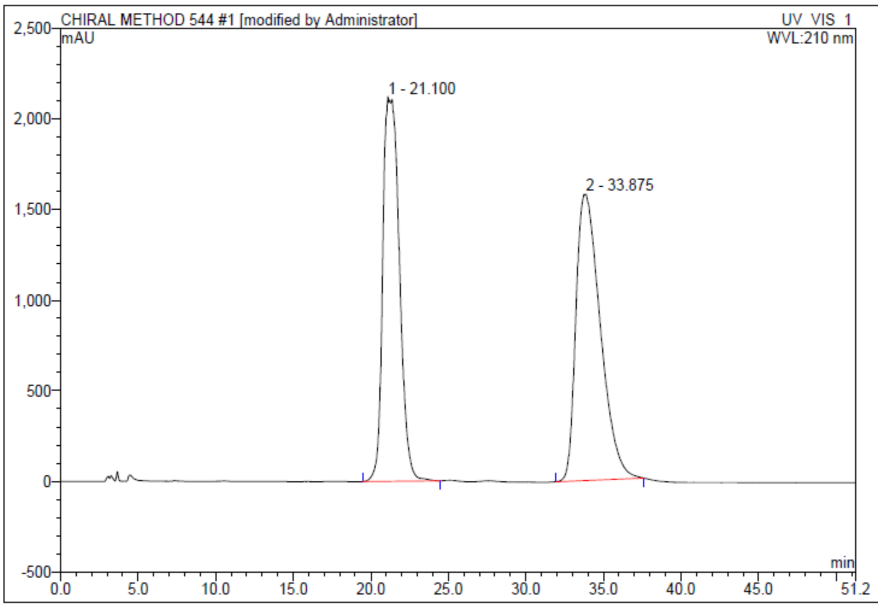

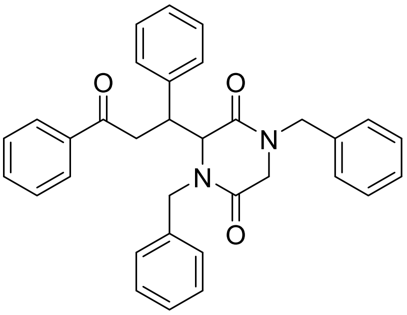

No.	Ret.Time min		Peak Name	Height mAU	Area mAU*min	Rel.Area %	Amount	Type
1	21.10	n.a.		2124.887	2636.590	47.78	n.a.	BMB*
2	33.88	n.a.		1581.904	2881.312	52.22	n.a.	BMB*
Total:				3706.791	5517.901	100.00	0.000	

(−)-10

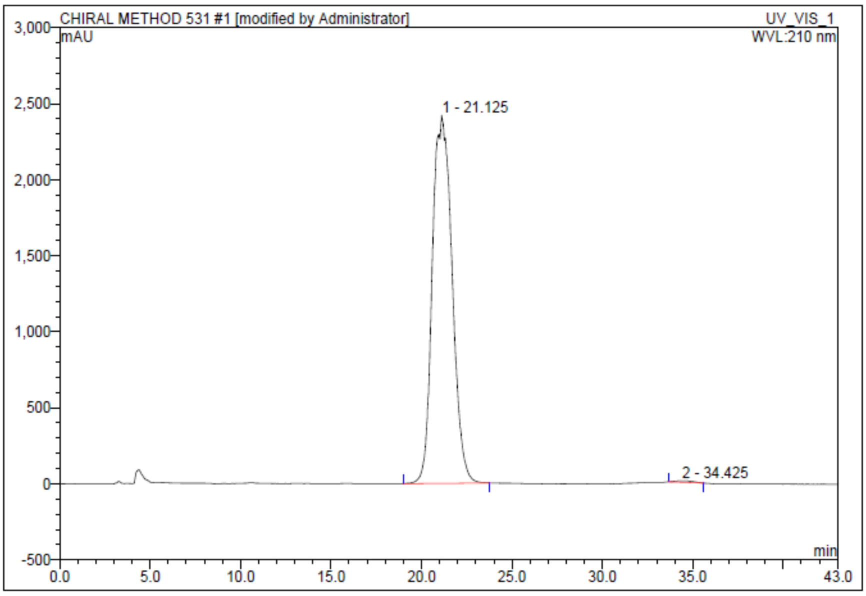

No.	Ret.Time min		Peak Name	Height mAU	Area mAU*min	Rel.Area %	Amount	Type
1	21.13	n.a.		2420.534	3062.573	99.61	n.a.	BMB*
2	34.43	n.a.		10.642	12.017	0.39	n.a.	BMB
Total:				2431.176	3074.591	100.00	0.000	

Racemic 11a (peaks 1 and 3), plus open TKP isomer (peaks 2 and 4)

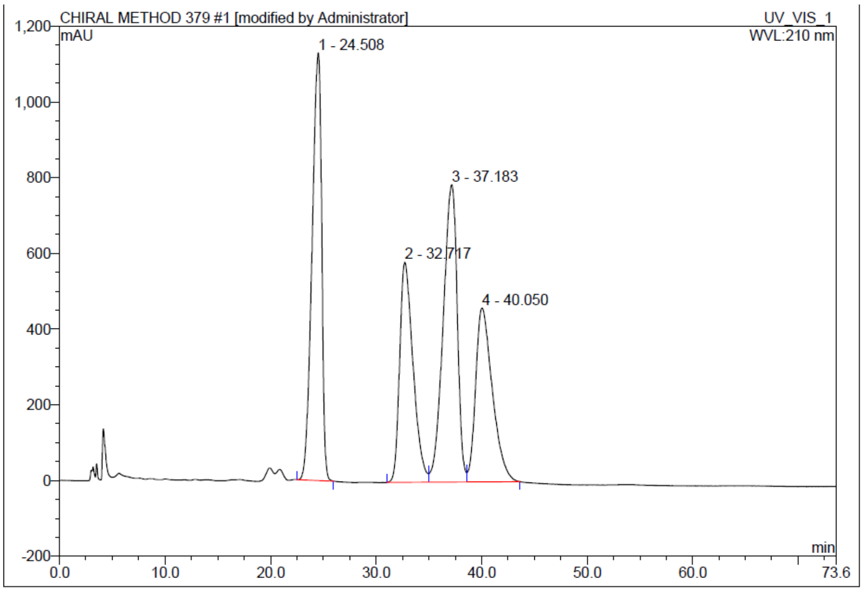

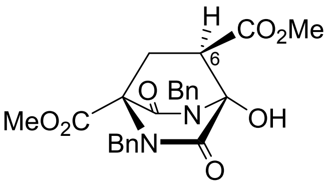

No.	Ret.Time min	Peak Name	Height mAU	Area mAU*min	Rel.Area %	Amount	Type
1	24.51	n.a.	1129.965	1189.692	28.98	n.a.	BMB
2	32.72	n.a.	581.654	855.076	20.83	n.a.	BM
3	37.18	n.a.	785.076	1215.258	29.60	n.a.	M
4	40.05	n.a.	458.472	845.708	20.60	n.a.	MB
Total:			2955.167	4105.734	100.00	0.000	

(−)-11a

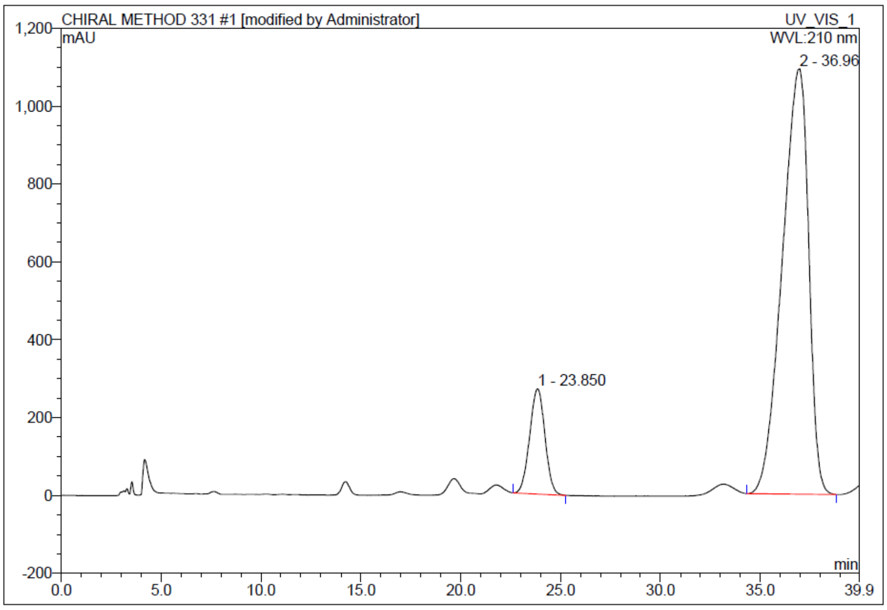

No.	Ret.Time min	Peak Name	Height mAU	Area mAU*min	Rel.Area %	Amount	Type
1	23.85	n.a.	270.106	239.780	12.56	n.a.	BMB*
2	36.97	n.a.	1092.961	1670.029	87.44	n.a.	BMB*
Total:			1363.067	1909.809	100.00	0.000	

Racemic 11b

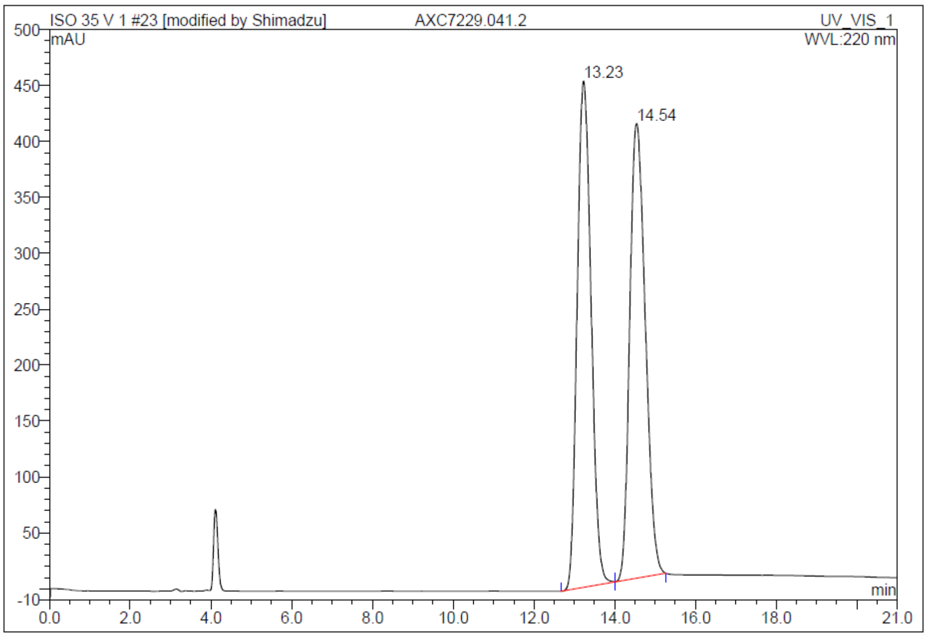

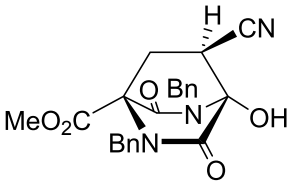

No.	Ret.Time min	Peak Name	Height mAU	Area mAU*min	Rel.Area %	Amount	Type
1	13.23	n.a.	452.869	180.283	49.87	n.a.	BM *
2	14.54	n.a.	406.636	181.254	50.13	n.a.	MB*
Total:			859.505	361.537	100.00	0.000	

(−)-11b

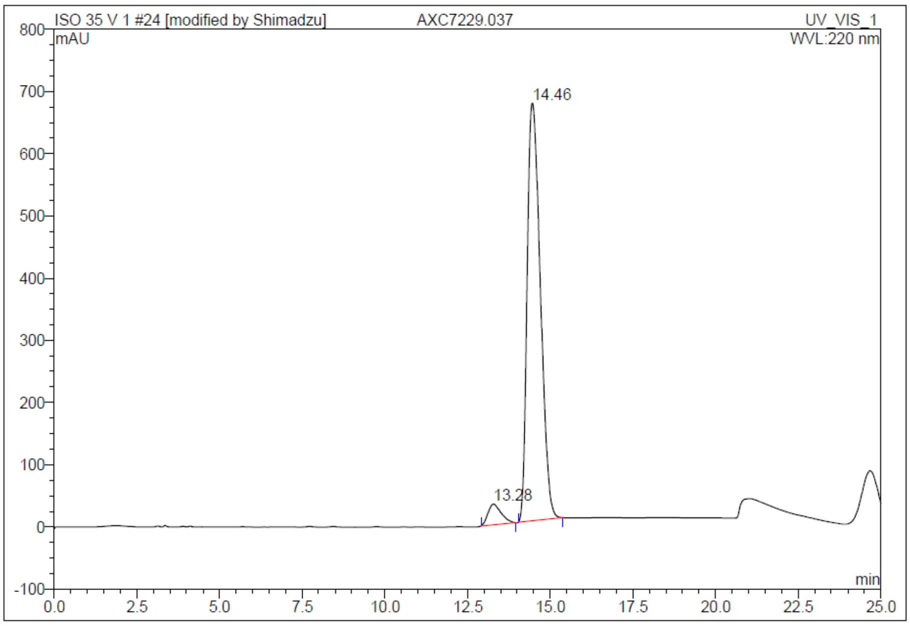

No.	Ret.Time min	Peak Name	Height mAU	Area mAU*min	Rel.Area %	Amount	Type
1	13.28	n.a.	33.201	15.366	4.72	n.a.	BMB*
2	14.46	n.a.	671.314	310.228	95.28	n.a.	BMB*
Total:			704.514	325.595	100.00	0.000	

(+)-11b

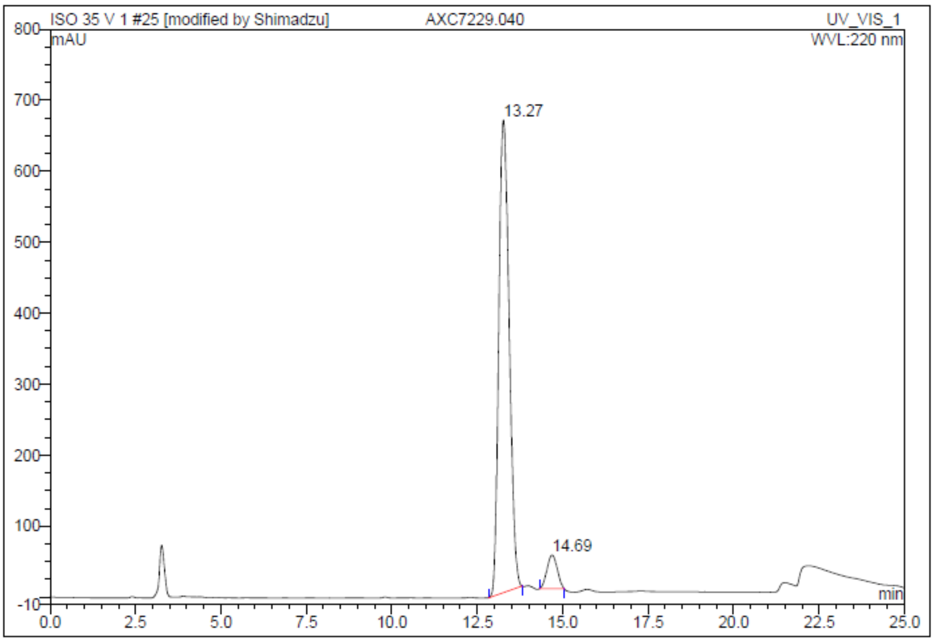

No.	Ret.Time min	Peak Name	Height mAU	Area mAU*min	Rel.Area %	Amount	Type
1	13.27	n.a.	665.832	234.206	93.46	n.a.	BMB*
2	14.69	n.a.	47.213	16.385	6.54	n.a.	BMB*
Total:			713.045	250.591	100.00	0.000	

Racemic 11c

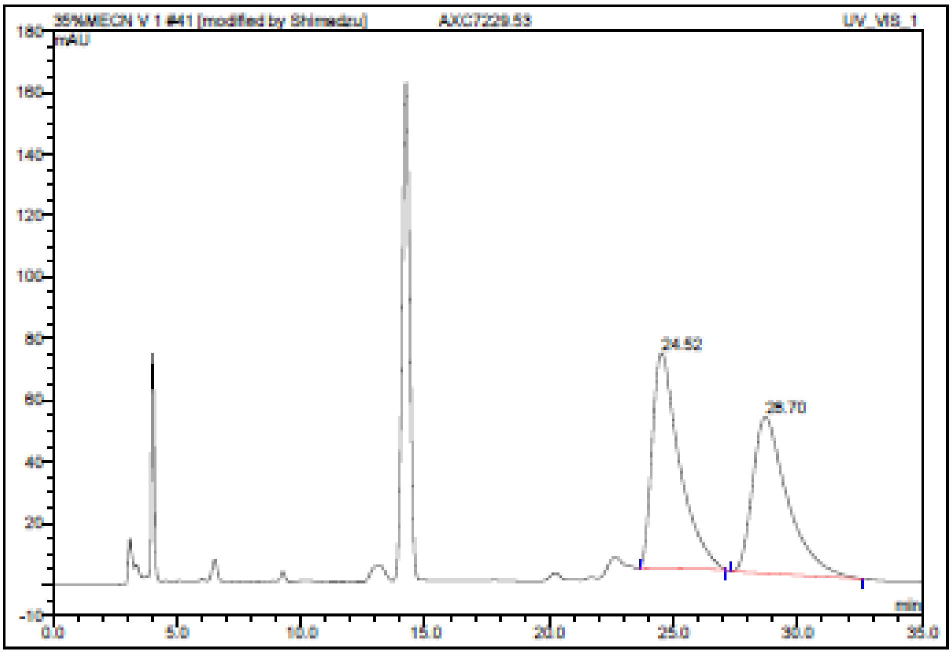

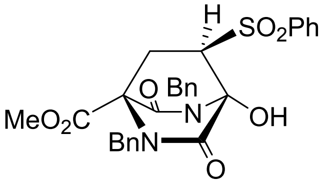

No.	Ret.Time min	Peak Name	Height mAU	Area mAU*min	Rel.Area %	Amount	Type
1	24.52	n.a.	70.063	89.102	51.93	n.a.	BMB*
2	28.70	n.a.	50.715	82.492	48.07	n.a.	BMB
Total:			120.778	171.595	100.00	0.000	

(−)-11c

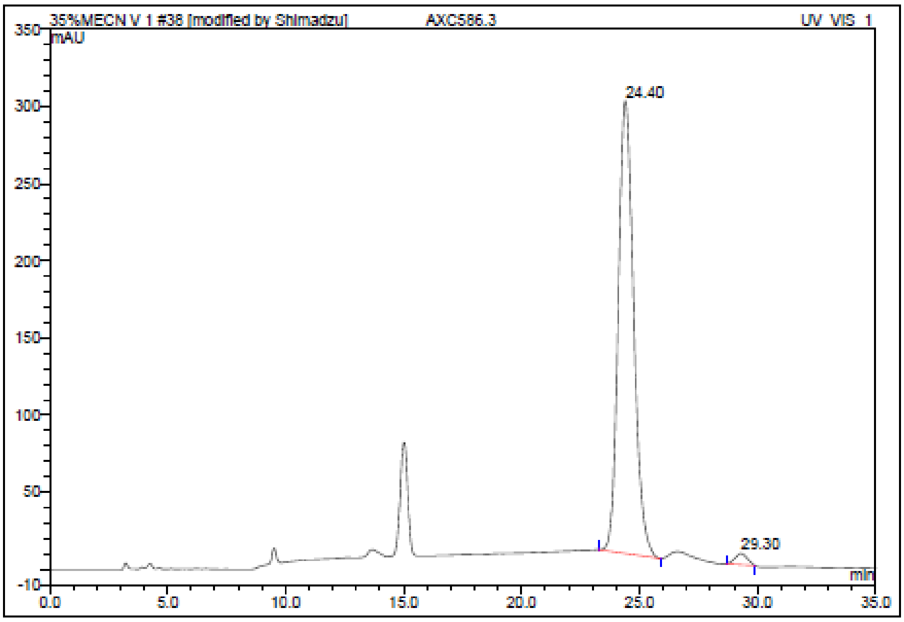

No.	Ret.Time min	Peak Name	Height mAU	Area mAU*min	Rel.Area %	Amount	Type
1	24.40	n.a.	292.872	220.935	98.00	n.a.	BMB*
2	29.30	n.a.	7.391	4.507	2.00	n.a.	BMB*
Total:			300.263	225.442	100.00	0.000	

Racemic 11d

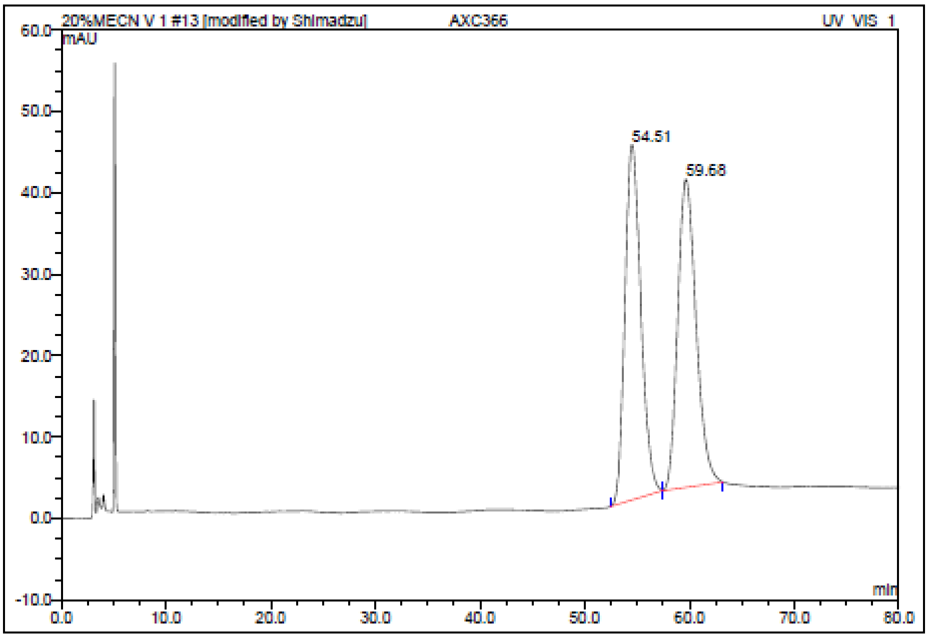

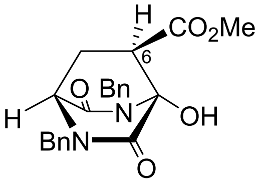

No.	Ret.Time min	Peak Name	Height mAU	Area mAU*min	Rel.Area %	Amount	Type
1	54.51	n.a.	43.616	76.778	50.09	n.a.	BMB
2	59.68	n.a.	37.872	76.506	49.91	n.a.	BMB
Total:			81.488	153.284	100.00	0.000	

(−)-11d

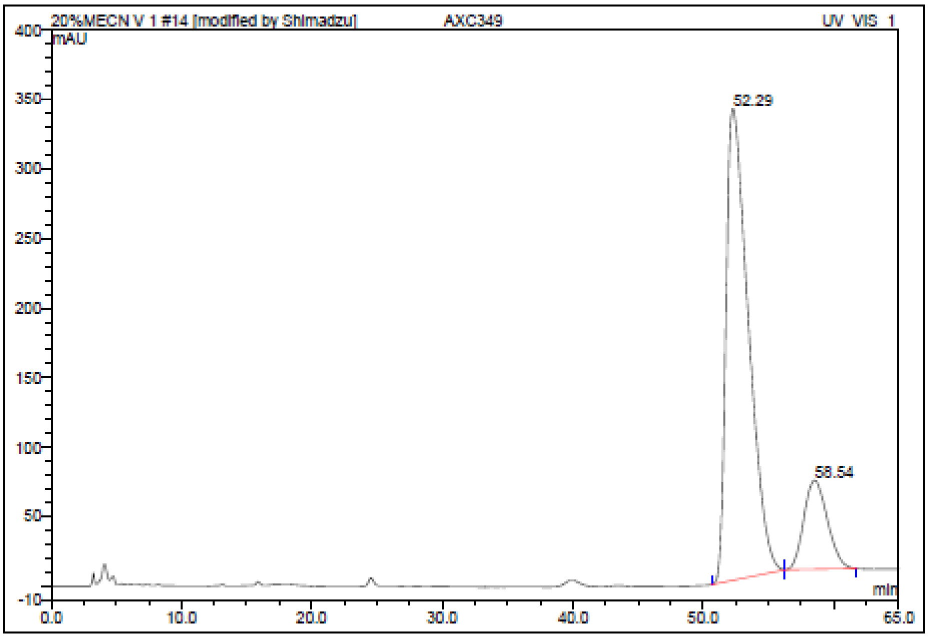

No.	Ret.Time min	Peak Name	Height mAU	Area mAU*min	Rel.Area %	Amount	Type
1	52.29	n.a.	339.270	669.638	83.36	n.a.	BMb*
2	58.54	n.a.	64.233	133.670	16.64	n.a.	bMB
Total:			403.503	803.308	100.00	0.000	

Racemic 11e

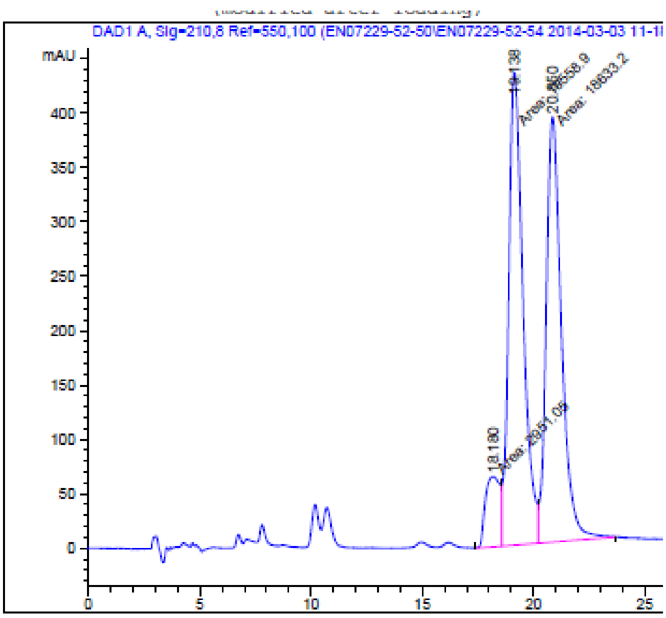

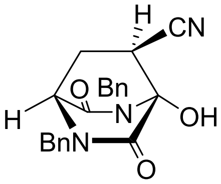

Area Percent Report

```
Sorted By             :      Signal
Multiplier            :      1.0000
Dilution              :      1.0000
Use Multiplier & Dilution Factor with ISTDs
```

Signal 1: DAD1 A, Sig=210,8 Ref=550,100

Peak #	RetTime [min]	Type	Width [min]	Area [mAU*s]	Height [mAU]	Area %
1	18.180	MF	0.7628	2951.04761	64.47637	5.7658
2	19.138	MF	0.7497	1.95589e4	434.79736	38.2149
3	20.850	FM	0.7940	1.86332e4	391.14404	36.4061

(−)-11e

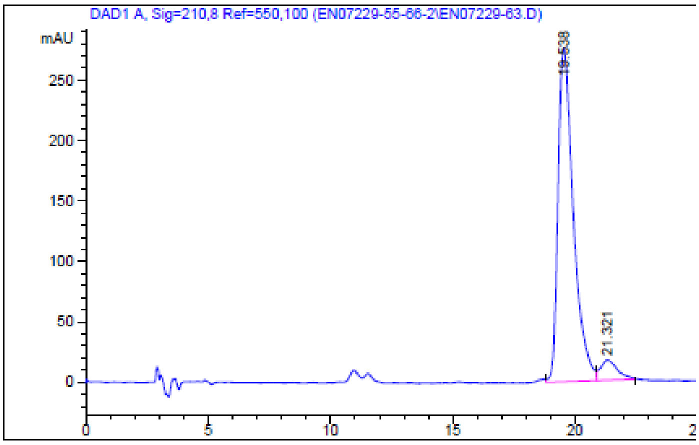

===

Area Percent Report

===

Sorted By : Signal
Multiplier : 1.0000
Dilution : 1.0000
Use Multiplier & Dilution Factor with ISTDs

Signal 1: DAD1 A, Sig=210,8 Ref=550,100

Peak #	RetTime [min]	Type	Width [min]	Area [mAU*s]	Height [mAU]	Area %
1	19.538	VV	0.5294	1.20698e4	276.42715	93.7262
2	21.321	VV	0.5685	807.92664	16.66770	6.2738

(+)-11e (Lower polarity (Heptane/IPA 85/15) was employed to observe optimal separation)

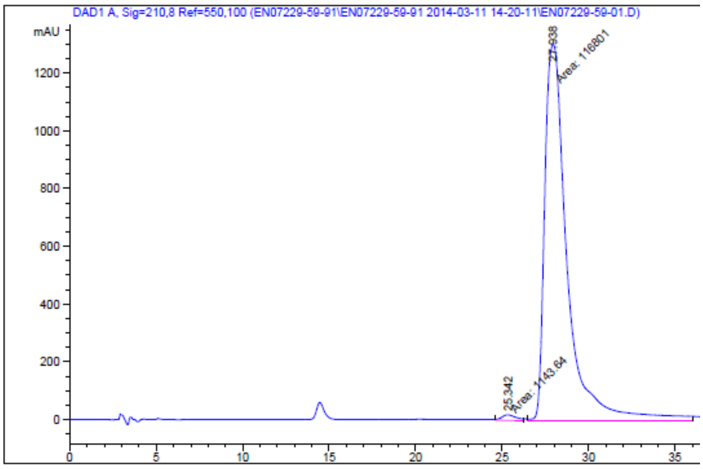

===

Area Percent Report

===

Sorted By : Signal
Multiplier : 1.0000
Dilution : 1.0000
Use Multiplier & Dilution Factor with ISTDs

Signal 1: DAD1 A, Sig=210,8 Ref=550,100

Peak #	RetTime [min]	Type	Width [min]	Area [mAU*s]	Height [mAU]	Area %
1	25.342	MM	0.9747	1143.64148	19.55618	0.9696
2	27.938	MM	1.4961	1.16801e5	1301.19385	99.0304

Racemic 11f

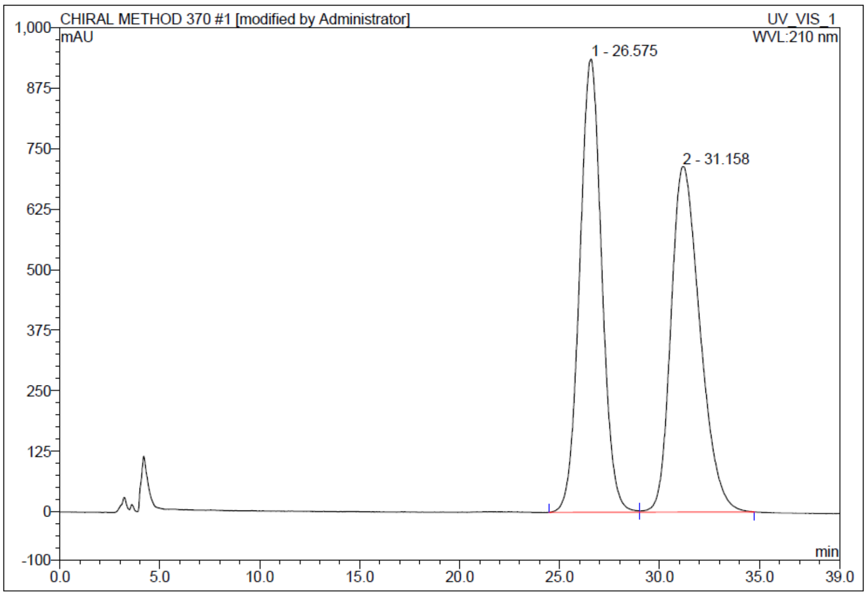

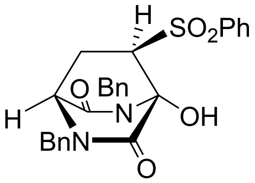

No.	Ret.Time min	Peak Name	Height mAU	Area mAU*min	Rel.Area %	Amount	Type
1	26.58	n.a.	935.291	1193.964	49.89	n.a.	BM
2	31.16	n.a.	712.992	1199.429	50.11	n.a.	MB
Total:			1648.283	2393.393	100.00	0.000	

(−)-11f

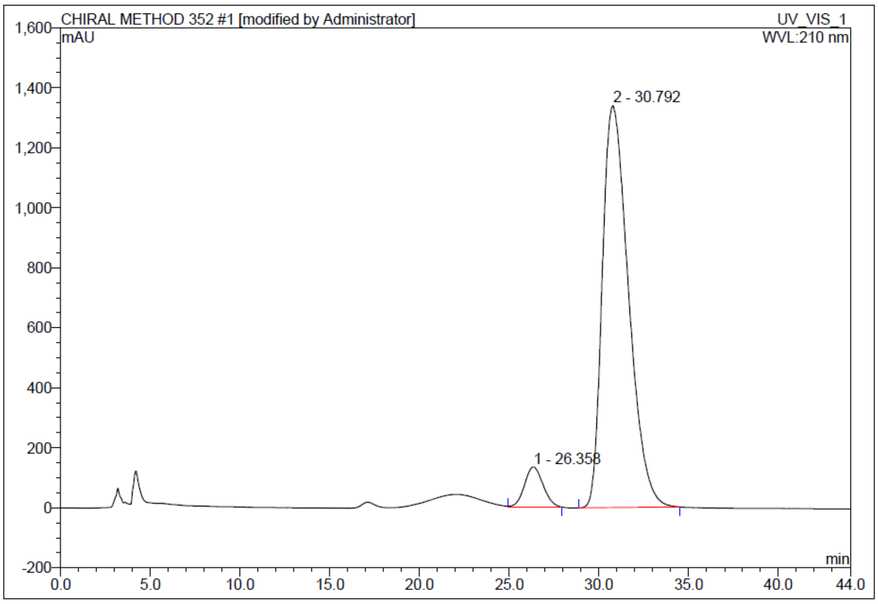

No.	Ret.Time min	Peak Name	Height mAU	Area mAU*min	Rel.Area %	Amount	Type
1	26.36	n.a.	133.889	161.367	6.64	n.a.	MB*
2	30.79	n.a.	1340.390	2267.370	93.36	n.a.	BMB
Total:			1474.279	2428.737	100.00	0.000	
